# Japanese society for cancer of the colon and rectum (JSCCR) guidelines 2024 for the clinical practice of hereditary colorectal cancer

**DOI:** 10.1007/s10147-025-02892-1

**Published:** 2025-11-10

**Authors:** Kohji Tanakaya, Tatsuro Yamaguchi, Keiji Hirata, Masayoshi Yamada, Kensuke Kumamoto, Yasuki Akiyama, Kei Ishimaru, Koichi Okamoto, Yuko Kawasaki, Keigo Komine, Akira Sakamoto, Kunitoshi Shigeyasu, Yoshiko Shibata, Yusaku Shimamoto, Hideki Shimodaira, Shigeki Sekine, Akinari Takao, Misato Takao, Yasuyuki Takamizawa, Yoji Takeuchi, Noriko Tanabe, Fumitaka Taniguchi, Akiko Chino, Hourin Cho, Satoru Doi, Takeshi Nakajima, Sakiko Nakamori, Yoshiko Nakayama, Toshiya Nagasaki, Hisashi Hasumi, Kouji Banno, Takao Hinoi, Kenji Fujiyoshi, Takahiro Horimatsu, Kenta Masuda, Masashi Miguchi, Yusuke Mizuuchi, Yasuyuki Miyakura, Michihiro Mutoh, Takahiro Yoshioka, Shinji Tanaka, Kazuhiro Sakamoto, Kentaro Sakamaki, Michio Itabashi, Hideyuki Ishida, Naohiro Tomita, Kenichi Sugihara, Yoichi Ajioka

**Affiliations:** 1 Department of Surgery, National Hospital Organization Iwakuni Clinical Center, 1-1-1 Atago-Machi, Iwakuni, 740-8510 Japan; 2 Department of Clinical Genetics, Tokyo Metropolitan Cancer and Infectious Diseases Center Komagome Hospital, Tokyo, Japan; 3 Department of Surgery 1, University of Occupational and Environmental Health, Kitakyushu, Japan; 4 Endoscopy Division, National Cancer Center Hospital, Tokyo, Japan; 5 Department of Genome Medical Science and Medical Genetics, Faculty of Medicine, Kagawa University, Kita-Gun, Japan; 6 Division of Gastrointestinal Surgery and Surgical Oncology, Graduate School of Medicine, Ehime University, Toon, Japan; 7 Department of Gastroenterology and Oncology, Tokushima University Graduate School of Medical Science, Tokushima, Japan; 8 College of Nursing, University of Hyogo, Akashi, Japan; 9 Department of Medical Oncology, Tohoku University Hospital, Sendai, Japan; 10 Department of Surgery, Tokyo Metropolitan Cancer and Infectious Diseases Center Komagome Hospital, Tokyo, Japan; 11 Department of Gastroenterological Surgery, Okayama University Graduate School of Medicine, Dentistry, and Pharmaceutical Sciences, Okayama, Japan; 12 Himawari-No-Kai (Sunflower Association), a Patient Advocacy Group for Individuals and Families Affected By Lynch Syndrome, Iwakuni, Japan; 13 Division of Gastroenterology, Department of Internal Medicine, Kobe University Graduate School of Medicine, Kobe, Japan; 14 Division of Medical Oncology, Faculty of Medicine, Tohoku Medical and Pharmaceutical University, Sendai, Japan; 15 Department of Pathology, Keio University School of Medicine, Tokyo, Japan; 16 Department of Gastroenterology, Tokyo Metropolitan Cancer and Infectious Diseases Center Komagome Hospital, Tokyo, Japan; 17 Department of Colorectal Surgery, National Cancer Center Hospital, Tokyo, Japan; 18 Department of Gastroenterology and Hepatology, Gunma University Graduate School of Medicine, Gunma, Japan; 19 Department of Clinical Genetics, Saitama Medical Center, Saitama Medical University, Saitama, Japan; 20 Department of Surgery, Hiroshima City Hospital Organization Hiroshima City Hiroshima Citizens Hospital, Hiroshima, Japan; 21 Department of Gastroenterology, Cancer Institute Hospital, Japanese Foundation for Cancer Research, Tokyo, Japan; 22 Endoscopy Center, Tokyo Medical University Hospital, Tokyo, Japan; 23 Harmony Line (Association for Patients and Families With Familial Adenomatous Polyposis), Osaka, Japan; 24 Division of Hereditary Tumors, Department of Genetic Oncology, Osaka International Cancer Institute, Osaka, Japan; 25 Department of Pediatrics, Shinshu University School of Medicine, Matsumoto, Japan; 26 Department of Gastroenterological Surgery, Saitama Cancer Center, Saitama, Japan; 27 Department of Urology, Yokohama City University, Yokohama, Japan; 28 Center of Maternal -Fetal/Neonatal Medicine, Hiroshima University Hospital, Hiroshima, Japan; 29 Department of Clinical and Molecular Genetics, Hiroshima University Hospital, Hiroshima, Japan; 30 Department of Surgery, Kurume University School of Medicine, Kurume, Japan; 31 Institute for Advancement of Clinical and Translational Science, Kyoto University Hospital, Kyoto, Japan; 32 Department of Obstetrics and Gynecology, Keio University School of Medicine, Tokyo, Japan; 33 Department of Gastroenterological Surgery, Hiroshima Prefectural Hospital, Hiroshima, Japan; 34 Department of Surgery and Oncology, Graduate School of Medical Sciences, Kyushu University, Fukuoka, Japan; 35 Department of Colon and Pelvic Surgery, Cancer Prevention and Genetic Counseling, Tochigi Cancer Center, Utsunomiya, Japan; 36 Department of Molecular-Targeting Prevention, Graduate School of Medical Science, Kyoto Prefectural University of Medicine, Kyoto, Japan; 37 Department of Gastroenterological Surgery, Kochi Health Sciences Center, Kochi, Japan; 38 JA Onomichi General Hospital, Onomichi, Japan; 39 Koshigaya Municipal Hospital, Koshigaya, Japan; 40 Faculty of Health Data Science, Juntendo University, Tokyo, Japan; 41 Saiseikai Kazo Hospital, Kazo, Japan; 42 Department of Digestive Tract and General Surgery, Saitama Medical Center, Saitama Medical University, Kawagoe, Japan; 43 Division of Cancer Treatment , Toyonaka Municipal Hospital, Toyonaka, Japan; 44 Institute of Science Tokyo , Tokyo, Japan; 45 Division of Molecular and Diagnostic Pathology, Graduate School of Medical and Dental Sciences, Niigata University, Niigata, Japan

**Keywords:** Hereditary colorectal cancer, Guidelines, Familial adenomatous polyposis, Lynch syndrome

## Abstract

Approximately 5% of all colorectal cancers have a strong genetic component and are classified as hereditary colorectal cancer (HCRC). Some of the unique features commonly seen in HCRC cases include early age of onset, synchronous/metachronous cancer occurrence, and multiple cancers in other organs. These characteristics require different management approaches, including diagnosis, treatment or surveillance, from those used in the management of sporadic colorectal cancer. Accurate diagnosis of HCRC is essential because it enables targeted surveillance and risk reduction strategies that improve patient outcomes. Recent genetic advances revealed several causative genes for polyposis and non-polyposis syndromes. The Japanese Society for Cancer of the Colon and Rectum (JSCCR) first published guidelines for the management of HCRC in 2012, with subsequent revisions every 4 years. The 2024 update to the JSCCR guidelines for HCRC was developed by meticulously reviewing evidence from systematic reviews and the consensus of the JSCCR HCRC Guidelines Committee, which includes representatives from patient advocacy groups for FAP and Lynch syndrome. These guidelines provide an up-to-date summary of HCRC, along with clinical recommendations for managing FAP and Lynch syndrome.

## Introduction

### Guideline objective

The number of patients with colorectal cancer (CRC) has been increasing in Japan, and societal interest is high given that it is one of the most common types of cancer. Most CRCs are thought to arise from the accumulation of gene variants in the colonic mucosa caused by the effects of lifestyle, environmental factors, and aging. Between 20 and 30% of all CRCs develop in relatives (i.e., familial clustering) and are sometimes called familial CRCs. A causative gene is identified in up to 5% of CRCs, regardless of familial clustering, and these cases are collectively referred to as hereditary colorectal cancer (HCRC). HCRC tends to be complicated by a young onset, synchronous/metachronous CRC, and multiple cancers in other organs. Therefore, it is necessary to take measures different from those for sporadic CRC. However, awareness of HCRC is not always high among clinicians.

The most common HCRCs are familial adenomatous polyposis (FAP) and Lynch syndrome. FAP is diagnosed by the characteristic clinical finding of colorectal adenomatous polyposis. However, recent investigations have found that colonic adenomatous polyposis can be caused by several genes other than *APC*, which is the causative gene for FAP. Therefore, genetic testing, rather than relying on clinical findings alone, is recommended for accurate diagnosis of colorectal adenomatous polyposis. Furthermore, although the frequency of Lynch syndrome is higher than that of FAP, many cases of Lynch syndrome are overlooked and undiagnosed because of the lack of characteristic clinical features. In recent years, however, with the spread of companion diagnoses and comprehensive genome profiling tests, Lynch syndrome is increasingly being suspected and diagnosed.

Against this backdrop, the Japanese Society for Cancer of the Colon and Rectum (JSCCR) guidelines 2024 for the Clinical Practice of Hereditary Colorectal Cancer (“2024 JSCCR Guidelines for HCRC” or “these guidelines” below) were developed with four aims in mind: (1) to deepen the understanding of the concept of HCRC, (2) to provide guidance on management strategies for HCRC, including diagnosis and surveillance, (3) to emphasize the importance of considering the psychosocial burden caused by hereditary diseases and the need to provide support for patients and their families, and (4) to promote mutual understanding by healthcare professionals and patients by making these guidelines available to the public.

Inclusion of Lynch syndrome in these guidelines may be considered inappropriate given the diversity of these tumors. However, in view of the history of the creation of these guidelines, this question will be left for future examination and revision.

### Principles underlying development of these guidelines

These guidelines provide evidence in support of clinical strategies for the diagnosis, treatment, and surveillance of HCRC but do not discuss treatment methods. We have attempted to develop these guidelines in accordance with the concept of evidence-based medicine. However, the incidence of HCRC is relatively low, and it is difficult to design studies that can provide a high level of evidence. Therefore, the guidelines have been developed by consensus among members of the JSCCR based on information obtained from a literature search and considering the health insurance system and actual clinical practice in Japan. Moreover, considering the specific characteristics of HCRC, members of the Japanese Society for Hereditary Tumors and patient/family associations also participated in the Guideline Development Committee. This committee comprised specialists in internal medicine, surgery, gynecology, pediatrics, pathology, genetic diagnosis, genetic counseling, and nursing, as well as representatives of patient/family associations.

### Topics

The main topics addressed in these guidelines are the diagnosis, treatment, and surveillance of HCRCs (FAP and Lynch syndrome) and genetic counseling for patients with these diseases in Japan.

### Perspectives

These guidelines adopted an individual perspective.

### Users and facilities

The guidelines are intended for use by physicians, nurses, genetic counselors, and experts in genetic medicine engaged in the diagnosis and management of HCRCs and related conditions in hospitals, clinics, and screening facilities.

### Scope of coverage

The guidelines cover patients and families with FAP, Lynch syndrome, and related HCRCs. Sporadic (non-hereditary) CRC, hamartomatous polyposis syndromes (Peutz-Jeghers syndrome, juvenile polyposis syndrome, Cowden syndrome/*PTEN* hamartoma tumor syndrome) and serrated polyposis syndrome are excluded.

### Collection of evidence

Evidence was collected by a literature search conducted in September 2022 using PubMed, Ichushi-Web (Japan Medical Abstracts Society), and the Cochrane Library to develop the Clinical Questions (CQ).

### Evaluation of evidence

We comprehensively collected studies related to the CQs and grouped the evidence presented in individual papers with respect to critical outcomes by study design. Individual studies were then assessed for risk of bias, indirectness, inconsistency, imprecision, and publication bias according to the study design (Table 1). Next, as in the 2022 JSCCR guidelines for the treatment of CRC [1], we assessed the body of evidence at the literature level according to the Grading of Recommendations Assessment, Development and Evaluation (GRADE) system [2] and finally determined the evidence level for the CQs. The evidence level is described at four levels: “A: There is strong confidence in the estimated values of the effect”; “B: There is moderate confidence in the estimated values of the effect”; “C: There is limited confidence in the estimated values of the effect”; and “D: There is little confidence in the estimated values of the effect” (Table 2).

**Table 1:** Judgment about the quality of the underlying evidence

1. Initial study design
Level A: systematic review, metanalysis, or randomized control study
Level B: other
Level C: observational study, cohort study, or case–control study
Level D; case series, or case report
2. Factors that can reduce the quality of the evidence
Limitations in study design or execution (risk of bias)
Inconsistency of results
Indirectness of evidence
Imprecision
Publication bias
3. Factors that can increase the quality of the evidence
Large magnitude of effect
All plausible confounding would reduce the demonstrated effect or increase the effect if no effect was observed
Dose–response gradient
4. The final total evidence was determined by evaluating the above in the order of 1, 2, and 3

**Table 2:** Definition of the evidence level for CQs

Evidence level A (high)	We are very confident that the true effect lies close to that of the
Evidence level B (moderate)	estimate of the effect We are moderately confident in the effect estimate: The true effect is likely to be close to the estimate of the effect, but there is a possibility that it is substantially different
Evidence level C (low)	Our confidence in the effect estimate is limited: The true effect may be substantially different from the estimate of the effect
Evidence level D (very low)	We have very little confidence in the effect estimate: The true effect is likely to be substantially different from the estimate of effect

### Strength of recommendations

Draft recommendations were developed based on the outcomes and evidence levels generated as described above and were assessed at a consensus meeting convened by members of the Guideline Development Committee. In the CQ text, the determined recommendations were expressed directly, and inconsistencies were removed. HCRC is an area with limited high-level evidence, except for chemoprevention. Therefore, local and international guidelines were referenced to determine the strength of the recommendations. Insurance coverage was not considered in determining the strength of the recommendations. The strength of the draft recommendations was determined by voting according to the GRADE Grid method, which assesses the following four items: certainty of evidence, patients’ preferences, benefits and harms, and costs (Table 3).

**Table 3:** Strength of CQ recommendations

Recommendations	
1 (Strong recommendation)	Strong “For” an intervention
	Strong “Against” an intervention
2 (Weak recommendation)	Weak “For” an intervention
	Weak “Against” an intervention

#### Voting method

1. Select one of the following five options:
  1. Strong “For” intervention
  2. Weak “For” intervention
  3. Weak “Against” intervention
  4. Strong “Against” intervention
  5. Not graded
2. If ≥ 70% of all votes agreed on one of the options (1)–(5) in the first vote, that option was chosen by consensus. This condition did not apply in the case of the following:
  1. If (1) + (2) exceeds 50% and (3) + (4) is 20% or lower, “weakly recommend to perform” is selected
  2. If (3) + (4) exceeds 50% and (1) + (2) is 20% or lower, “weakly recommend not to perform” is selected
3. If neither of the two conditions were met at the time of the first vote, a second discussion was held under the category of “no consensus was reached,” while taking into account the medical circumstances in Japan and disclosing the voting results; a second round of voting was then held.
4. If no consensus was reached in the second vote, “Not graded” was selected.

Representatives of the patient/family association also voted on the CQs after receiving an interpretation of the guidelines and freely expressing their opinions at the meeting. In brief, a total of 18 committee members participated in the voting for each CQ. Among them, one representative of the patient/family association voted for the CQ relevant to his or her condition (i.e., either familial adenomatous polyposis or Lynch syndrome).

### Explanatory text

Attempts were made to use clear and unambiguous language in the CQs. Ease of understanding and avoidance of convoluted text were emphasized in the CQ explanations. Descriptions of specific figures and values in the research results when referring to a large number of clinical trials were abbreviated as appropriate. The study design based on which the recommendations were to be determined was specified whenever possible, including meta-analyses, randomized controlled trials, and observational studies.

### Chapters

In terms of the extraction of key clinical issues, well-established evidence has been described in each chapter, which include “I. Overview of hereditary colorectal cancers,” “II. Familial adenomatous polyposis,” and “III. Lynch syndrome.” Clinical topics for which insufficient data were available from the literature search are also included in each chapter. The outline, diagnosis, treatment, and surveillance of these diseases are described with the abundant use of flowcharts, figures, and tables. Easy-to-understand explanations are added as side notes when possible, given the specific characteristics of HCRC, to ensure correct understanding of the disease characteristics and terminology.

## Chapter I. Overview of hereditary colorectal cancers

### Basic concepts

#### Definition

Hereditary colorectal cancers (HCRCs) are disorders for which causative genes have been identified that increase the risk of colorectal cancer (CRC). In HCRCs, the mode of inheritance, density of colorectal polyps, histological type, risk of CRC, and extra-colorectal manifestations vary depending on the causative gene. Table 4 summarizes the molecular genetic characteristics and clinicopathological features of the representative HCRCs.

**Table 4:** Genetic and clinical characteristics of representative hereditary colorectal cancers. *FAP*, familial adenomatous polyposis; *AFAP*, attenuated familial adenomatous polyposis; *GAPPS*, gastric adenocarcinoma and proximal polyposis of the stomach; *MAP*, *MUTYH*-associated polyposis; *PPAP*, polymerase proofreading-associated polyposis; *CMMRD*, constitutional mismatch repair deficiency; *PJS*, Peutz-Jeghers syndrome; *JPS*, juvenile polyposis syndrome; *HMPS*, hereditary mixed polyposis syndrome; *CS/PHTS*, Cowden syndrome/*PTEN* hamartoma tumor syndrome; *SPS*, serrated polyposis syndrome; *AD*, autosomal dominant; *AR*, autosomal recessive; *NA*, not applicable

Polyposis	Disorder	mode of inheritance	Causative gene	Prevalence	Number of colonic polyps	Histology	Risk of colorectal cancer	Extra-colonic polyposis	Extra-colonic neoplasms
Adenomatous polyposis	FAP	AD	*APC*	1/20,000 ~ 1/10,000	> 100	adenoma	100%	stomach, duodenum	gastric cancer, duodenal (papillary) cancer, desmoid tumor, papillary thyroid cancer, brain tumor, hepatoblastoma, etc
	AFAP	AD	*APC*		> 10, < 100	adenoma	70%		
	GAPPS	AD	*APC* (1B)	NA	–	adenoma	–	stomach	gastric cancer
	MAP	AR	*MUTYH*	NA	< 100	adenoma, HP	70 ~ 90%	duodenum	duodenal (papillary) cancer, papillary thyroid cancer, sebaceous tumor, etc
	PPAP	AD	*POLD1*	NA	> 30, < 100	adenoma	> 20%	duodenum	endometrial cancer, breast cancer, brain tumor
	PPAP	AD	*POLE*	NA	> 30, < 100	adenoma	> 20%	–	duodenal cancer, brain tumor
	CMMRD syndrome	AR	*MLH1, MSH2, MSH6, PMS2*	1/1,000,000 ~ 3/1,000,000	> 10, < 100	adenoma	80 ~ 95%	–	hematological tumor, brain tumor
	*MSH3*-associated polyposis	AR	*MSH3*	NA	> 30, < 100	adenoma	–	duodenum	goiter, intraductal papilloma, gastric cancer, brain tumor, etc
	*MLH3*-associated polyposis	AR	*MLH3*	NA	> 30, < 100	adenoma	–	–	–
	*NTHL1*-associated polyposis	AR	*NTHL1*	NA	> 1, < 100	adenoma	> 20%	–	breast cancer, endometrial cancer, bladder cancer, HNSCC, skin cancer, etc
	*AXIN2*-associated polyposis	AD	*AXIN2*	NA	< 100	adenoma	–	–	–
Hamartomatous polyposis	PJS	AD	*STK11*	1/280,000 ~ 1/8,300	> 5	hamartoma	39%	stomach, small bowel	gastric cancer, small bowel cancer, pancreatic cancer, breast cancer, adenocarcinoma of the cervix, etc
	JPS, HMPS	AD	*BMPR1A*	1/100,000 ~ 1/16,000	> 5	hamartoma	40 ~ 50%	stomach, small bowel	gastric cancer, small bowel cancer, pancreatic cancer, etc
	JPS	AD	*SMAD4*		> 5	hamartoma	< 50%	stomach, small bowel	
	CS/PHTS	AD	*PTEN*	1/200,000	< 100	hamartoma	11 ~ 20%	stomach, small bowel	breast cancer, endometrial cancer, thyroid follicular carcinoma, renal cell carcinoma, etc
Serrated polyposis	SPS	AD	*RNF43*	NA	> 5, < 100	SL	–	–	–
Mixed polyposis	HMPS	AD	*GREM1*	NA	–	adenoma, HP, inflammatory	11 ~ 20%	–	–
Non-polyposis	LS	AD	*MLH1*	1 ~ 4% of colorectal cancer	–	adenoma, SSL	46 ~ 61%	–	endometrial cancer, gastric cancer, ovarian cancer, small bowel cancer, biliary tract cancer, pancreatic cancer, upper urinary tract cancer, brain tumor, sebaceous tumor, etc
		AD	*MSH2*		–	adenoma, SSL	33 ~ 52%	–	
		AD	*MSH6*		–	adenoma, SSL	10 ~ 44%	–	
		AD	*PMS2*		–	adenoma, SSL	8.7 ~ 20%	–	
		AD	*EPCAM*		–	adenoma	33 ~ 52%	–	
	LFS	AD	*TP53*	NA	–	–	> 20%	–	bone and soft tissue sarcoma, adrenocortical tumor, brain tumor, leukemia, breast cancer, etc
	BHDS	AD	*FLCN*	NA	–	–	–	–	renal cell carcinoma
	–		*MUTYH*	NA	–	–	10 ~ 13%	–	–
	–		*ATM*	NA	–	–	5 ~ 10%	–	–
	–		*BLM*	NA	–	–	5 ~ 10%	–	–
	–		*CHEK2*	NA	–	–	5 ~ 10%	–	–
	–		*GALNT12*	NA	–	–	5 ~ 10%	–	–
	–		*RPS20*	NA	–	–	–	–	–

#### Classification

HCRCs are classified into two groups, namely, polyposis and non-polyposis. HCRCs with polyposis are further classified into adenomatous, hamartomatous, and serrated polyposis according to histological type.

- Adenomatous polyposis is defined as ≥ 10 adenomatous polyps in the colon and rectum. It is sometimes classified as severe/profuse/dense type (≥ 1,000–2,000), sparse type (100–1,000), or attenuated type (10–100) based on the number of polyps. However, the number of polyps observed depends on the patient's age, the use of high-resolution endoscopy, chromoendoscopy, and image enhanced endoscopy, including spraying dye and narrow-band imaging, and the physician. Patients with thousands of adenomatous polyps are often diagnosed with familial adenomatous polyposis (FAP), which frequently involves gastric and duodenal polyps. In patients with an attenuated type, it is difficult to estimate the causative gene because there are numerous conditions characterized by the development of 10–100 polyps, including FAP, *MUTYH*-associated polyposis, polymerase proofreading-associated polyposis, constitutional mismatch repair deficiency syndrome, *MSH3*-associated polyposis, *MLH3*-associated polyposis, and *NTHL1*-associated polyposis. Furthermore, these HCRCs sometimes involve more than 100 polyps. Therefore, it is difficult to diagnose HCRC as adenomatous polyposis based on the number of polyps alone. In terms of the mode of inheritance, adenomatous polyposis caused by a germline pathogenic variant (GPV) in any of the *APC*, *POLE*, *POLD1*, or *AXIN2* genes is inherited in an autosomal dominant manner, whereas other adenomatous polyposis conditions are inherited in an autosomal recessive manner.
- Hamartomatous polyposis syndrome occasionally involves fewer than 10 colorectal polyps. Representative hamartomatous polyposis syndromes include Peutz-Jeghers syndrome, juvenile polyposis syndrome, and Cowden syndrome/*PTEN* hamartoma tumor syndrome, which are distinguished by histological type. Patients with hamartomatous polyposis syndrome develop multiple polyps in the stomach and small intestine (including the duodenum) and other features, such as lip pigmentation or macrocephaly, depending on the syndrome [1–3].
- Serrated polyposis syndrome includes the development of various types of polyps, including hyperplastic polyps and serrated lesions. Some patients with this syndrome have GPVs of *RNF43*, *GREM1*, and *MUTYH* [4–6]; however, the causative genes are not well defined. Environmental factors may also be involved in the development of serrated polyps.

HCRCs without polyposis include Lynch syndrome and Li–Fraumeni syndrome, neither of which has a characteristic appearance. Therefore, patients with these syndromes cannot be diagnosed with HCRC based on clinicopathological factors.

#### Epidemiology

**Fig. 1:**
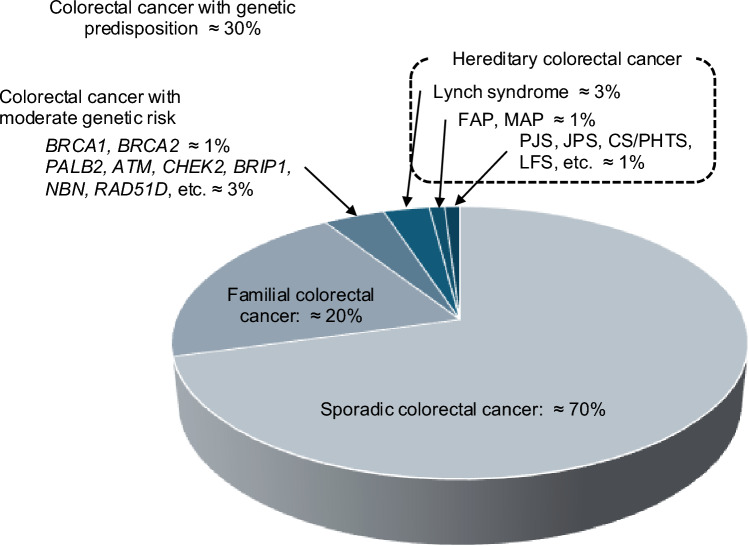
Percentage of genetically predisposed colorectal cancers among all colorectal cancers. FAP, familial adenomatous polyposis; CS/PHTS, Cowden syndrome/*PTEN* hamartoma tumor syndrome; LFS, Li-Fraumeni syndrome; JPS, juvenile polyposis syndrome; MAP, *MUTYH*-associated polyposis; PJS, Peutz-Jeghers syndrome

Approximately 30% of patients with CRC have familial clustering of CRC or GPVs of cancer predisposition genes (Fig. 1). Patients with autosomal recessive inheritance, somatic mosaicism, or autosomal dominant inheritance de novo do not have familial clustering of CRC.

According to studies of germline multi-gene panel testing (^*^MGPT) for CRC patients, FAP accounts for 0.5% and Lynch syndrome for 3% of cases, with GPVs in *ATM*, *CHEK2*, *MUTYH*, and *TP53* also observed, resulting in approximately 5% of CRCs being HCRC [7, 8]. Furthermore, *BRCA1* and *BRCA2*, which are causative genes for hereditary breast and ovarian cancers, have each been detected in 1% of patients, and *PALB2*, *BRIP1*, *NBN*, and *RAD51D* were detected in 3% overall. Therefore, approximately 10% of patients with CRC are estimated to have a hereditary cancer predisposition syndrome.

^*^Unless otherwise specified, “MGPT” refers to “germline MGPT.”

#### Risk of tumorigenesis

The risk of developing CRC depends on the type of HCRC. Not all patients with genetic predispositions will develop CRC, with the exception of typical FAP, which has almost 100% penetrance. Furthermore, the risk of developing CRC depends on the causative gene, even within the same syndrome, as in Lynch syndrome [9].

Patients with HCRC are also at high risk of developing extra-colorectal tumors or lesions, known as associated lesions. FAP-associated tumors (lesions) include fundic gland polyposis and duodenal adenoma, while Lynch syndrome is associated with gynecological and urological tumors. These associated lesions develop in an organ-specific manner. Moreover, some patients with HCRC develop associated lesions before development of CRC. For example, approximately 35% of women with Lynch syndrome reportedly develop endometrial cancer as a sentinel cancer [10, 11]. Therefore, a multidisciplinary approach involving gastroenterology, colorectal surgery, and other departments is critical in the surveillance of HCRC.

#### Mechanisms of carcinogenesis

All of the causative genes for HCRC identified to date are tumor suppressor genes, and their functions include inhibiting cell proliferation (e.g., *APC*, *TP53*, *PTEN*, and *SMAD4*) or DNA repair (e.g., *MLH1*, *MSH2*, and *MUTYH*). Thus, variants considered pathogenic in HCRC are those that result in loss of function of the causative genes, including truncating variants, loss of heterozygosity, large duplications/deletions, and pathogenic missense variants.

**Fig. 2:**
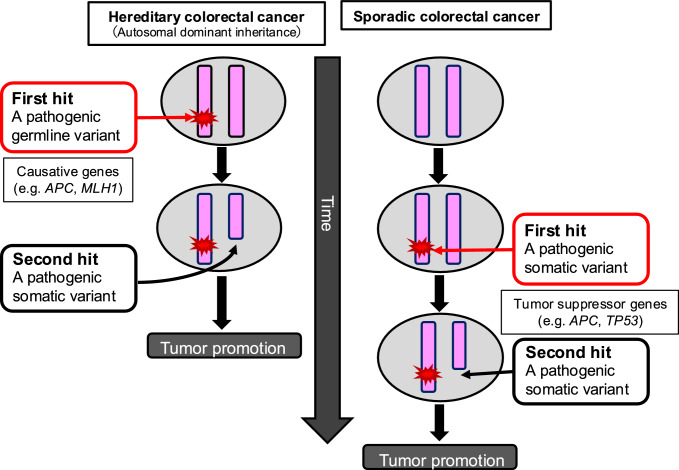
Tumorigenesis mechanism in the two-hit theory of tumor suppressor genes based on Knudson's two-hit theory

In autosomal dominant HCRCs, in addition to the GPV of the causative gene, an acquired pathogenic variant in the wild-type allele results in loss of function of the corresponding protein, causing the associated tumor [12]. Multiple tumors develop at a younger age in patients with HCRC than in those with sporadic CRC because all the cells carry a pathogenic variant in one allele of the causative gene (Fig. 2).

**Fig. 3:**
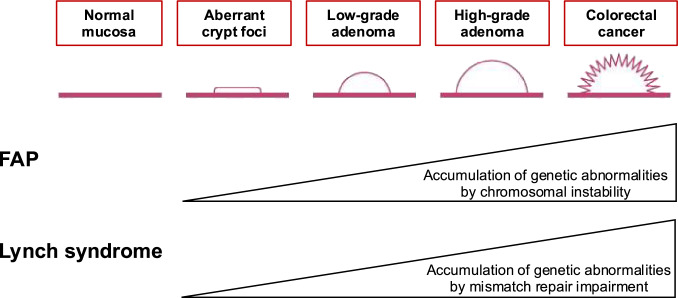
Representative tumorigenesis mechanisms in familial adenomatous polyposis and Lynch syndrome. FAP, familial adenomatous polyposis

Development of CRC in FAP is explained by the multistep model of carcinogenesis. Initially, loss of function of the APC protein in colonic epithelial cells results in accumulation of β-catenin in the cytoplasm, which increases its translocation into the nucleus, where it forms a complex with TCF4, promoting transcription of oncogenes leading to cell proliferation. Morphologically, this process is thought to give rise to aberrant crypt foci as the initial lesion. Additional genetic alterations, such as those in *KRAS* and *TP53*, are required for progression from aberrant crypt foci to adenoma and eventually to CRC. This process is associated with a condition known as chromosomal instability, which predisposes cells to genetic abnormalities (Fig. 3).

Development of CRC in Lynch syndrome involves disruption of the mismatch repair system. DNA replication during cell division is highly accurate, with an error rate of 10^–10^ to 10^–8^. However, in the event of replication errors, mismatch repair proteins encoded by the causative genes for Lynch syndrome identify these errors, including mismatches and small insertions/deletions. When an additional acquired pathogenic variant occurs alongside the GPV in a mismatch repair gene, the mismatch repair system becomes defective, losing its ability to detect replication errors. These replication errors frequently occur in microsatellite regions, which are repetitive sequences of up to a few bases within the genome. Some genes containing these repetitive sequences code for proteins involved in tumor suppression (e.g., *TGFBR2*), cellular proliferation, DNA repair (e.g., *MSH3*, *MSH6*), and apoptosis (e.g., *BAX*). Disruption of the mismatch repair system leads to microsatellite instability (MSI), resulting in accumulation of variants in these genes and development of tumors.

Autosomal recessive HCRCs increase the risk of developing cancer when each of the alleles with a GPV is inherited from parents who are carriers of a GPV of the causative gene. The causative genes for autosomal recessive HCRC are involved in DNA repair, so tumors develop because of the inability to repair certain types of DNA damage.

### Diagnosis

#### Significance of diagnosis

HCRCs are diagnosed by genetic testing because some HCRCs have a similar phenotype. However, some of the HCRCs can be diagnosed by their characteristic phenotype. The mode of inheritance, penetrance of malignant tumors, associated tumors (lesions), and surveillance depend on the causative gene. The diagnosis is useful for risk assessment and estimation of the risk of recurrence in relatives.

Genetic counseling should be provided at appropriate times during genetic testing and diagnosis and involves the provision of information as well as psychological and social support that allows patients and clinicians to make informed choices. This process is best performed as a team effort by experienced physicians and professionals skilled in genetic counseling [13]. Providing detailed information on the suspected disease and the benefits and limitations of testing is essential before proceeding with genetic tests.

#### Diagnostic process

**Fig. 4:**
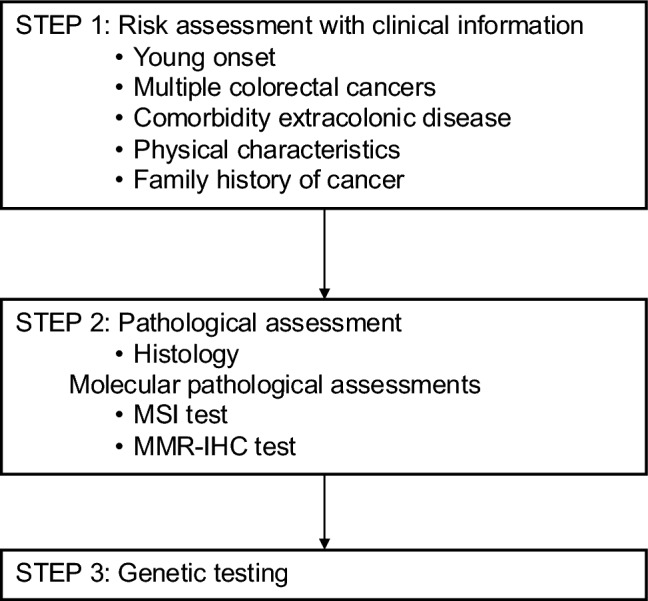
Risk assessment of hereditary colorectal cancer. MMR-IHC, mismatch repair-immunohistochemistry; MSI, microsatellite instability

HCRC is diagnosed in a three-step process: risk assessment based on clinical information (step 1), pathological and molecular pathological assessments (step 2), and genetic testing (step 3) (Fig. 4).

Step 1: Risk assessment based on clinical information

The first step in the diagnosis of HCRC is risk assessment based on clinical information, including (1) early onset, (2) synchronous/metachronous CRC, (3) history of malignancy other than CRC, (4) physical features, and (5) a maternal and paternal family history of up to a third-degree relative (at least second-degree relative) with CRC (Fig. 4).

Patients with HCRC often develop extra-colorectal lesions, which may include dental abnormalities, lip pigmentation, and macrocephaly, findings of which help with diagnosis and estimation of the causative gene. The current trend toward nuclear families may make it difficult to obtain information concerning even second-degree relatives. Therefore, we recommend collecting the information needed to distinguish the paternal and maternal sides to create a pedigree and assess the mode of inheritance. The presence of associated tumors is a key point in screening, particularly in patients with HCRC without polyposis.

Endoscopic findings in the gastrointestinal tract are crucial in the diagnosis of HCRC. Therefore, the number, type, and location of polyps detected by both lower and upper gastrointestinal endoscopy should be accurately recorded.

If HCRC is suspected based on clinical information, clinicians should proceed to step 2 and then to step 3 if a patient with CRC has a family member already diagnosed with HCRC and their phenotype matches that of the disease [14].

Step 2: Pathological and molecular pathological assessments

**Fig. 5:**
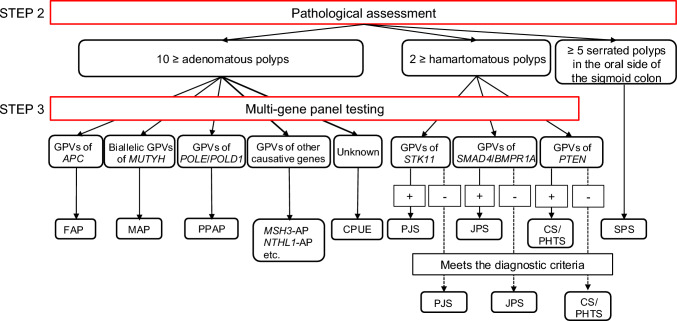
Flowchart of the diagnosis of hereditary colorectal polyposis. *GPVs*, germline pathogenic variants; *FAP*, familial adenomatous polyposis; *MAP*, *MUTYH*-associated polyposis; *PPAP*, polymerase proofreading-associated polyposis; *MSH3*-*AP*, *MSH3*-associated polyposis; *NTHL1*-*AP*, *NTHL1*-associated polyposis; *CPUE*, colonic adenomatous polyposis of unknown etiology; *PJS*, Peutz-Jeghers syndrome; *JPS*, juvenile polyposis syndrome; *HMPS*, hereditary mixed polyposis syndrome; *CS/PHTS*, Cowden syndrome/*PTEN* hamartoma tumor syndrome; *SPS*, serrated polyposis syndrome

Patients with polyposis should undergo biopsy for pathologic assessment. The process for diagnosing hereditary colorectal polyposis based on histological findings is summarized in Fig. 5 and Table 5. Each type of polyposis, including adenomatous, hamartomatous, and serrated, has several differential conditions and candidate causative genes.

**Table 5:** Classification of polyposis and diseases to be differentiated

Colonoscopy findings	Histology	Number of polyps	Diseases to be differentiated	Candidate causative genes
Adenomatous polyposis	Adenoma	> 10	FAP, MAP, PPAP, CMMRD syndrome	*APC, MUTYH, POLD1, POLE, MLH1, MSH2, MSH6, PMS2, MSH3, MLH3, NTHL1, AXIN2*
Hamartomatous polyposis	Hamartoma	> 2	PJS, JPS, CS/PHTS, HMPS	*STK11, BMPR1A, SMAD4, PTEN*
Serrated polyposis	HP, SSL	> 5 in the oral side of the sigmoid colon	SPS, MAP	*RNF43, MUTYH, GREM1*
Mixed polyposis	JP, HP, SSL		HMPS, JP	*GREM1, BMPR1A*

PJP, Peutz–Jeghers polyp; JP, juvenile polyp; HP, hyperplastic polyp; SSL, sessile serrated lesion; FAP, familial adenomatous polyposis; MAP, *MUTYH*-associated polyposis; PPAP, polymerase proofreading-associated polyposis; CMMRD, constitutional mismatch repair deficient; PJS, Peutz–Jeghers syndrome; JPS, juvenile polyposis syndrome; HMPS, hereditary mixed polyposis syndrome; CS, Cowden syndrome; PHTS, *PTEN* hamartoma tumor syndrome; SPS, serrated polyposis syndrome

Patients with a non-polyposis syndrome should undergo MSI or mismatch repair immunohistochemistry testing using CRC tissue, given that most CRCs associated with Lynch syndrome show MSI-High or loss of expression of mismatch repair proteins corresponding to the causative gene. However, regardless of the results, clinicians should proceed to step 3 if the patient’s family history indicates a high incidence of Lynch syndrome-associated tumors.

Step 3: Genetic testing

Genetic testing for diagnosis of HCRC involves analysis of DNA extracted from whole blood to detect the GPV of the causative gene, which allows estimation of the cancer risk. The results are useful for informed consent and surveillance [15, 16] and can also be used to diagnose relatives.

Genetic testing for HCRC includes (1) direct sequencing, (2) multiplex ligation-dependent probe amplification (MLPA), (3) targeted sequencing by MGPT, (4) whole-exome sequencing, and (5) whole-genome sequencing (Table 6).

**Table 6:** Types of germline genetic tests for diagnosis of hereditary colorectal cancer

Direct sequencing (Sanger sequencing)
e.g., Diagnosis of relatives, including asymptomatic individuals (Relative Diagnosis)
Multiplex Ligation-dependent Probe Amplification(MLPA)
e.g., Diagnosis of relatives, including asymptomatic individuals (Relative Diagnosis)
Multi-gene panel testing (Target sequencing)
e.g., *MLH1, MSH2, MSH6, PMS2, EPCAM* for Lynch syndrome e.g., *APC, MUTYH, POLD1, POLE, MLH1, MSH2, MSH6, PMS2, MSH3, MLH3, NTHL1, AXIN2* for adenomatous polyposis
Whole exome sequencing
e.g., Genetic testing for colorectal cancer patients with atypical phenotypes
Whole genome sequencing
e.g., Genetic testing for colorectal cancer patients suspected of having inversions or translocations in causative genes that result in pseudo-exon generation

Direct sequencing reads the base sequence of the exons and exon–intron boundary regions encoding the protein of the suspected causative gene one base at a time. MLPA detects large deletions or duplications by adding specific probes to each exon. Direct sequencing or MLPA is used when the causative gene is strongly suspected. However, pathogenic variants in *APC* may not be detected, even in cases with thousands of adenomatous polyps. For diagnosis of relatives, it is sufficient to analyze only the pathogenic variant identified in the proband (single-site analysis); therefore, direct sequencing or MLPA is used depending on the variant to be detected. Targeted sequencing by MGPT generates a panel of candidate causative genes for HCRC, which are then analyzed by next-generation sequencing. Targeted sequencing by MGPT is now widely used because multiple causative genes are often suspected based on clinical findings in HCRCs. Genes should be analyzed such as *APC*, *MUTYH*, *POLD1*, and *POLE* for adenomatous polyposis and *MLH1*, *MSH2*, *MSH6*, *PMS2*, and *EPCAM* for Lynch syndrome. Targeted sequencing allows the simultaneous analysis of multiple genes, with the addition of *BRCA1/2* to MGPT for diagnosis of hereditary breast and ovarian cancers [17, 18]. Whole-exome sequencing analyzes all exons and exon–intron boundary regions of all genes, whereas whole-genome sequencing analyzes all regions, including introns, using next-generation sequencing. Therefore, these sequences sometimes detect hereditary diseases unrelated to tumor development. Whole-genome sequencing can also detect genetic alterations, such as translocations or inversions, that are difficult to detect using other sequencing methods.

In HCRC with somatic mosaicism, only some cells in the body have pathogenic variants, potentially resulting in variant frequencies that fall below the detection sensitivity of direct sequencing. However, next-generation sequencing can detect variants at low frequencies, making it useful for diagnosing somatic mosaicism.

MGPT has been reported to identify GPVs in approximately 20% of patients with CRC under 50 years of age [19]. The National Comprehensive Cancer Network Guidelines (version 2. 2023) [9] recommend MGPT-based targeted sequencing for all patients with CRC under 50 years of age, whereas MSI-High/deficient mismatch repair (dMMR) is recommended for patients older than 50 years. Therefore, genetic testing using targeted sequencing by MGPT is highly recommended for suspected cases of HCRC.

#### Germline (secondary) findings in cancer genome profiling tests

In some patients, HCRCs are diagnosed or suspected based on germline findings in comprehensive cancer genomic profiling tests. Pathogenic variants identified in normal samples are considered to be GPVs. Genetic testing is required when presumed GPVs are detected in tumor-only testing.

#### Interpretation of genetic test results

The clinical relevance of detected variants is generally assessed using classifications from ClinVar (https://www.ncbi.nlm.nih.gov/clinvar/) or InSiGHT (https://www.insightgroup.org/variants/databases/) (Table 7).

**Table 7:** Classification of Gene Variants in Public Databases

ClinVar/Clinical significance value	InSiGHT/MMR gene variant classification criteria
Pathogenic	Class 5 Pathogenic
Likely pathogenic	Class 4 Likely pathogenic
Uncertain significance	Class 3 Uncertain significance
Likely benign	Class 2 Likely not pathogenic/little clinical significance
Benign	Class 1 Not pathogenic/no clinical significance

InSiGHT, International Society for Gastrointestinal Hereditary Tumours; MMR, mismatch repair

A) “Pathogenic variant or likely pathogenic variant”.

The genetic disorder is managed medically. However, it should be understood that individuals who have a pathogenic variant will not always develop cancer during their lifetime, except for diseases that have nearly 100% penetrance, such as FAP.

B) “Variants of uncertain significance were detected”.

Genetic changes with unknown implications for the development of disease are reported as variants of uncertain significance. Examples include silent variants (where a single change in the base sequence does not affect amino acid synthesis) or missense variants (where amino acid substitution occurs), which do not affect the likelihood of disease development. In such cases, the recommendation is to proceed in accordance with the following section on “Genetic abnormalities are not detected” until the significance of this variant is known.

C) “Genetic abnormalities are not detected”.

When no genetic abnormalities are confirmed in the family.

If the same genetic changes are not found in other members of a pedigree when a definitive diagnosis is obtained by genetic testing, it is determined that it is not a genetic disease. However, the risk of tumorigenesis in the general population should still be understood in such cases.

When no diagnosis is made in the family

Given that some variants cannot be detected by genetic testing or may result from abnormalities in unknown causative genes, these cases should be treated with caution. For example, the clinical detection rate of pathogenic variants of APC even in FAP with 100–1000 polyps does not reach 100%, with values of approximately 60% [20]. Therefore, the possibility that unknown variants have not been detected because of technical problems or the presence of other gene abnormalities must be considered. In clinical practice, such cases should ideally be managed in the same way as patients with diseases that are considered to be hereditary, even when no pathogenic variant is detected by genetic testing.

### Genetic counseling

#### Definition

Genetic counseling is the process of helping individuals to understand and adjust to the medical, psychological, and family implications of a genetic disease. This process includes (1) interpretation of family and medical histories to assess the likelihood of disease occurrence and recurrence; (2) education about genetic phenomena, testing, management, prevention, resources, and research; and (3) counseling to promote informed choices and adaptation to risks and circumstances [13].

#### Risk communication and decision-making support

In genetic counseling for cancer, it is important to assess the risk of hereditary tumor syndromes and provide accurate and easy-to-understand information for patients and their families. Risk communication in genetic counseling helps patients and their families to understand complex genetic information, make informed choices about their health, and improve their quality of life.

When patients with hereditary tumor syndromes and their families face psychosocial issues, decision-making is an important aspect of helping them to cope with and move forward from these challenges. Shared decision-making is a collaborative process in which healthcare professionals and patients work together to make health decisions. It involves discussing the available options, benefits, and risks while considering the patient's values, preferences, and circumstances.

In view of the diverse and complex psychosocial issues faced by patients and their families, multiple specialists with appropriate expertise should be involved in providing decision-making support.

#### Psychosocial issues

The specific psychosocial issues faced by individuals undergoing genetic counseling for cancer include coping with the risk of cancer, practical, family, and children-related considerations, living with cancer, and emotions [20]. These issues should be identified as early as possible in genetic counseling, and patients and their families should be supported to cope with and adapt to them within the framework of a multidisciplinary team.

#### Approaches

Genetic counseling about hereditary tumor syndromes and genetic testing provides accurate medical information as well as opportunities for clients to discuss their concerns and anxieties and the support required for autonomous decision-making. A receptive attitude, non-directiveness, and empathy are essential in genetic counseling. Healthcare professionals should keep in mind that each client has their own individual preferences, sensitivities, prior knowledge, comprehension, level of anxiety, and trust in medical care.

All physicians should possess fundamental knowledge and skills in genetic counseling. Healthcare providers and institutions offering genetic testing should ensure the provision of appropriate genetic specialists for their clients as necessary. When genetic counseling services are unavailable at the primary hospital, clients should be referred to genetic specialists at an appropriate external facility [16].

#### Obtaining medical information from the client

In genetic counseling, medical information should be obtained from the client and their family for accurate risk assessment. The information gathered during genetic counseling includes medical history, family history, needs regarding genetic counseling, awareness of the disease, values, and psychosocial aspects.

#### Contents of information provided

Clients require a considerable amount of information about cancer and heredity in order for them to have an accurate understanding of hereditary colorectal cancer. The information provided includes an overview of hereditary tumors and genetic testing, and the social resources available. The psychosocial impact of a genetic test result should also be considered. The clinical significance of genetic testing should be explained in advance, so that the client can understand its advantages and disadvantages. The advantages are that it provides a definitive diagnosis, requires taking only a small amount of blood, and can be used for diagnosis regardless of whether or not there is a family history of cancer, for surveillance of other diseases that may develop, and for identification of biological relatives who may carry the same pathogenic variant. The disadvantages of genetic testing include certain diagnostic limitations, the possibility that it may not be able to etect or predict the onset of a specific disease, lack of insurance coverage, psychological effects, and potential social discrimination or stigma.

Genetic testing should be performed while considering the client’s burden from the medical, ethical, economic, and technical perspectives. The client must receive a written explanation of genetic testing, and informed consent should be obtained. Genetic counseling should be provided as needed, not only before and after genetic testing but also on an ongoing basis. It is also important to ascertain the client’s preferences in advance as to whether relatives should be informed about their genetic test results.

#### Timing of initial genetic counseling for HCRC

Genetic counseling should be started as early as possible in the following scenarios:

1. HCRC is suspected based on clinical information (e.g., age of onset, multiple or synchronous cancers, family history, histopathological findings)
2. MSI-High or dMMR is identified by companion diagnostics
3. A hereditary tumor syndrome is diagnosed or suspected by comprehensive genomic profiling (CGP). CGP refers to tests used in cancer genomic medicine, primarily targeting somatic genetic alterations in tumor tissues.

In addition to ongoing genetic counseling for the client with HCRC, genetic counseling for at-risk relatives is essential.

#### Genetic testing in minors

Genetic testing should generally be delayed until the individual reaches adulthood and can make autonomous decisions, especially in cases where the condition typically manifests in adulthood. If early diagnosis can lead to prevention or early treatment, genetic testing may be performed with parental consent and informed assent from the minor following an explanation appropriate to the child’s level of understanding [13].

#### Genetic counseling for patients with hereditary tumors

The role of genetic counseling extends beyond providing information related to genetic testing. It is also important to ensure that clients with a hereditary tumor syndrome receive comprehensive care, including surveillance, through multidisciplinary and cross-departmental collaboration, supporting the client’s autonomous decision-making, lifestyle adaptations, and psychosocial needs. To achieve this, it is necessary to establish a multidisciplinary team consisting of genetic specialists, physicians from various disciplines, nurses, clinical psychologists, medical social workers, and clinical laboratory technicians.

## Chapter II. Familial adenomatous polyposis

### Overview

- Familial adenomatous polyposis (FAP) is an autosomal dominant disorder characterized by the presence of colorectal adenomatous polyposis, which is caused primarily by a germline pathogenic variant (GPV) in the *APC* gene located on chromosome 5q22.2 (Side note II-1: *APC*-associated polyposis).
- Patients with FAP have an increased risk of developing colorectal cancer (CRC); therefore, timely and appropriate treatment interventions are crucial. FAP is also associated with extracolonic manifestations, necessitating surveillance and treatment considering these related conditions.

Side note II-1: *APC*-associated polyposis

Multiple causative genes and modes of inheritance are responsible for colorectal adenomatous polyposis, along with associated extracolonic manifestations. Therefore, surveillance, treatment, and familial management protocols have been developed based on specific causative genes. Consequently, there has been an increase in the number of disease names linked to these causative genes. For example, FAP and gastric adenocarcinoma and proximal polyposis of the stomach (GAPPS) are sometimes collectively referred to as *APC*-associated polyposis [9, 21], while diseases that result in adenomatous polyposis, including FAP, are occasionally grouped under the term “adenomatous polyposis syndrome” [9].

#### Clinical features

**Fig. 6:**
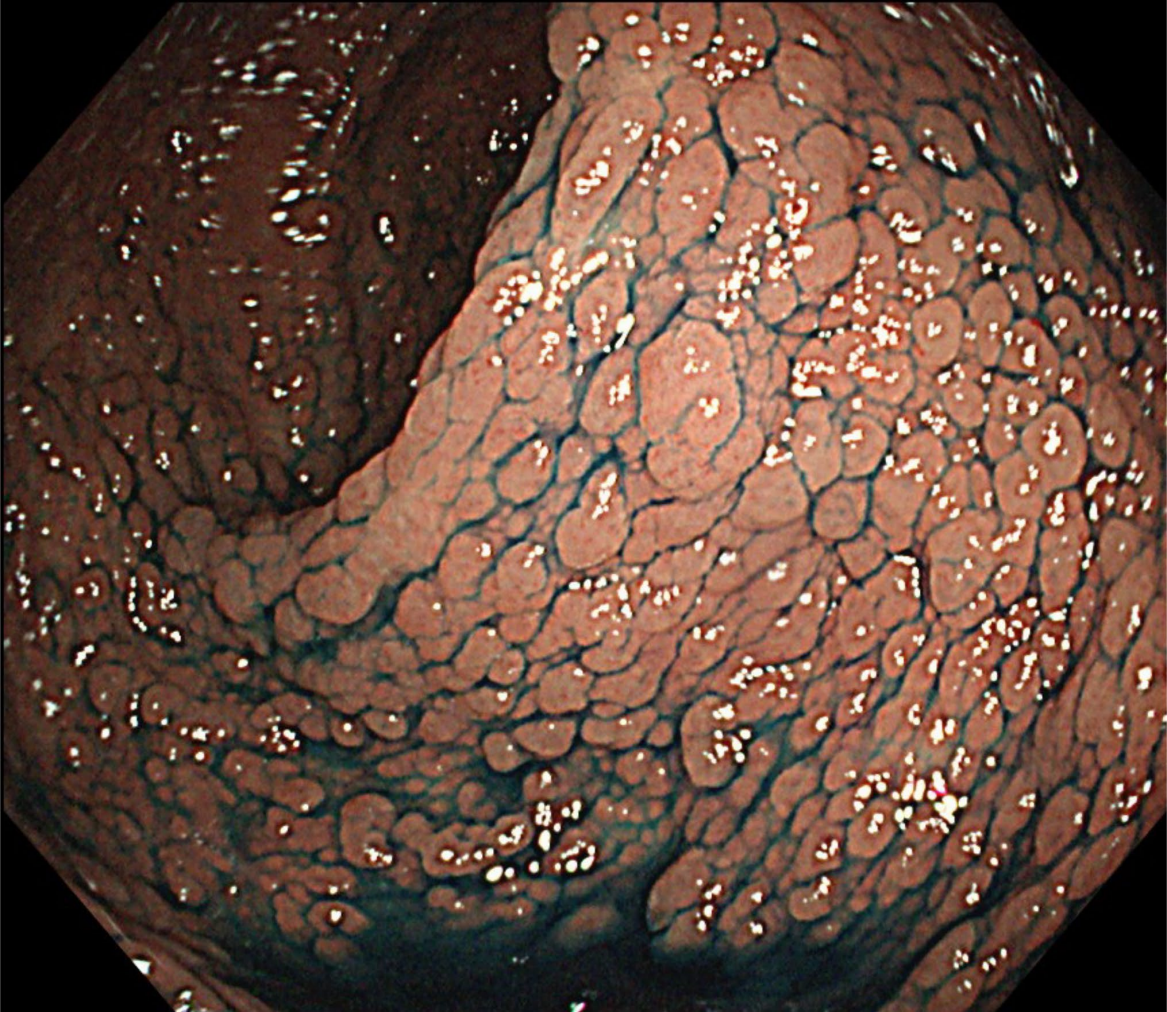
Severe/profuse/dense familial adenomatous polyposis

**Fig. 7:**
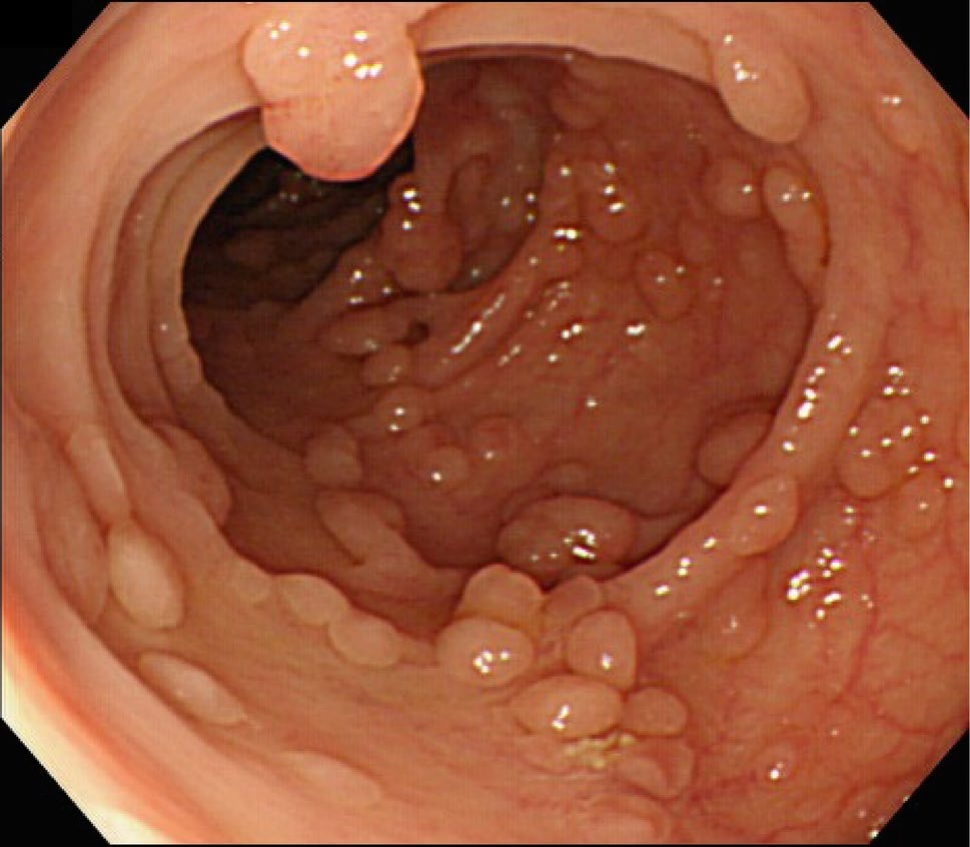
Sparse familial adenomatous polyposis

- FAP is a syndrome predisposing individuals to CRC, which includes both classical and attenuated forms.
- Classical FAP is characterized by the development of hundreds to thousands of colorectal adenomatous polyps, with onset typically at an average age of 16 years (range, 7–36). Ninety-five percent of individuals with classical FAP develop polyps by the age of 35 years, and CRC is inevitable without interventions such as proctocolectomy. The average age at diagnosis of CRC in untreated individuals is 39 years (range, 34–43). Classical FAP can be subcategorized based on adenoma density. Severe/profuse/dense FAP is characterized by > 1,000 adenomas covering the mucosa with no normal mucosa visible (Fig. 6). In contrast, sparse FAP involves approximately 100 to 1,000 adenomas against a background of normal mucosa (Fig. 7). However, adenoma density can vary according to the colorectal region, so the clinical significance of distinguishing strictly between severe/profuse/dense and sparse FAP is limited.
- Attenuated FAP (AFAP) is characterized by the presence of approximately 10–100 adenomas and is associated with GPVs in the *APC* gene. AFAP typically features fewer polyps, which are predominantly proximal, have a later age of onset (average 50–55 years), and have a lower lifetime risk of CRC (70%).
- Extracolonic manifestations of FAP include polyps in the stomach and duodenum, osteomas, dental abnormalities, congenital hypertrophy of the retinal pigment epithelium (CHRPE), benign skin lesions, desmoid tumors, adrenal masses, and other related cancers.
- GAPPS is characterized by proximal gastric polyposis and a high risk of gastric adenocarcinoma, with most reported cases showing no duodenal or colorectal lesions.

#### Causative gene and mode of inheritance

The causative gene for FAP is the *APC*, and it follows an autosomal dominant mode of inheritance.

The *APC* gene is located on the long arm of chromosome 5 (5q22.2) and is a tumor suppressor gene encoding a 311.8-kDa protein comprising 2,843 amino acid residues, with domains for binding β-catenin and Axin. APC plays a crucial role in suppressing formation of tumors by regulating degradation of β-catenin in the Wnt signaling pathway. β-catenin is phosphorylated by a complex that includes APC, Axin, and GSK3β when Wnt signaling is inhibited, leading to its ubiquitination and proteasomal degradation. However, when Wnt signaling is active, β-catenin is not degraded, accumulates in the cytoplasm, and translocates to the nucleus, where it activates the transcription of target genes via TCF. When a pathogenic variant occurs in the *APC* gene, the resulting APC protein is unable to bind β-catenin, even when Wnt signaling is active. This failure prevents degradation of β-catenin, leading to its accumulation within the cytosol, where it promotes the transcription of Wnt target genes, driving cell proliferation and ultimately leading to the development of adenoma [22, 23]. Patients with FAP carry a GPV in one allele of the *APC* gene in all their cells. Development of adenoma is triggered by acquisition of additional loss-of-function variants or deletions in the opposing *APC* allele, leading to the formation of numerous adenomas [24]. If left untreated, further genetic alterations occur in oncogenes like *KRAS* and tumor suppressor genes like *TP53*, with nearly all patients developing CRC by approximately 60 years of age.

Adenoma density is associated with the location of the germline variant in the *APC* gene and with the risk of development of CRC. In severe/profuse/dense FAP, germline variants often occur in *APC* codons 1250–1464 (particularly codon 1309) [25, 26]. In contrast, in AFAP, germline variants are frequently found in the 5′or 3′ ends of the *APC* gene or in regions subject to selective splicing (i.e., regions where specific exons are skipped during transcription because of the variant) [27]. Patients with severe/profuse/dense FAP generally develop adenoma and cancer at a younger age than those with other forms of FAP. In a multicenter study, the median age at which CRC developed was 41 years for severe/profuse/dense FAP, 48 years for sparse FAP, and 59 years for AFAP [28].

#### Associated tumors and extracolonic manifestations

FAP has both neoplastic and non-neoplastic extracolonic manifestations. Notably, extracolonic manifestations can be useful as auxiliary diagnostic criteria for FAP, regardless of the number of colorectal adenomas. Major manifestations associated with FAP include fundic gland polyposis, gastric and duodenal adenomas, periampullary adenomas, small bowel adenomas, desmoid tumors, skull osteomas, mandibular osteomas, epitheliomas, thyroid cancer, hepatoblastoma, adrenal tumors, and brain tumors.

#### Epidemiological features

- The prevalence of FAP in the general population is approximately 1:20,000 to 1:10,000 in Western countries and approximately 1:17,400 in Japan [29]. Patients with FAP account for < 1% of all CRC cases [30].
- CRC can occur in patients with FAP during their teenage years; however, approximately 50% of patients develop CRC in their 40 s, and nearly all will develop CRC by around 60 years of age if untreated [31].
- CRC remains the leading cause of death in patients with FAP. In the 1980s, approximately 80% of FAP-related deaths were attributable to CRC; however, this figure has decreased to approximately 60% since the 1990s [32].
- Among the major extracolonic manifestations, desmoid tumors and duodenal cancer are significant causes of death in patients with FAP, with respective frequencies of approximately 10% and 6% [32].

### Diagnosis

#### Diagnostic process

**Fig. 8:**
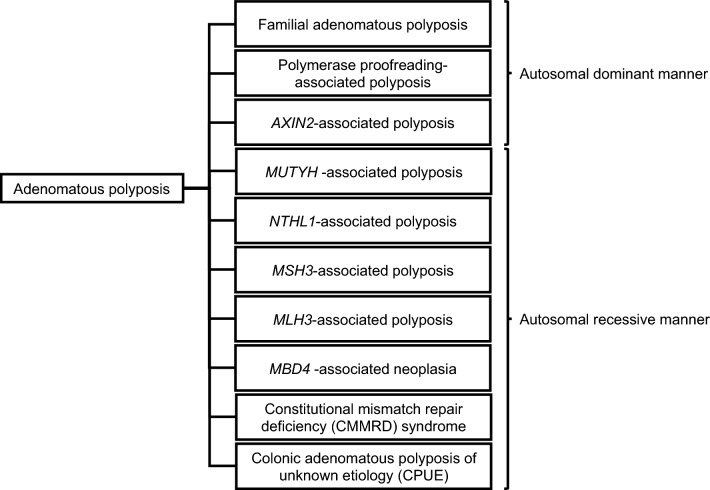
Classification of adenomatous polyposis according to causative gene

- FAP is diagnosed using the following three steps:
  o **Step 1: Risk assessment based on clinical information**
  o If > 10 polyps are identified during a colonoscopy, proceed to Step 2.
  o **Step 2: Pathological and molecular pathological evaluation**
  o If pathological evaluation confirms that the polyps are adenomatous, proceed to Step 3.
  o **STEP 3: Genetic testing**
  o A FAP diagnosis is confirmed if a GPV in the *APC* gene is detected. The classification of colorectal adenomatous polyposis diagnosed by genetic testing is shown in Fig. 8.
- No GPV in the *APC* gene is identified in 20%–40% of patients with colorectal adenomatous polyposis [33–35]. Possible reasons include somatic *APC* mosaicism, colorectal adenomatous polyposis due to non-*APC* causative genes, colorectal adenomatous polyposis of unknown etiology, and limitations in analytical techniques.
- Extracolonic manifestations are useful as auxiliary diagnostic criteria for FAP, regardless of the number of colorectal adenomas.

#### Diseases/conditions requiring differential diagnosis

##### Somatic *APC* mosaicism

Somatic *APC* variants occurring during embryonic development can result in a mosaic state in which some cells have the *APC* variant and others do not. Colorectal adenomatous polyposis resembling FAP can develop if the pathogenic variant in the *APC* gene occurs in cells that differentiate into colonic mucosal cells. The degree of mosaicism depends on the timing of the somatic *APC* variant. For example, adenomatous polyposis will be present in the left colon and rectum but not in the right colon if the variant arises only in cells that contribute to the hindgut. Adenomatous polyposis can be inherited if the pathogenic variant occurs before differentiation of the mesoderm into the gonads but not if the variant occurs after the three germ layers have differentiated, when only the endoderm (which differentiates into structures like the gastrointestinal tract) would be affected. Somatic *APC* mosaicism has been reported in 1.6%–4% of patients with FAP and identified *APC* variants and is thought to account for 11%–20% of cases of FAP without a family history [36, 37]. Recent advances in next-generation sequencing have made it possible to detect low-frequency GPVs that are undetectable by conventional Sanger sequencing. Low-frequency pathogenic variants have been identified in 25–50% of patients clinically suspected of having FAP or thought to have colorectal polyposis of unknown etiology, leading to a diagnosis of somatic *APC* mosaicism [38, 39]. In a multicenter study in Japan, somatic *APC* mosaicism was identified in 9 (7.3%) of 123 cases of colorectal adenomatous polyposis [35]. Low-frequency pathogenic variants were identified in 3 of these patients, and the remaining 6 were diagnosed based on the presence of the same pathogenic variant in multiple tumor tissues or in tumor tissue and adjacent normal mucosa.

##### Gastric adenocarcinoma and proximal polyposis of the stomach (GAPPS)

- GAPPS is an autosomal dominant disorder that is primarily characterized by fundic gland polyposis, caused by GPVs in the promoter 1B region of the *APC* gene, and characterized as *APC*-associated polyposis [9, 21, 40].
- In the stomach, the *APC* promoter 1A region is inactivated by hypermethylation, whereas the promoter 1B region regulates gene expression. A pathogenic variant in the promoter 1B region results in the loss of normal expression of APC protein in the stomach. In contrast, expression of APC protein in the colon/rectum is primarily regulated by the promoter 1A region; therefore, a pathogenic variant in the promoter 1B region does not affect expression of APC protein in the colon/rectum. Thus, GAPPS is characterized by fundic gland polyposis with minimal colorectal adenomas [21, 40].
- Patients with GAPPS have a 12%–25% lifetime risk of developing gastric cancer [41]; however, their risk of CRC is unknown, and risk management should be based on family history. Surveillance for gastric cancer should begin at the age of 15 years, and prophylactic total gastrectomy may be considered after the age of 20–30 years [42]. Total colonoscopy must be considered to exclude colorectal polyposis.

##### *MUTYH*-associated polyposis (MAP)

- MAP is an autosomal recessive disorder caused by biallelic pathogenic variants in the *MUTYH* gene, which is involved in base excision repair [43]. Patients with MAP typically develop 10–100 colorectal adenomas; however, over 100 lesions can be found [44]. Multiple serrated polyps may also be observed, and 18% of patients with MAP meet the diagnostic criteria for serrated polyposis syndrome [9]. The penetrance of CRC in MAP by the age of 60 years ranges from 43 to 100% [45], and surveillance with total colonoscopy should begin at 25–30 years of age. Patients with MAP also have extracolonic manifestations similar to those observed in FAP, with lifetime risks of duodenal polyposis of 17%–34%, duodenal cancer of 4%–5%, and fundic gland polyps of 11%, necessitating esophagogastroduodenoscopy (EGD) as early as 30–35 years of age. Treatment follows the same principles as those for AFAP [44]. This disease is rarely reported in Japan, and the frequency of *MUTYH* variants and proportion of CRC cases attributed to MAP in the Japanese population are unknown. However, a MGPT study that included 123 patients with colorectal adenomatous polyposis identified 2 patients with MAP (1.6%), both of whom had > 100 adenomas [35, 46].
- Recently, germline monoallelic pathogenic variants in *MUTYH* have been detected by comprehensive genome profiling tests. Individuals with a family history of CRC and a monoallelic *MUTYH* pathogenic variant have an increased risk of CRC [47, 48]; however, meta-analyses and recent large-scale studies have reported that the risk of CRC is not increased in monoallelic *MUTYH* carriers [49, 50]. In view of these conflicting findings, the National Comprehensive Cancer Network guidelines in Western countries recommend that asymptomatic monoallelic *MUTYH* carriers with a family history of CRC in a first-degree relative should consider surveillance with colonoscopy every 5 years, starting at the age of 40 years or 10 years younger than the age at which CRC was diagnosed in the first-degree relative. This recommendation aligns with the standard surveillance protocol for individuals without genetic information but with a family history of CRC in a first-degree relative. Specific recommendations cannot be made for individuals with a family history of CRC in a second-degree relative because of insufficient data [9].

##### Polymerase proofreading-associated polyposis (PPAP)

PPAP is an autosomal dominant disorder caused by GPVs in the exonuclease domain of the *POLE* or *POLD1* gene, which are involved in proofreading during DNA replication [51, 52]. PPAP accounts for 0.1%–0.4% of familial tumors and 0.3%–0.7% of CRCs and cases of colorectal polyposis [53]. Patients often present with dozens of colorectal adenomas; however, cases without adenomas have also been reported, and the condition is associated with an increased risk of CRC [52, 54–57]. While CRC is the most common tumor associated with PPAP, extracolonic manifestations vary depending on the causative gene. *POLD1*- related cases are linked to endometrial cancer, breast cancer, and brain tumors, whereas *POLE*- related cases are associated with duodenal adenomas and cancer, ovarian cancer, brain tumors, pancreatic cancer, breast cancer, and melanoma [53, 54, 56–63]. Somatic *POLE* variants have been observed in 2%–8% of CRCs and 7%–15% of endometrial cancers; however, somatic *POLD1* variants are extremely rare. Cancers caused by these somatic variants are characterized by a microsatellite-stable, ultra-mutated tumor phenotype with distinct mutational signatures [53]. Recently, a Japanese family with a *POLD1* variant was identified in which the proband had attenuated colorectal polyposis and endometrial cancer [64].

##### MSH3-associated polyposis

*MSH3* is a DNA mismatch repair gene that contributes to colorectal carcinogenesis via microsatellite instability. Autosomal recessive colorectal polyposis is associated with biallelic GPVs in the *MSH3* gene; however, whether monoallelic *MSH3* variants increase the risk of CRC cannot be determined owing to insufficient evidence [65–67]. Surveillance for asymptomatic biallelic *MSH3* carriers should begin with colonoscopy at the age of 25–30 years, with repeated examinations every 2–3 years if no polyps are found. The interval should be shortened to 1–2 years if polyps are detected, and surgery should be considered if endoscopic management becomes challenging [9].

##### *MLH3*-associated polyposis

*MLH3* is another DNA mismatch repair gene, and autosomal recessive colorectal polyposis is linked to biallelic GPVs (e.g., p.S1188X) in *MLH3*. In an initial report involving 4 families and 5 cases, patients were diagnosed at a relatively older age (48–52 years), with 1–200 colorectal adenomatous polyps, and some had developed malignancies such as CRC and breast cancer [68]. Another variant (p.N121Mfs*49) causes male infertility without associated adenomatous polyposis [69, 70].

##### NTHL1-associated polyposis

*NTHL1* is involved in base excision repair and acts on oxidized pyrimidine bases [71]. Biallelic pathogenic variants of *NTHL1* are associated with colorectal polyposis [72–74] and with breast and endometrial cancers [71, 72]. Lifetime risks for CRC and extracolonic cancers are 64% and 86%, respectively, for men and 47% and 100% for women [75]. Monoallelic pathogenic variants of *NTHL1* are not associated with colorectal polyposis or CRC [76]. Colonoscopy surveillance for asymptomatic biallelic *NTHL1* carriers should begin at the age of 25–30 years, with repeated examinations every 2–3 years if no polyps are found. The interval should be shortened to 1–2 years if polyps are detected, and surgery should be considered if endoscopic management becomes difficult. Surveillance for breast cancer should begin with annual mammography with tomosynthesis or contrast-enhanced magnetic resonance imaging (MRI) at the age of 40 years. However, there is insufficient evidence to recommend risk-reducing mastectomy. For endometrial cancer, early diagnosis based on awareness of abnormal uterine bleeding and endometrial cytology is crucial [9].

##### *MBD4*-associated polyposis (*MBD4*-associated neoplasia syndrome)

Biallelic GPVs in *MBD4*, a base excision repair gene, cause colorectal adenomatous polyposis [77]. Affected patients and their relatives develop various tumors, including up to hundreds of colorectal adenomatous polyps, CRC, acute myeloid leukemia, uveal melanoma, granulosa cell tumors of the ovary, and meningioma. A Japanese case report described a patient with 30 adenomatous polyps who harbored a monoallelic GPV in *MBD4* [78].

##### *AXIN2*-associated polyposis

*AXIN2* plays an important role in the Wnt signaling pathway, where it regulates the stability of β-catenin, and is essential for development of the teeth. Families with GPVs in *AXIN2* have oligodontia (congenital absence of multiple teeth) and multiple colorectal adenomatous polyps, and develop CRC [79]. Serrated lesions have also been reported in some cases [80]. Extracolonic manifestations include upper gastrointestinal polyps, breast cancer, lung cancer, hepatocellular carcinoma, and prostate cancer [81].

##### Constitutional mismatch repair deficiency (CMMRD) syndrome

CMMRD syndrome is an autosomal recessive disorder caused by biallelic GPVs in mismatch repair genes and is associated with colorectal adenomatous polyposis. Affected individuals develop hematologic malignancies, brain tumors, and CRC during childhood.

##### Colonic adenomatous polyposis of unknown etiology (CPUE)

If genetic testing using MGPT does not identify a causative gene, the condition is termed CPUE [9]. However, conventional genetic testing may not detect somatic mosaicism [35, 37], gene inversions [82], translocations [83], intronic variants distant from exon–intron junctions [84, 85], or promoter region variants [86]. As a result, some cases of CPUE may be attributed to technical limitations in identifying the causative genes. If ≥ 100 adenomas are identified in the colon/rectum, surveillance, treatment, and familial management should be implemented as for FAP, even if genetic testing has not been performed or no pathogenic variants are detected.

### Surveillance and treatment

#### Colorectal adenomas and carcinomas

##### Characteristics and classification

- FAP can be classified as dense FAP, sparse FAP, or AFAP based on adenoma density. Dense and sparse FAP together are sometimes referred to as classical FAP.
- The density of adenomas is associated with the location of the GPV in the *APC* gene and the risk of development of CRC.
- InSiGHT has proposed a staging system based on endoscopic evaluation using the number and size of adenomas (i.e., the polyp burden). The polyp burden correlates strongly with the interval between surveillance colonoscopies and the indication for surgery [87]. A marked increase in polyp burden over time is used as a clinical criterion for determining the need for surgery [88].

##### Surveillance and cancer prevention

*Surveillance*

- Surveillance by total colonoscopy for FAP should start at around 10 years of age, with intervals of 1–2 years for classical FAP and 2–3 years for AFAP starting at 18–20 years. The age at which to start surveillance by total colonoscopy should be considered for both genetically diagnosed cases of FAP and those without genetic testing.
- A European study that investigated the frequency of CRC in patients with FAP who were younger than 20 years found no cases of CRC before the age of 10 years and an incidence rate of 0.2% in the 11–15-year age group [30]. Therefore, the recommended age for initiating surveillance by total colonoscopy in patients with FAP is approximately 10 years [22]. However, patients with dense FAP can develop CRC before the age of 10 years [89], so require careful attention.
- The onset of CRC occurs 10–15 years later in AFAP than in classical FAP [90], with cases of CRC before the age of 30 years being rare [91]. Therefore, surveillance by total colonoscopy should begin in the late teens (18–20 years) [21].
- If no colorectal adenomas are detected by total colonoscopy, the interval between examinations can be extended in cases where genetic testing has not been performed. Further extension of the interval can be considered based on clinical judgment if multiple consecutive surveillance colonoscopies do not show any adenomas [9].

*Chemoprevention*

During surveillance, Nonsteroidal anti-inflammatory drugs (NSAIDs) have been used during surveillance to suppress development and growth of adenomatous polyps. However, their effectiveness in preventing cancer and the effects in long-term use are unclear.

##### Treatment

*Endoscopic treatment*

The most reliable treatment for prevention of death from CRC is prophylactic colectomy before the development of cancer. However, technical advances have made it possible to resect many colorectal polyps safely during colonoscopy. A single-center trial investigated the outcomes in patients with sparse FAP who declined surgery and were managed by regular endoscopic polypectomy and follow-up [92]. No cases of perforation or severe bleeding were observed during colonoscopy, and no cases of advanced cancer were detected during follow-up. A subsequent multicenter, prospective, exploratory Phase I/II study (J-FAPP III) evaluated the safety and efficacy of intensive downstaging polypectomy [93]. Colonoscopies were performed until all adenomas ≥ 10 mm were resected, followed by removal of adenomas ≥ 5 mm and, if possible, those that were smaller. Of 166 patients who had not undergone colectomy, 150 (90.4%) completed the 5-year intervention period without surgery.

*Surgical treatment*

**Fig. 9:**
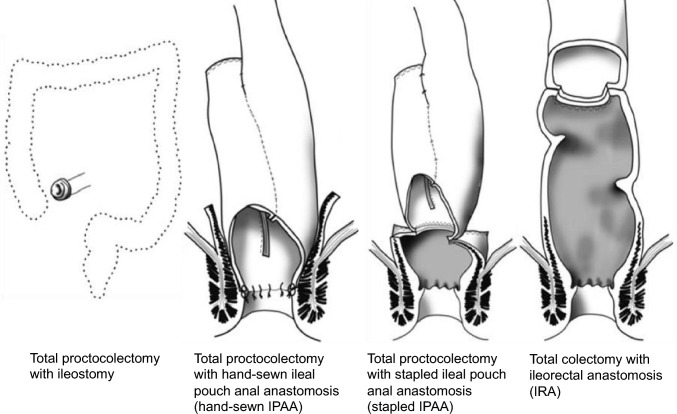
Surgical procedures for patients with familial adenomatous polyposis

- The most reliable treatment for prevention of death from CRC is prophylactic colectomy before cancer onset.
- The primary surgical procedures are total proctocolectomy with permanent ileostomy, total proctocolectomy with ileal pouch–anal anastomosis (IPAA), and total colectomy with ileorectal anastomosis (IRA) (Fig. 9 and Table 8).
- The timing of prophylactic colectomy should be decided based on following considerations, (1) the cumulative prevalence of CRC [32], (2) adenoma density [94], (3) adenoma size and morphology, (4) age at death and cancer onset in at-risk relatives and the presence of desmoid tumors [95], (5) location of *APC* gene variants [96, 97], (6) the patient’s educational and occupational environment [98], (7) fertility post-IPAA [99] and male sexual dysfunction [100], (8) gastrointestinal symptoms such as diarrhea, abdominal pain, and rectal bleeding, and (9) pathological findings in the tumors. Considering the prevalence of CRC, patients with classical FAP should undergo surgery as early as their late teens, with the most common age for surgery being in the 20 s [101, 102]. A Japanese study in patients who had FAP with known locations of pathogenic *APC* variants reported a rapid increase in the risk of developing stage II or higher CRC after the age of 34 years in patients with the classical FAP genotype and after the age of 49 years in those with the AFAP genotype [97]. Identifying the location of pathogenic variants by genetic testing can help to determine the timing of prophylactic colectomy.
- Total proctocolectomy with IPAA is currently the standard procedure and accounts for an increasing proportion of the procedures performed [103–106]. Use of laparoscopic surgery has also been increasing [105–110]. Considering the balance between benefits and risks, the decision to perform a temporary ileostomy should be made on an individual basis in cases of IPAA.
- If a desmoid tumor is identified in the mesentery, the indication for IPAA should be assessed carefully because of the risk of recurrence or growth of the tumor and the technical challenges involved. Notably, laparoscopic surgery, particularly for IRA, is associated with a lower postoperative incidence of desmoid tumors [111, 112].
- Total colectomy in women with FAP may reduce fertility (Side Note II-2: Surgery, fertility, pregnancy, and childbirth).
- The surgical approach for a patient with FAP who has developed CRC should be determined based on the stage and location of the CRC as well as other clinical considerations. If curative resection is possible, total proctocolectomy or total colectomy with regional lymph node dissection should be considered. Managing unresectable advanced CRC, metastatic lesions, and chemotherapy should follow the same protocols as those for sporadic CRC.

**Table 8:** Characteristics of the surgical procedure for FAP

Surgical procedures	Total proctocolectomy with permanent ileostomy	Total proctocolectomy with ileal-pouch anal anastomosis (IPAA)	Total colectomy with ileorectal anastomosis (IRA)
Advantages	Colorectal cancer completely prevented	Colorectal cancer almost prevented Preservation of natural anal function	Good bowel function Relatively easy surgical technique Lower complication rate than that of IPAA
Disadvantages	Deteriorated body image with permanent colostomy	A complex surgical technique Unstable bowel function Possibility of cancer development at the remaining rectal mucosa near the anus Possibility of pouchitis	Possibility of rectal cancer development (depending on the number of adenomas, location of the germline variant in the *APC* gene, or length of the residual rectum)

Side Note II-2: Surgery, fertility, pregnancy, and childbirth.

- A study of 58 women with FAP in Denmark [113] reported a fertility rate of 90%, which was comparable to that in the general population. Another study involving 162 European women with FAP found that the fertility rate in patients with FAP who had not undergone surgery was comparable to that in the general population, as was that in patients who underwent IRA; however, those who underwent IPAA had a 0.46 times lower fertility rate [30]. In contrast, a study of 138 patients with FAP in the Netherlands reported that fertility was unrelated to the type of surgery but was associated with age at the time of initial surgery [114].
- Reduced fertility after IPAA may be a result of postoperative adhesions. In one study, hysterosalpingography performed after total colectomy showed adhesions of the fallopian tubes to the pelvic wall in 48% of cases, unilateral occlusion in 43%, and bilateral occlusion in 10% [115].
- The fertility rate has been reported to be significantly higher after laparoscopic surgery than after open surgery in patients with FAP and ulcerative colitis [116]. Research specifically involving patients with FAP also found that laparoscopic surgery was associated with a lower rate of postoperative bowel obstruction and a smaller reduction in female fertility [107]. However, no prospective studies have been conducted specifically in patients with FAP. Furthermore, vaginal delivery after IPAA is reportedly safe [117, 118]; however, the risk of damage to the anal sphincter and pudendal nerve injury after episiotomy should be considered in vaginal delivery post-IPAA.

##### Surveillance of the lower gastrointestinal tract after colorectal surgery

After IRA, long-term surveillance, including endoscopy, is required to monitor for development of cancer in the remaining rectum (Side Note II-3: Risk of rectal cancer post-total colectomy with IRA). After stapled IPAA, 2–3 cm of rectal mucosa typically remains, and a small amount of rectal mucosa may remain even after hand-sewn IPAA. Therefore, long-term postoperative surveillance of the remaining rectal mucosa is necessary, whether stapled or hand-sewn. The reported incidence of adenoma in the ileal pouch after IPAA ranges from 6.7% to 74% [119–122]. Development of cancer has also been reported, necessitating long-term endoscopic surveillance [123, 124]. Pouchitis after IPAA for FAP occurs in approximately 5% of patients, which is lower than the rate observed after IPAA for ulcerative colitis [125]. Clinical symptoms include fever, diarrhea, and anemia, and endoscopic surveillance should be performed promptly if such symptoms appear. Surveillance for recurrence should be performed as for sporadic CRC where curative resection is performed for concomitant CRC.

Side Note II-3: Risk of rectal cancer post-total colectomy with IRA

Long-term follow-up after IRA shows that rectal cancer occurs in 24%–43% of cases [126, 127]. In one study, rectal resection was necessary during up to 20 years of follow-up post-IRA in 10% of patients with AFAP, 39% of those with sparse FAP, and 61% of those with dense FAP [128]. With advances in surgical techniques and the increased frequency of IPAA [103, 104], the rate of rectal resection after IRA has decreased from 40 to 13%, with a decline in the cumulative incidence of residual rectal cancer post-IRA [107, 129, 130].

#### Fundic gland polyposis and gastric adenomas

##### Characteristics and classification

**Fig. 10:**
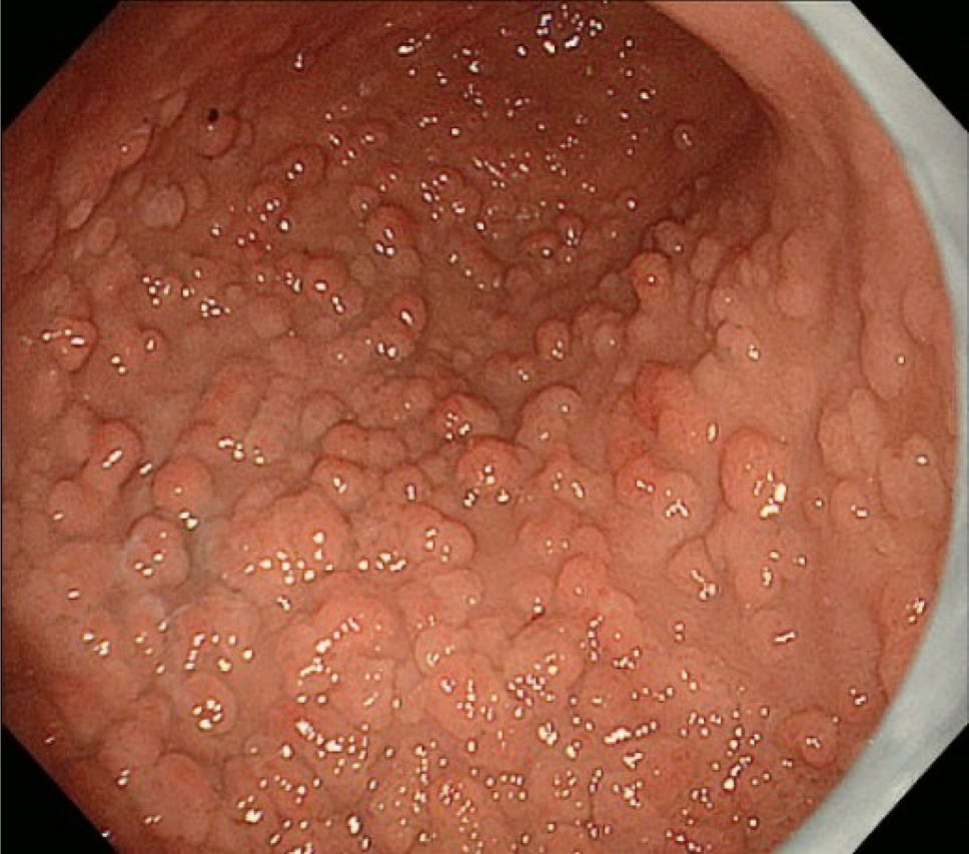
Fundic gland polyposis

**Fig. 11:**
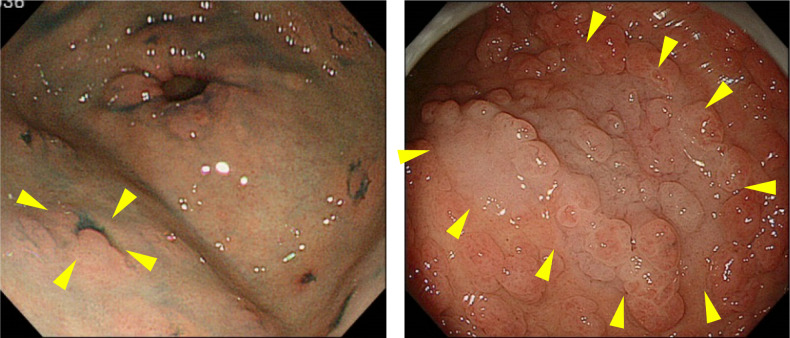
Gastric adenoma (left, depressive type; right, elevated type)

- Fundic gland polyposis (Fig. 10) is distinct from fundic gland polyps observed in the stomachs of individuals without FAP in terms of number and size. Fundic gland polyposis has been observed in 88% of patients with FAP [131], making it useful for auxiliary diagnosis.
- Fundic gland polyposis occurs more frequently in patients with FAP who are not infected with *Helicobacter pylori* [131].
- Adenomas (Fig. 11) and cancers can develop within fundic gland polyposis.
- The relationship between fundic gland polyposis, gastric adenomas, and the *APC* genotype is unclear [132].
- In patients with FAP, neoplastic lesions in the stomach are classified based on morphology, and location [133].

##### Surveillance

- EGD surveillance is necessary because neoplastic lesions such as adenomas and cancers can develop within fundic gland polyposis.
- Low-grade dysplastic lesions occur frequently, but high-grade dysplastic lesions are less common [131, 134]. There have also been reports of gastric cancer, albeit less frequently [135].
- The timing and frequency of EGD surveillance should generally follow the degree of duodenal lesions (staged using the Spigelman classification), which occur frequently.
- Neoplastic lesions are more common in Asian patients with fundic gland polyposis than in their Western counterparts [131, 134, 136–138]. Given that some of these lesions can be difficult to diagnose, EGD surveillance at intervals of 1–2 years may be considered [136]. Particularly careful EGD surveillance is required in patients with diffuse (carpet-like) polyposis and lesions with significant elevation.

##### Treatment

- Active treatment for fundic gland polyposis itself is generally not necessary.
- Although no established consensus exists regarding the endoscopic treatment of gastric adenomas, treating all low-grade adenomas is generally unnecessary. Typically, treatment is considered for tumorous lesions ≥ 10 mm or high-grade adenomas [9].

#### Duodenal adenomas and carcinomas

##### Characteristics and classification

**Fig. 12:**
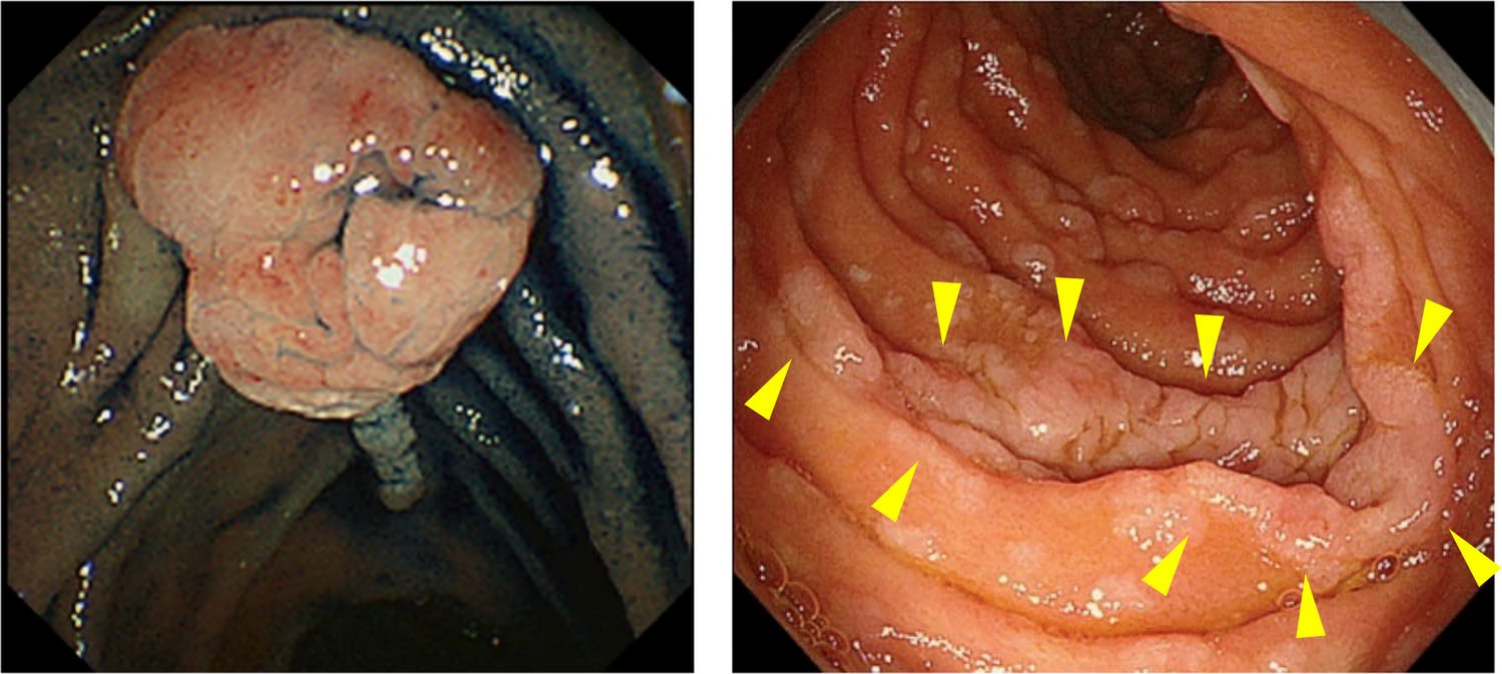
Ampullary adenoma (left) and multiple duodenal adenomas (right)

**Fig. 13:**
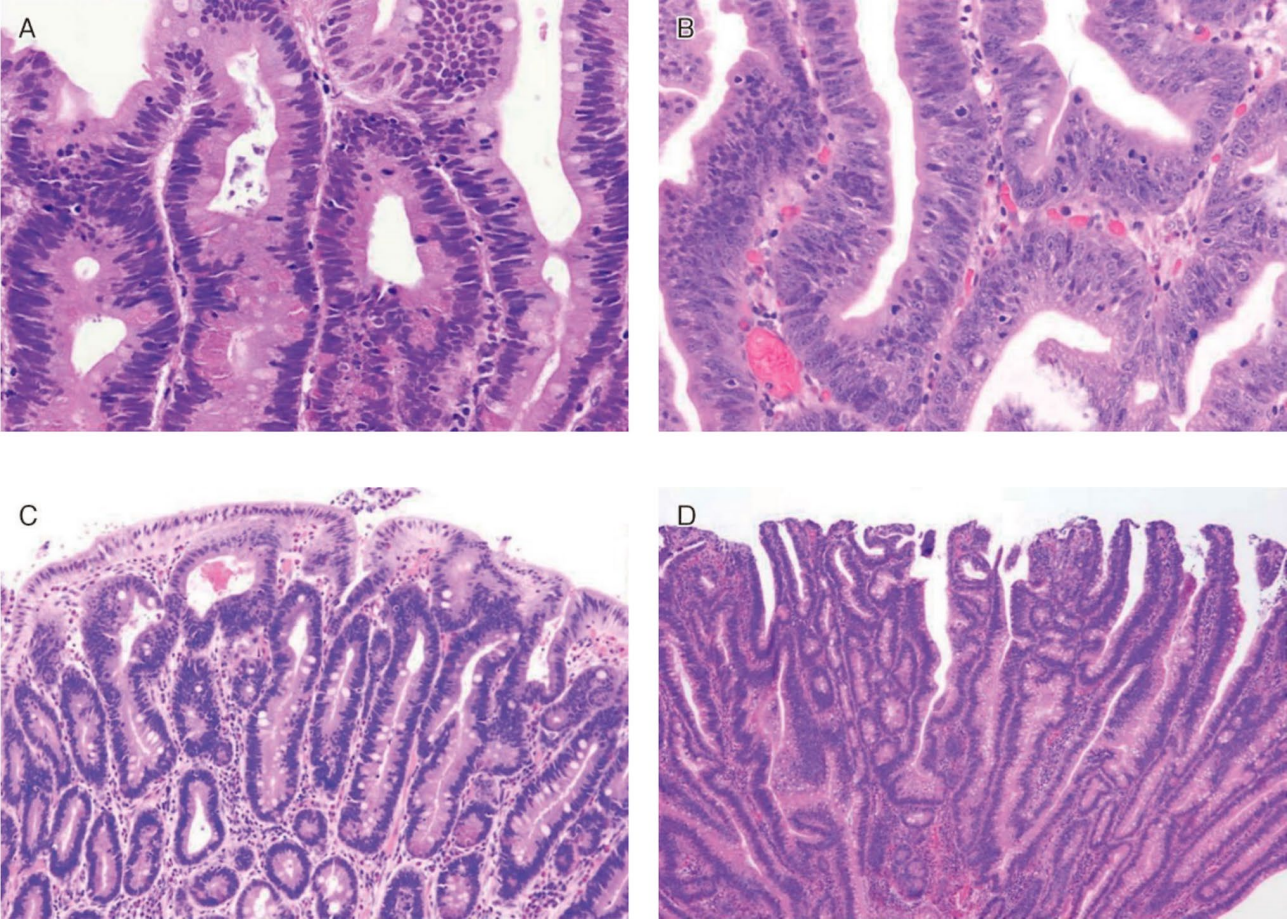
Histology of familial adenomatous polyposis-associated duodenal adenomas. **A** Low- grade adenoma: the tumor glands are rather uniform and the adenomatous epithelial cells show basally oriented, elongated nuclei. **B** Intramucosal carcinoma: tumor glands show significant irregularity, nuclear stratification, and occasional prominent nucleoli. Note that high-grade dysplasia in the Spigelman classification includes non-invasive intramucosal carcinoma in the Japanese classification. **C** Tubular adenoma: this lesion shows a relatively regular tubular architecture. **D** Tubulo-villous adenoma: this lesion partially exhibits villous architecture, composed of fibrovascular cores lined by dysplastic epithelium

- Duodenal and ampullary adenomas (Fig. 12) are rarely observed in healthy individuals without FAP, and the presence of multiple duodenal adenomas is a hallmark of FAP, making it useful for auxiliary diagnosis.
- Duodenal adenomas are observed in 30%–90% of patients with FAP [139–141], with an increase in prevalence after the age of 40 years [140, 141].
- According to a multicenter study in Japan, the cumulative incidence of duodenal adenoma was 39.2% by the age of 50 years, with classical FAP having a substantially higher cumulative incidence than AFAP (42.5% vs. 23.5%) [142].
- Ampullary tumors have been observed in up to 72% of cases [143].
- Forward-viewing and side-viewing endoscopy is used to observe duodenal lesions and the ampullary region.
- The relationship between duodenal adenomas, ampullary tumors, and the *APC* genotype is not clearly established [132].
- The Spigelman classification has reproducibility between and within examiners [144].
- Duodenal cancer accounts for approximately 3% of deaths in patients with FAP and is one of the leading causes of death after prophylactic colectomy [32, 145].
- The relative risk of duodenal cancer is approximately 250–330.8 times higher in patients with FAP than in the general population [146, 147], with an estimated cumulative incidence rate of 4–10% [30, 148, 149].
- The Spigelman scoring system is used for clinicopathological classification of duodenal adenomas in patients with FAP [137].
- The Spigelman classification is determined based on the number and maximum diameter of duodenal adenomas seen on endoscopic examination and assesses the structure of the adenoma (Fig. 13) for histological type and dysplasia. Minor modifications have been made to the original classification system [150].
- Use of narrow-band imaging increases the detection rate of duodenal adenomas but does not affect the Spigelman classification [151].

##### Surveillance

- Although duodenal adenomas grow slowly [140, 152, 153], regular endoscopic surveillance is necessary because of the risk of progression and malignant transformation.
- Generally, surveillance should begin at around the age of 20–25 years, with surveillance intervals of 4–5 years for Spigelman stage 0, 2–5 years for stage I, 2–3 years for stage II, and every 6 months to 2 years for stage III [141]. However, endoscopic examinations at intervals of once every 1–3 years may also be considered, given the possibility of missed lesions and occurrence of fundic gland polyposis or gastric adenomas.
- Biopsies from the ampulla are safe with a low risk of pancreatitis and can elevate the Spigelman stage in the absence of visible abnormalities [154]. However, routine biopsies from ampullae without visible abnormalities are usually not necessary because ampullary tumors generally progress slowly [148].

##### Treatment

**Fig. 14:**
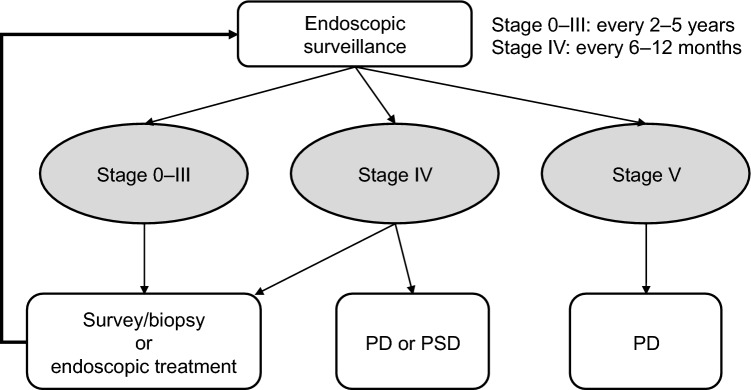
Surveillance of duodenal adenoma based on revised Spigelman’s classification. PD, pancreaticoduodenectomy; PSD, pancreas sparing duodenectomy

- Guidelines for management of duodenal adenomas according to the modified Spigelman classification are shown in Fig. 14
- Endoscopic treatments for duodenal adenoma include snare resection, argon plasma coagulation, and other techniques [143]. Recent reports on the safety and efficacy of new treatment techniques (such as bipolar snare resection, cold snare polypectomy, and underwater endoscopic mucosal resection) have expanded the treatment options [155–158].
- Complete endoscopic resection of Spigelman stage II/III duodenal adenoma is associated with high recurrence and complication rates (50%–100%) [141]. However, recent improvements in endoscopic resection techniques and the introduction of new treatments have increased the safety and efficacy of endoscopic resection [155–159].
- A systematic review identified modified Spigelman stage IV as a risk factor for the development of non-ampullary duodenal carcinoma [160]. The incidence of duodenal cancer was approximately 2.3% for stage II, 2.4% for stage III [161], and 7%–36% for stage IV [148, 161]. Therefore, evaluation by a specialist for surgical intervention or surveillance every 6–12 months is recommended. Although downgrading by endoscopic treatment has recently been described [138, 143, 162, 163], whether this significantly alters the natural history of the disease is still not known.
- The standard surgical procedure for patients with concomitant carcinoma (Spigelman stage V) is pancreaticoduodenectomy. However, less invasive surgeries, such as pancreas-preserving duodenectomy, which does not hinder postoperative endoscopic surveillance and has a stronger prophylactic intent, are selected for stage IV cases [164–166].
- Treatment for ampullary tumors should be considered when maximum diameters are ≥ 1 cm or showing advanced histology (such as tubulovillous adenoma or high-grade dysplasia), or if symptoms of obstruction (such as liver dysfunction or pancreatitis) are present. Endoscopic ampullectomy by experts should be considered first [167].
- Endoscopic ampullectomy has been reported to have an adverse event rate of approximately 10%, a residual lesion rate of 28%, and a recurrence rate of 17%, necessitating careful case selection and meticulous surveillance [167].

#### Desmoid tumors

##### Characteristics and classification

**Fig. 15:**
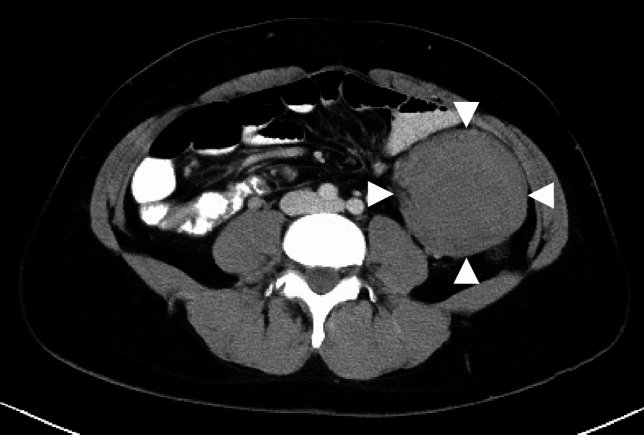
Intra-abdominal desmoid tumor

- Desmoid tumors (Fig. 15) are observed in 8%–20% of patients with FAP [95, 168–170]. These tumors are a type of non-metastasizing fibroma but grow invasively.
- Desmoid tumors are classified as intra-abdominal, extra-abdominal, or mixed, with the intra-abdominal type accounting for 70% of all desmoid tumors [101]. These tumors often develop in the abdominal wall, mesentery, or retroperitoneum within 2–3 years after total proctocolectomy [118, 171, 172].
- In Japan, the incidence of desmoid tumors is 10%–15%, with intra-abdominal desmoids accounting for 71.8%–80% of all these tumors. They are considerably more common in individuals under 30 years of age and in females, with approximately two-thirds of cases occurring within 1 year within 1 year after total proctocolectomy [172, 173].
- Desmoid tumors associated with FAP differ from sporadic desmoid tumors in terms of mechanisms of development and clinical characteristics, including their location, making them useful for auxiliary diagnosis of FAP. "Usually, FAP-related desmoid tumors develop due to somatic variants in the opposite allele of *APC*, resulting in functional impairment of both *APC* alleles (two-hit theory). In clinical practice, compared to sporadic desmoid tumors, FAP-related desmoid tumors tend to be larger, more commonly multiple, more often intra-abdominal, and have an earlier age of onset.”
- Desmoid tumors may spontaneously regress or stabilize over time [174–176].
- When desmoid tumors develop intra-abdominally (including in the retroperitoneum), they can cause complications such as bowel obstruction, perforation, abscess formation, and ureteral obstruction, making treatment challenging.
- Data from a multicenter study in Japan found no difference in the risk of development of desmoid tumors between laparoscopic surgery and open surgery [105, 106].
- Recent reports, including data from Japan, suggest that the risk of development of desmoid tumors is higher after IPAA than after IRA [172, 177].
- The mortality rate for desmoid tumors is in the range of 0% to 14% [119, 168, 170, 174].
- There is a correlation between the development of desmoid tumors and pathogenic *APC* variants, particularly on the 3′side of codon 1444 or within codons 1445–1580 [178, 179]. Another report showed a high incidence of desmoid tumors in cases with pathogenic variants on the 3′ side of codon 1399, noting severe and potentially lethal symptoms [180]. There is also a correlation between desmoid tumors and 3′ side pathogenic variants of *APC* in patients with AFAP [9, 24].
- The staging system for intra-abdominal desmoid tumors developed by Church et al. [181] requires evaluation of the trend in growth, necessitating prospective assessment over 6 months. Retrospective evaluations may lack accurate data on growth trends.
- For desmoid tumors associated with FAP that occur outside the abdominal cavity (mostly in the abdominal wall), a “wait and see” approach or surgical resection is generally selected, with a good prognosis. Intra-abdominal desmoid tumors are typically managed noninvasively, but tumor growth or progression leading to organ dysfunction (urinary tract, bowel) may result in a decline in quality of life or a poor prognosis. Therefore, a new classification has been proposed in Japan that accounts for the location and size of the tumor, organ dysfunction affecting quality of life (mobility restrictions, urinary tract obstruction, bowel obstruction), and complications (abscess and fistula formation and dehiscence of the abdominal wall) (Table 9) [182, 183]. Unlike the Church classification, this new classification can be used at any time point during the clinical course.

**Table 9:** Classification of desmoid tumors. Reproduced with permission from Ishida H, Chikatani K, Mori Y, et al. (2020) Diagnosis, treatment, and proposal of the new classification system for the severity of desmoid tumors associated with familial adenomatous polyposis. J Hered Tumors 20:45–58

Grade	Location	Maximum diameter	Number of tumor	Symptom
1	Extra-abdominal^*^	< 10 cm	1	None–any
2A	Extra-abdominal^a^	≥ 10 cm	Any	None–any
2B	Extra-abdominal^a^	Any	Any	Motion restriction ( +)
3A	Intra-abdominal	< 10 cm	Any	Urinary tract obstruction (-) and Bowel obstruction (-)
3B	Intra-abdominal	≥ 10 cm	Any	Urinary tract obstruction (-) and Bowel obstruction (-)
4A	Intra-abdominal	Any	Any	Urinary tract obstruction ( +) and Bowel obstruction (-)
4B	Intra-abdominal	Any	Any	Incomplete bowel obstruction ( +)
4C	Intra-abdominal	Any	Any	Complete bowel obstruction ( +)
5	Intra-abdominal	Any	Any	Abscess/ Fistula formation/ Abdominal wall dehiscence

^*a*^*Extra-abdominal* abdominal wall, limbs or trunk

##### Surveillance

- Surveillance is important for early diagnosis of desmoid tumors because of the negative treatment outcomes and poor long-term prognosis when these tumors are advanced [181].
- Desmoid tumors frequently develop within 2–3 years after total proctocolectomy, typically in the abdominal wall, mesentery, or retroperitoneum [119, 171, 172]. Therefore, during this period, attention should be paid to clinical symptoms (e.g., abdominal pain, bloating, masses, gastrointestinal passage obstruction), with abdominal palpation every 6 months and imaging studies (contrast-enhanced computed tomography [CT] or MRI of the abdomen and pelvis) annually.
- Regular contrast-enhanced CT or MRI of the abdomen and pelvis is recommended in cases with a personal or family history of desmoid tumors [9].

##### Treatment

Intra-abdominal desmoid tumors detected during surveillance should be managed according to the Church classification [180, 181] or the new Japanese classification [182]. The treatment plan should be considered based on the symptoms of extra-abdominal desmoid tumors (primarily in the abdominal wall) and their impact on day-to-day life.

*Pharmacotherapy*

- There is minimal evidence on pharmacotherapy for desmoid tumors in patients with FAP. Some of the research on desmoid tumors has included patients with FAP, and treatment can generally follow the approach for sporadic desmoid tumors [184].
- Anti-estrogen drugs and NSAIDs
  - Given the high expression of estrogen receptors and cyclooxygenase (COX)-2 in desmoid tumors, anti-estrogen drugs (e.g., tamoxifen, raloxifene, and toremifene) and NSAIDs (e.g., sulindac and indomethacin) have been investigated, mostly in retrospective analyses. A systematic review of anti-estrogen drugs reported a response rate of 51%, with no remarkable difference in efficacy or safety when used as monotherapy and in combination with NSAIDs. A comparison of desmoid tumors between patients with FAP (*n* = 91) and sporadic cases (*n* = 50) found no significant difference in the response rate (51% vs. 48%) or disease control rate (78% vs. 86%) [185]. The only relevant prospective Phase II trial investigated a combination of sulindac and tamoxifen in pediatric patients (20% of whom had FAP) and reported a response rate of 8% and a 2-year progression-free survival rate of 36%, showing less favorable outcomes than in the retrospective reports [186].
- Molecular targeted therapy
  - Several prospective Phase II trials of the tyrosine kinase inhibitor imatinib have reported high disease control rates (84%–91%) despite low response rates (6%–19%) [187–190]. A randomized controlled trial that compared the multikinase inhibitor sorafenib with a placebo found a significant increase in median progression-free survival in the sorafenib group (not reached vs. 11.3 months, hazard ratio 0.13, *p* < 0.001) with a good response rate (33% vs. 20%) [191]. In the randomized Phase II DESMOPAZ trial, which compared the multikinase inhibitor pazopanib with oral methotrexate plus vinblastine, the response rate and 1-year progression-free survival rate were better in the pazopanib group (37% vs. 25% and 86% vs. 67%, respectively) [192].
- Cytotoxic chemotherapy
  - Most reports on cytotoxic chemotherapy are retrospective; however, a systematic review reported response rates of 11%–82% and disease control rates of 71%–100% for low-dose methotrexate plus a vinca alkaloid (vinblastine or vinorelbine) and response rates of 33%–100% and disease control rates of 89%–100% for standard-dose anthracycline-based treatment [193]. A prospective phase II trial of methotrexate plus vinblastine reported response rates of 35%–40% [194, 195]. Retrospective studies have demonstrated the efficacy of doxorubicin plus dacarbazine and low-dose methotrexate plus vinca alkaloids for desmoid tumors in patients with FAP, with respective response rates of 63.6% and 40.3% [196, 197].
- Gamma-secretase inhibitors
  - In November 2023, the US Food and Drug Administration approved nirogacestat, a gamma-secretase inhibitor targeting NOTCH receptors, for adult patients with progressive desmoid tumors requiring systemic therapy. The randomized Phase III DeFi trial evaluated the efficacy of nirogacestat for desmoid tumors and found that progression-free survival was significantly longer in the nirogacestat group than in the placebo group (hazard ratio 0.29). In a subgroup analysis limited to individuals with a family history of FAP, 8 of the 13 patients in the placebo group experienced progression compared with only 2 of the 11 patients in the nirogacestat group; however, there was no significant difference in progression-free survival between the two groups.

*Surgery*

**Fig. 16:**
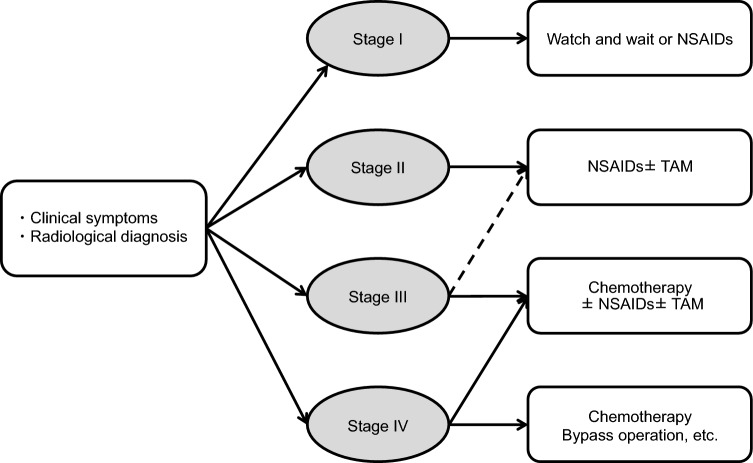
Disease classification by Church, et al. [181] and treatment plans for intra-abdominal desmoid tumors. NSAIDs, nonsteroidal anti-inflammatory drugs, TAM, tamoxifen

- Intra-abdominal desmoid tumors have a recurrence rate of 10%–68% even after complete resection [169], while 5%–33% may spontaneously regress [174–176]. Given the substantial surgical trauma involved [198], surgery should be limited to patients with symptoms such as bowel obstruction [199]. Asymptomatic cases should be managed with observation or pharmacotherapy based on the Church classification [180, 181] or the new Japanese classification [182, 200]. Extra-abdominal desmoid tumors (mostly in the abdominal wall) are also generally managed with observation; however, surgery may be considered if the tumors are having a considerable impact on quality of life, such as by limiting movement.
- Recurrence may occur because of not only incomplete resection but also new tumor formation at the resection site; therefore, excessive resection of tumor margins should be avoided [201].
- Surgery may be considered for gastrointestinal obstruction in patients with intra-abdominal desmoid tumors; however, resection can be challenging and may require extensive bowel resection [199, 202].
- One report suggests no difference in the survival rate between complete resection and non-resection, including bypass procedures [203].
- Data from Japan indicate that surgical resection was performed in 86% of extra-abdominal, 48% of intra-abdominal, and 71% of mixed-type desmoid tumors, with complete resection rates of 91% for extra-abdominal tumors and 46% for intra-abdominal tumors [204].
- An analysis of 26 cases in Japan showed a tendency to treat stage I desmoid tumors with NSAIDs (primarily sulindac) or chemotherapy without surgery. In contrast, surgery was performed in 62.5% of stage III cases, with frequent use of NSAIDs (primarily sulindac), hormone therapy, and chemotherapy [204].
- Although no prospective studies have been conducted, the Church classification [180, 181] suggests observation or NSAIDs for stage I (maximum diameter < 10 cm without size increasing trend for 6 months, no tumor palpation, no symptomatic pain, nor restriction on daily life), NSAIDs + tamoxifen for stage II (maximum diameter < 10 cm without size increasing trend for 6 months, tumor palpation and/or symptomatic pain, without restriction on daily life), chemotherapy ± NSAIDs ± tamoxifen for stage III (maximum diameter between 10 to 20 cm with size increasing trend, with urinary tract and/or intestinal obstruction, with restriction on daily life), and chemotherapy or bypass surgery for stage IV (maximum diameter > 20 cm with more than 50% size increasing within 6 months, with urinary tract and/or intestinal obstruction, necessitating hospitalization). No deaths have been reported for stages I/II, whereas the mortality rates for stages III and IV have been 15% and 44%, respectively [181] (Fig. 16). Stent placement is recommended for ureteral obstruction [181, 205].

##### Postoperative surveillance

Surgical treatment of desmoid tumors (especially the intra-abdominal type) should be avoided whenever possible. However, if surgery is unavoidable, strict postoperative surveillance is necessary to monitor for early recurrence, and regular imaging examinations such as abdominal ultrasound, MRI, and CT scans should be included.

#### Other associated manifestations

In addition to colorectal tumors, patients with FAP may also develop small bowel tumors, hepatoblastomas, adrenal tumors, and thyroid cancer.

##### Small bowel (jejunal and ileal) tumors

- The cumulative risk of small bowel cancer in patients with FAP is < 1% (compared with 0.3% in the general population), with an average age of onset of 43 years [9]. A meta-analysis showed a positive correlation between the occurrence of small bowel tumors and duodenal tumors [206]. The small intestine should be included during CT/MRI monitoring of desmoid tumors; however, no established evidence exists for regular imaging or endoscopic surveillance of the small intestine in patients with FAP [9, 207]. Capsule endoscopy may also be considered, especially in cases with severe duodenal polyposis [9].
- Reports suggest that capsule endoscopy can be completed safely in patients with FAP, even after total proctocolectomy, and in younger patients [207–210]. However, care should be taken to confirm the absence of obstructive lesions before capsule endoscopy and to retrieve the capsule after the examination, particularly in patients who have undergone total proctocolectomy [211]. In patients with FAP, the detection rate of jejunal and ileal polyps using capsule endoscopy ranges from 30.4% to 60% [207–210]. A study of the distribution of jejunal and ileal polyps using capsule endoscopy in 29 patients with FAP found small bowel polyps in 21 cases, with 76% in the proximal jejunum and only 3% in the distal jejunum and ileum [212]. However, the frequency of malignancy arising from small bowel polyps is unknown, and use of capsule endoscopy in the small bowel remains limited [213, 214]. Capsule endoscopy should be considered in patients with FAP when malignancy is suspected in areas not observable by EGD.

##### Adrenal tumors

Adrenal tumors occur in 7.4%–16% of patients with FAP (compared with 1%–3% in the general population) [215–217]. In one study that included 311 patients with FAP, 48 (16%) were identified to have adrenal tumors, with an average age at diagnosis of 45 years and bilateral tumors found in 23% of cases [215]. Most were incidentally discovered on CT scans, with 80% being adenomas and over 97% being benign (including myelolipomas and hyperplasia). Only 1 case (approximately 2%) was cancer.

##### Hepatoblastoma

The incidence of hepatoblastoma is higher in young patients with FAP than in the general population [218]. The cumulative risk ranges from 0.4% to 2.5%, with an average age of onset between 18 and 33 months [9]. A recent review of 109 patients with FAP-associated hepatoblastoma reported a male-to-female ratio of 2.6:1 and a median age of 20 months (ranging from newborn to 11.6 years) [219]. Hepatoblastoma was identified before diagnosis of FAP in 35 cases (out of 49 with available data). De novo occurrence was observed in 12% of cases, with no correlation found between location of the *APC* variant and development of hepatoblastoma. Screening for hepatoblastoma by liver palpation, abdominal ultrasound, and serum alpha-fetoprotein every 3–6 months until the age of 5 years [9] or every 2 years until the age of 7 years [220] has been proposed. However, no high-evidence routine screening method exists for hepatoblastoma [9, 220].

##### Thyroid cancer

The cumulative risk of thyroid cancer is higher in patients with FAP than in the general population (1.2–12% vs. 1.2%) [9]. A recent meta-analysis reported thyroid cancer in approximately 2.6% of patients with FAP, with an average age at diagnosis of 31 years and 95% of cases occurring in females. Papillary carcinoma accounted for 83% of cases, of which 26% were the cribriform-morular variant. In approximately one-third of the cases, thyroid cancer was diagnosed before FAP was identified. The tumor was bilateral in 46% of cases and multicentric in 59%. Furthermore, 79% of pathogenic variants were located at the 5′ end of the *APC* gene [9, 16]. A Japanese study reported a frequency of 6.4% for thyroid cancer in patients with FAP, with a higher prevalence in women (a female to male ratio of 8:1) and an age range of 17–41 years for women and 39–57 years for men, which is consistent with another report [221]. The prevalence of thyroid cancer was significantly higher in groups screened with ultrasound than in those without ultrasound screening (6.9% vs. 1.4%). Therefore, annual thyroid ultrasound examinations starting in adolescence are recommended [9, 16]. Ultrasound should begin in late adolescence, followed by regular surveillance every 2–5 years if the results are normal. Consultation with a thyroid specialist is recommended if abnormalities are detected. Shorter intervals between surveillance examinations should be considered if there is a family history of thyroid cancer [9].

### Management of at-risk relatives and children

#### At-risk relatives

- At-risk relatives (especially first-degree relatives, which include parents, children, and siblings) should receive a thorough explanation of the disease, and gastrointestinal surveillance, particularly of the colon/rectum, should be conducted with their consent.
- If at-risk relatives have colorectal adenomas (especially multiple adenomas), the diagnostic chart for hereditary colorectal polyposis (Fig. 5) should be followed.
- At-risk relatives with clinical symptoms related to FAP should be managed regardless of age.
- In patients with hereditary tumors, including FAP, collection and accurate recording of a family history using standard pedigree documentation is essential [222–224]. Updating family information periodically is recommended because a personal medical history may change.
- Genetic counseling should also be offered to at-risk relatives.
- If a GPV of the *APC* gene has been identified within a family, a diagnosis can be confirmed by genetic testing of normal tissue, such as a blood sample, and the appropriate timing for predictive diagnosis should be considered during genetic counseling. A genetic specialist must provide genetic counseling before and after genetic testing.

#### Management of children

- For classical FAP, consider genetic counseling and predictive diagnosis with parental involvement at the recommended age for starting colorectal surveillance (early to mid-teens). For AFAP, consider this in late adolescence.
- If active hepatoblastoma screening is considered, predictive diagnosis during infancy should also be considered.

##### Pediatric FAP

*Gastrointestinal lesions*

- Multiple adenomas appear in the colon/rectum during adolescence. A registry study in the UK found that the number of colorectal polyps at the time of first total colonoscopy (at an average age of 13 years) was < 100 in 79% of cases, 101–500 in 17%, and > 500 in 4%, with a median maximum polyp size of 2 mm (range, 1–15) [225]. Surveillance after the initial examination found that the number of polyps increased by a mean of 12.5 (range, 0–145) per year. The annual rate of increase in the number of colorectal polyps in pediatric patients with the codon 1309 pathogenic variant has been reported to be more rapid at 89 per year [226].
- CRC is extremely rare in patients under 20 years of age, and occurs in < 0.2% of patients with FAP [30]. However, a total colonoscopy should be performed regardless of age if gastrointestinal symptoms, such as diarrhea or bloody stools, or anemia are present.
- Pediatric endoscopy: Care should be taken to avoid traumatic experiences from endoscopy that would make the child unwilling to undergo future examinations. In view of the varying stages of development and understanding among children in the target age group for FAP surveillance, it is important to explain the procedure and obtain informed assent in a way that is appropriate to the child’s developmental stage. Although bowel preparation is challenging, ascorbic acid-containing polyethylene glycol electrolyte solution is effective and safe [227]. Active use of sedation and appropriately managed anesthesia is also recommended.

*Extra-gastrointestinal lesions*

- Hepatoblastoma: Fewer than 2% of pediatric patients with FAP develop hepatoblastoma and primarily before the age of 3 years [228]. There are differing opinions on whether to recommend screening for hepatoblastoma (abdominal palpation, abdominal ultrasound, and serum alpha-fetoprotein every 3–6 months until 5 years of age [9, 229] or every 2 years until the age of 7 years [220]), depending on whether the effectiveness of screening is supported by evidence. For infants at risk of FAP, parents should be informed about the risk of hepatoblastoma, and the decision should be made considering the parents’ wishes.
- Thyroid cancer, desmoid tumors, brain tumors (e.g., medulloblastoma) may also occur in pediatric patients.

*Congenital hypertrophy of the retinal pigment epithelium (CHRPE)*

CHRPE is a discontinuous flat pigmented lesion of the retina that is present from birth in approximately 80% of patients with FAP. It is asymptomatic and does not require treatment because it does not affect vision or become malignant. Bilateral or multiple CHRPE is a characteristic finding in FAP.

### Clinical questions

#### CQ1: Should genetic testing be performed in patients with colorectal adenomatous polyposis?

(Evidence level: C, Recommendation grade: 1, Agreement rate: 88.9%).

**Recommendation:** Genetic testing must be performed in patients with colorectal adenomatous polyposis for several reasons, including diagnostic guidance, therapeutic decision-making, and aiding in surveillance strategies for the proband as well as diagnosis for at-risk relatives.

##### Comments

Eight clinical guidelines, seven case series studies, and one cross-sectional study on genetic testing for patients with colorectal adenomatous polyposis were reviewed. Patients with FAP often develop adenomatous polyposis in the colon/rectum as a characteristic clinical feature, which means that genetic testing is often omitted. However, certain pathogenic *APC* variants are associated with the density of colorectal adenomas and the occurrence of extracolonic manifestations (e.g., fundic gland polyposis, duodenal adenomas, osteomas, desmoid tumors, and the CHRPE phenotype) in patients with FAP [15, 25, 26, 128]. Therefore, the genotype can be a reference for surveillance, timing of prophylactic colectomy, choice of surgical procedure, and prediction of the risk of postoperative desmoid tumors.

Notably, 20%–25% of FAP cases arise de novo, and there may be individuals with a pathogenic *APC* variant even in the absence of a family history of adenomatous polyposis [230]. In contrast, while relatives who share the same pathogenic variant as the proband also develop colorectal adenomatous polyposis, their colorectal adenomas may not be prominent even in adulthood in families with low adenoma density. Moreover, some individuals hesitate to undergo colonoscopy because of fear or embarrassment, even those who are members of families in which adenomatous polyposis occurs from a relatively young age. For these individuals, genetic testing can be substituted for colonoscopy to diagnose FAP. Surveillance interventions should be initiated if the same pathogenic variant is found in a relative [220].

In some cases, pathogenic *APC* variants are not detected, even when FAP is clinically suspected. Reports from Western countries indicate that pathogenic *APC* variants are found in 60%–70% of patients who have ≥ 100 adenomas; however, pathogenic variants of *APC*, biallelic *MUTYH* are observed in only 10%, 7% of those who have 20–99 adenomas, respectively. The detection rates decrease further to 5% and 4%, respectively, in those who have 10–19 adenomas [33]. A Japanese study in which 123 patients with ≥ 10 adenomatous polyps in the colon/rectum were investigated by next-generation sequencing identified pathogenic *APC* variants in 93% of patients with dense adenomatous polyposis, 72% of those with sparse adenomatous polyposis, and 17% of those with < 100 adenomas, suggesting a correlation between the density of colorectal adenomas and the detection rate of pathogenic *APC* variants [35]. Furthermore, 7.3%–20% of cases with colorectal adenomatous polyposis involve somatic *APC* mosaicism [35, 37]. Somatic *APC* mosaicism may be associated with atypical phenotypes, such as adenomatous polyposis confined to certain areas of the colon/rectum or an absence of FAP-associated manifestations. Diagnosis of somatic mosaicism requires the ability to detect low-frequency variants, for which next-generation sequencing is preferable to traditional Sanger sequencing.

Reasons for not detecting GPVs in the *APC* gene, even when FAP is suspected, may include (1) *APC* variants that are undetectable by the method used, (2) somatic *APC* mosaicism, and (3) MAP, PPAP, or other known or unknown genes that cause colorectal adenomatous polyposis. This tendency is more apparent in patients with oligopolyposis (10–99 adenomas), which is genetically heterogeneous. Therefore, MGPT, which simultaneously analyzes multiple genes associated with hereditary tumors, is recommended for genetic testing in patients with colorectal adenomatous polyposis [231]. Genetic testing for *APC* may be selected in cases where FAP is strongly suspected based on adenoma density, the presence of extracolonic manifestations, or family history. However, MGPT for hereditary tumors should be considered if no pathogenic variants are found.

Western guidelines recommend genetic testing for adolescent patients with colorectal adenomatous polyposis, including those who are school-aged [138, 232, 233]. They also strongly recommend predictive genetic testing for at-risk relatives, including children, if an at-risk relative carries a pathogenic *APC* variant [9, 220, 229]. Genetic testing in patients with colorectal adenomatous polyposis should be considered useful for diagnosis, therapeutic decision-making, and surveillance of the proband, as well as for diagnosis of at-risk relatives.

#### CQ2: Is chemoprevention effective for patients with FAP who have not undergone colectomy and not developed CRC?

(**Evidence level: B**, **Recommendation grade: None**, **Agreement rate: 94.4%).**

**Recommendation:** Chemoprevention has shown some ability to suppress adenoma in patients with FAP who do not have a past history of CRC or colectomy during limited periods of administration and observation, despite the risk of treatment-related adverse events. However, there is insufficient evidence for recommending chemoprevention because of the lack of robust data on its efficacy and long-term safety.

##### Comments

Fifteen randomized controlled trials and one cohort study of chemoprevention in patients with FAP who had not undergone colectomy or developed CRC were reviewed. A manual search of the reference lists for these studies identified four additional studies concerning cardiovascular events related to COX-2 inhibitors.

The chemopreventive effects of sulindac (an NSAID) have been evaluated in patients with FAP. Sulindac was found to suppress the growth and proliferation of colorectal adenomas [234, 235]; however, adenomas tended to increase and enlarge after sulindac was discontinued [235]. A recent retrospective observational study also found that sulindac administered for an average of 7.4 years in 39 patients with FAP and confirmed pathogenic *APC* variants who refused prophylactic colectomy stabilized polyp numbers in all but 1 case [236]. Clinical trials of combined therapies involving sulindac and other NSAIDs (e.g., eflornithine or erlotinib) also found a reduction in the numbers of adenomatous polyps and a delay in the need for colectomy; however, these combination therapies were also associated with adverse events [237–239].

In another study that included 77 patients with FAP, administration of celecoxib (a selective COX-2 inhibitor) at a dosage of 800 mg/day for 6 months resulted in a reduction in the number and size of colorectal adenomas [240]. In pediatric patients with FAP, celecoxib suppressed development of adenomatous polyps without an increase in the adverse event rate above that with placebo [241]. However, a clinical trial that investigated the polyp-suppressing effects of celecoxib in patients without FAP but with a history of colorectal tumors found an increase in cardiovascular adverse events [242]. Other selective COX-2 inhibitors, such as rofecoxib and tiracoxib, have a limited ability to suppress adenomatous polyps [243, 244]; however, like celecoxib, they are associated with an increase in thrombotic cardiovascular events [245].

Eicosapentaenoic acid, derived from fish oil, has been found to decrease the number and size of colorectal adenomas in patients with FAP after IRA [246]. However, it does not reduce the risk of adenoma in the general population [247].

The CAPP1 trial evaluated the chemopreventive effects of high-dose aspirin (600 mg/day) and resistant starch in young patients with FAP (age 10–21 years). However, neither intervention significantly reduced the number of adenomas in the sigmoid colon and rectum [248]. In Japan, a small, double-blind, randomized controlled trial (J-FAPP II) of low-dose aspirin (100 mg/day for 6–10 months) found no significant reduction in the size of adenomas [249]. Subsequently, J-FAPP IV, conducted with a 2 × 2 factorial design comparing low-dose aspirin (100 mg/day) and mesalazine (2 g/day) against placebo, demonstrated a significant reduction in polyp growth after 8 months of low-dose aspirin [250]. Moreover, an economic analysis suggested that combining low-dose aspirin with intensive downstaging polypectomy may be more cost-effective than intensive downstaging polypectomy alone or surgical colectomy in patients who have FAP without dense adenomas but have not previously undergone colectomy [251]. However, administration of low-dose aspirin in healthy older individuals increases the risk of intracranial hemorrhage [252], highlighting the need for clinical trials to assess the long-term impact of low-dose aspirin on progression of the FAP phenotype.

In conclusion, chemoprevention in patients with FAP who have not undergone colectomy or developed CRC has demonstrated adenoma-suppressing effects during limited periods of administration and observation. However, the evidence remains insufficient regarding its effectiveness in avoiding colectomy and preventing cancer and its safety in long-term use.

#### CQ3: Is endoscopic treatment useful for duodenal adenomas, including periampullary adenomas, in patients with FAP?

(Evidence level: C, Recommendation grade: 2, Agreement rate: 100%).

##### Recommendation:

- Endoscopic treatment for non-periampullary duodenal adenomas in patients with FAP is weakly recommended when performed with careful case selection and is a safe treatment method that could potentially avoid surgical intervention.
- Endoscopic treatment for periampullary adenomas in patients with FAP is also weakly recommended for lesions that are clinically significant and warrant treatment.

##### Comments

One prospective cohort study, seven retrospective studies, and one study identified by manual searching were reviewed to evaluate the effectiveness and safety of endoscopic treatment for non-periampullary duodenal adenomas. One systematic review and two retrospective studies were reviewed for periampullary adenomas (ampullary adenomas).

Duodenal adenomas occur in 90% of patients with FAP by the age of 70 years [253], with the incidence of duodenal cancer increasing to 5%–8% by the age of 50–60 years and reaching 18% by the age of 75 years [142, 148, 161, 253, 254]. Duodenal cancer is the second leading cause of death in patients with FAP [138], accounting for 3%–12% of deaths [253, 255], and it is increasing as the proportion of deaths caused by CRC decreases. Periampullary adenoma progresses slowly [153, 256], necessitating use of treatment strategies that are different from those used for non-periampullary adenoma.

*1. Endoscopic treatment for non-periampullary duodenal adenomas in patients with FAP*

The necessity for and use of endoscopic treatment for duodenal adenomas in patients with FAP have been investigated. Until the early 2010s, endoscopic mucosal resection and argon plasma coagulation were commonly performed. However, these procedures are associated with high adverse event rates (5–25%), and 73–100% of patients develop multiple new adenomas in the long term [257, 258]. Consequently, both local and international guidelines have made limited recommendations for endoscopic treatment of duodenal adenomas in patients with FAP [233, 259].

However, since the late 2010s, the introduction of cold polypectomy for small lesions and selective endoscopic treatment targeting only lesions measuring > 10 mm and those with morphological changes have resulted in lower adverse event rates (2%–13%) [157, 260, 261]. The occurrence of invasive or advanced cancer has been minimal after endoscopic treatment of duodenal adenomas, and surgical intervention has been avoided in 74%–100% of cases during follow-up periods of 49–101 months [143, 163, 261]. A prospective interventional study also suggests the possibility of downgrading from Spigelman stage IV [261]. However, many studies are retrospective, with insufficient follow-up durations and a lack of direct comparison with untreated or surgical cases; therefore, the cost-effectiveness of endoscopic intervention remains unclear. Endoscopic treatment for non-periampullary duodenal adenomas should be performed cautiously, considering the risks and benefits, and only by experts. The use of a thin colonoscope, capsule endoscopy, or balloon endoscopy should be considered if polyps are also present distal to the duodenum, including in the horizontal portion [213, 261, 262].

*2. Endoscopic treatment for periampullary adenomas in patients with FAP*

Periampullary adenomas in patients with FAP often progress slowly [153, 256], and biopsies from visually normal ampullas offer little benefit [138, 153]. Treatment for periampullary adenomas should target lesions > 10 mm, those suspected of high-grade dysplasia on endoscopic observation, and those with villous components (tubulovillous or villous adenoma) confirmed by biopsy [256]. Endoscopic ampullectomy is performed for periampullary adenomas with technical success rates of 78%–90%; however, recurrence rates are also high at 25%–33% [263–265]. In one report, 75% of recurrences could be treated with additional endoscopic interventions [264], indicating that endoscopic ampullectomy could potentially avoid surgical intervention. However, the adverse event rates for endoscopic ampullectomy have included bleeding (in 9%–11% of cases), acute pancreatitis (2%–15%), and perforation (2%–4%) [263–265], which means that the procedure should be performed cautiously and only by experts.

#### CQ4: Is surveillance for desmoid tumors useful in patients with FAP?

(Evidence level: C, Recommendation grade: 2, Agreement rate: 94.4%).

**Recommendation:** Surveillance for desmoid tumors in patients with FAP is weakly recommended, especially considering that treatment interventions may become more invasive if the tumors are diagnosed when symptomatic.

##### Comments

One meta-analysis, two systematic reviews, one cohort study, three case–control studies, five case series, three case reports, and eight review articles were reviewed to evaluate the use of surveillance for desmoid tumors in patients with FAP.

Intra-abdominal desmoid tumors are the second leading cause of death in patients with FAP and can affect life expectancy. In a meta-analysis that included 4,625 patients with FAP [15], risk factors for development of a desmoid tumor included a family history (odds ratio [OR] 7.02, 95% confidence interval [CI] 4.15–11.9), GPVs in the *APC* gene located distal to codon 1399 (OR 4.37, 95% CI 2.14–8.91), a history of abdominal surgery (OR 3.35, 95% CI 1.33–8.41), and female sex (OR 1.57, 95% CI 1.13–2.18), with 41% of desmoid tumors occurring intra-abdominally. Furthermore, a registry study from four European countries reported that 53% of desmoid tumors developed after colectomy [179]. A recent meta-analysis of 6,452 patients with FAP also identified a history of abdominal surgery as a risk factor for desmoid tumors (OR 3.40, 95% CI 1.64–7.03). No significant difference in the incidence of desmoid tumors was observed between patients who underwent total colectomy with IRA and those who underwent total proctocolectomy with IPAA or between open surgery and laparoscopic surgery [266]. However, when analyzing among laparoscopic surgery, they reported higher desmoid tumor incidence after laparoscopic IPAA compared to laparoscopic IRA [266]. A multicenter study in Japan reported that 71.8% of desmoid tumors that developed in patients with FAP after colectomy were intra-abdominal [172]. Therefore, surveillance for intra-abdominal desmoid tumors is particularly important after abdominal surgery in patients with FAP.

In terms of timing of onset, several international reports indicate that desmoid tumors occur at a median of 3–4 years after surgery, with some cases reported as late as 9 years postoperatively [15]. A multicenter study in Japan found the median time of onset to be 2.2 years after surgery [172].

Although no prospective studies have directly demonstrated the usefulness of postoperative surveillance for desmoid tumors in patients with FAP, the available evidence suggests that not all desmoid tumors in patients with FAP require intervention. However, more invasive interventions may be necessary if a desmoid tumor is very advanced at the time of diagnosis. Specifically, when symptomatic intra-abdominal desmoid tumors are detected, they are often at an advanced stage, such as stage III using Church’s system [205] or grade 4–5 using Ishida’s severity classification [182], and typically require invasive treatment [183]. Therefore, diagnosing asymptomatic desmoid tumors during postoperative surveillance could potentially allow for less invasive treatment interventions.

In conclusion, given that early diagnosis of asymptomatic desmoid tumors may afford patients the chance to try less invasive treatment, it is weakly recommended to conduct surveillance for desmoid tumors in patients with FAP, particularly after abdominal surgery.

#### CQ5: Is surveillance for thyroid cancer useful in patients with FAP?

(Evidence level: C, Recommendation grade: 2, Agreement rate: 100%).

**Recommendation:** Surveillance for thyroid cancer is weakly recommended for young female patients with FAP because of their increased risk of developing the disease.

##### Comments

One meta-analysis, one systematic review, two case–control studies, one case series, and eight review articles were reviewed for surveillance of thyroid cancer in patients with FAP. Most thyroid cancers in these patients are papillary carcinomas, with > 50% exhibiting the distinctive cribriform-morular variant histology. The incidence of multifocality is high (28.6%–69%), as is that of bilaterality (42%–67%); however, the prognosis is relatively favorable. A meta-analysis that investigated thyroid cancer in FAP analyzed 9,821 patients and found that 79.2% of those who developed thyroid cancer had a GPV in the *APC* mutation cluster region (codons 1286–1513), and 95% of these cases were female [16]. Furthermore, a Japanese study that included 129 patients with FAP who underwent neck ultrasound reported that women under 35 years of age were at higher risk of thyroid cancer [267].

While there are recommendations for thyroid cancer surveillance in patients with FAP using neck palpation and ultrasound, there is no high-level evidence from prospective studies that directly demonstrates its effectiveness. A multicenter study performed by the Colorectal Cancer Study Group that included 282 patients with FAP found that the median age at the time of diagnosis of FAP was 30 years [221]. In contrast, the median age at diagnosis of thyroid cancer was 27.5 years, with some patients being diagnosed with thyroid cancer before the diagnosis of FAP. The cumulative incidence of thyroid cancer was significantly higher in women (11.4%, 16 cases) than in men (1.4%, only 2 cases), and most cases occurred before the age of 50 years. Female sex and age younger than 33 years were independent high-risk factors for thyroid cancer. In the same study, a meta-analysis was conducted that found a 1.6% incidence of thyroid cancer among patients with FAP, with an increasing trend in incidence since 2000. The study recommended considering ultrasound surveillance of the neck from the age of 20 years onward.

International reports also recommend biennial neck ultrasound for patients with FAP and thyroid nodules or other findings because of the increased risk of thyroid cancer [268]. However, there are no data to support use of thyroid surveillance in older patients with FAP, even for women.

In conclusion, surveillance for thyroid cancer using neck palpation and ultrasound is weakly recommended for high-risk patients with FAP, particularly those who are young and female.

## Chapter III. Lynch syndrome

### Overview

Lynch syndrome is a hereditary autosomal dominant disease that is primarily caused by a germline pathogenic variant (GPV) in one of the DNA mismatch repair (MMR) genes (Side Note III-1: Germline epimutation). Lynch syndrome is characterized mainly by colorectal cancer (CRC) and endometrial cancer, which have distinctive pathological features, owing to the breakdown of the MMR mechanism. Various associated tumors can develop in patients and their relatives, necessitating surveillance and treatment.

Side Note III-1: Germline epimutation

Epimutations have been found to contribute to tumorigenesis in some cases of Lynch syndrome. Epimutations are modifications of molecules involved in gene expression, such as aberrant DNA methylation, that can cause changes in gene expression without alterations in the DNA sequence. Although rare, aberrant germline methylation (i.e., hypermethylation) of the promoter region of the *MLH1* has been reported to be a cause of Lynch syndrome [269].

#### Clinical characteristics

- CRC associated with Lynch syndrome differs from sporadic CRC in the following ways:
  o Age of onset is younger.
  o Multiple tumors (synchronous or metachronous) are present.
  o Tumors develop predominantly in the proximal (right-sided) colon.
  o Poorly differentiated adenocarcinoma is more common.
- The histological features of CRC associated with Lynch syndrome include the presence of tumor-infiltrating lymphocytes, a medullary growth pattern, mucinous/signet ring cell differentiation, and a Crohn’s-like lymphocytic reaction [270–273] (Section III-2–1-2: Characteristic histopathological features of CRC with MMR deficiency).
- A variety of other Lynch syndrome-associated extra-colonic cancers, such as endometrial cancer, can also develop (Section III-1–3: Lynch syndrome-associated tumors).

#### Causative genes and mechanism of carcinogenesis

##### Causative genes and mode of inheritance

- *MSH2* on chromosome 2 (2p21-p16)
- *MSH6* on chromosome 2 (2p16.3)
- *EPCAM* on chromosome 2 (2p21)
- *MLH1* on chromosome 3 (3p22.2)
- *PMS2* on chromosome 7 (7p22.1)
- Autosomal dominant inheritance

Lynch syndrome is diagnosed when a GPV is identified in any of these causative genes.

##### Mechanism of carcinogenesis

- In patients with Lynch syndrome, a GPV is present in one allele of an MMR gene. The MMR mechanism becomes compromised when the wild-type allele subsequently mutates (or is methylated in the promoter region), resulting in abnormalities in repeat numbers, which frequently occur in microsatellite regions, which are simple repetitive sequences within the genome. Genes involved in tumor suppression (e.g., *TGFBR2*), cell proliferation, DNA repair (e.g., *MSH3*, *MSH6*), and apoptosis (e.g., *BAX*) contain these repetitive sequences in the coding regions, where alterations tend to develop. The proteins encoded by these MMR genes form heterodimers with specific MMR proteins, which recognize single base substitutions or small insertions/deletions that escape the proofreading function of DNA polymerase during replication.
- *EPCAM*, which is essential for transcription termination, is adjacent to the upstream region of *MSH2*; deletions in the 3′ region of *EPCAM* can lead to Lynch syndrome by causing abnormal methylation in the *MSH2* promoter, resulting in loss of MSH2 protein expression.
- It has been suggested that Lynch syndrome-associated CRC involves a carcinogenesis pathway similar to that in sporadic CRC involving the adenoma-carcinoma sequence. However, many details remain unclear (Fig. 3: Representative mechanism of carcinogenesis in FAP and Lynch syndrome).

#### Lynch syndrome-associated tumors

- Lynch syndrome is associated with various malignancies, including not only CRC but also endometrial, ovarian, gastric, small intestine, bile duct, pancreatic, renal pelvis, ureter, brain, and skin tumors (Side Note III-2: Muir–Torre syndrome [274]). Breast, bladder [275], and prostate cancers [276] have also been reported to develop in association with Lynch syndrome [11]. The risk of associated tumors in Lynch syndrome varies depending on the type of causative gene, type of pathogenic variant, and environmental factors, and is high for CRC and endometrial cancer. However, not all carriers of pathogenic variants will develop Lynch syndrome-associated tumors (Table 10) [271, 277–283].
- In cases with deletions limited to *EPCAM*, the risk of CRC is comparable with that in Lynch syndrome caused by pathogenic variants in *MSH2*, but the risk of endometrial cancer is lower. The risk of malignancy is comparable with that in Lynch syndrome with *MSH2* pathogenic variants when the deletion extends to the 5′ region of *MSH2* [284–286]. *EPCAM* deletions account for 1%–3% of cases of Lynch syndrome [286].

**Table 10:** Cumulative risks for diagnosis by the age of 80

Cancer	Cumulative risks (%)					
	*MLH1*	*MSH2* and* EPCAM*	*MSH6*	*PMS2*	General population in USA^39)^	References
Colorectal	46–61	33–52	10–44	8.7–20	4.1	[10, 334, 410, 429, 433, 434,466–469, 471, 474]
Endometrial	34–54	21–57	16–49	13–26	3.1	[10, 410, 428, 434, 467, 469, 471, 473, 474]
Ovarian	4–20	8–38	≤ 1–13	1.3–3	1.1	[10, 295, 410, 429, 434, 471, 473, 474–472]
Upper urinary tract	0.2–5	2.2–28	0.7–5.5	≤ 1–3.7	–	[280, 410, 429, 434, 471, 473, 474–472]
Bladder	2–7	4.4–12.8	1–8.2	≤ 1–2.4	2.3	[394, 410, 434, 471–471, 474]
Gastric	5–27	0.2–27	≤ 1–7.9	–	0.8	[11, 280, 295, 403, 404, 410, 429, 434, 471, 474, ]
Small bowel	0.4–11	1.1–10	≤ 1–4	0.10–0.3	0.3	[280, 295, 410, 429, 433, 434, 471, 474–471]
Pancreatic	6.2	0.5–1.6	1.4–1.6	≤ 10–1.6	1.7	[410, 434, 471, 474]
Biliary tract	1.9–13	0.02–1.7	0.2 ≤ 1	0.2 ≤ 1	–	[410, 429, 434, 471, 474, 484,485]
Brain tumor	0.7–1.7	2.5–7.7	0.8–1.8	0.6 ≤ 1	0.5	[280, 410, 433, 434, 467, 470, 471, 474]

Side Note III-2: Muir–Torre syndrome

Muir–Torre syndrome is characterized by sebaceous gland tumors (sebaceous adenomas, sebaceous epitheliomas, sebaceous carcinomas) and/or keratoacanthomas in association with various Lynch syndrome-associated tumors, including CRC. GPVs are identified mostly in *MSH2* [274].

#### Epidemiological characteristics

Lynch syndrome has a prevalence of approximately 1 in 279–654 individuals in Western countries [287–289] and accounts for around 2.4%–3.7% of all CRCs [290, 291]. In Japan, Lynch syndrome is reported to accounts for 0.7%–1.01% of all CRCs [292, 293].

### Diagnosis

#### Diagnostic process

**Fig. 17:**
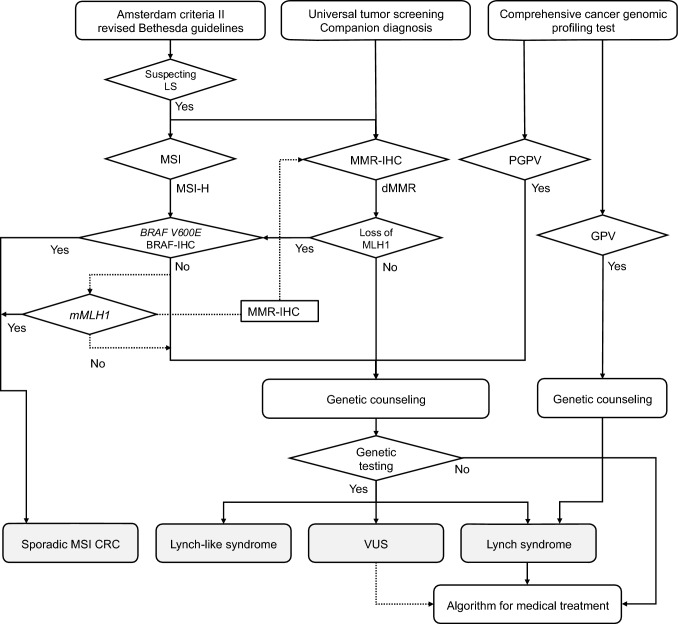
Diagnostic process for Lynch syndrome. CRC, colorectal cancer; GPV, germline pathogenic variant; IHC, immunochemistry; LS, Lynch syndrome; MMR, mismatch repair; mMLH1, aberrant promoter hypermethylation of *MLH1*; MSI, microsatellite instability; MSI-H, high frequency of microsatellite instability; PGPV, presumed germline pathogenic variant; VUS, variant of uncertain significance

The diagnosis should be made according to the following steps in patients with clinicopathological findings (including family history) suggestive of Lynch syndrome (Fig. 17).

**STEP 1:** Confirm the applicable category among (1) to (4):

- **Screening by criteria:** Patient fulfills the Amsterdam criteria II [294] or the criteria in the revised Bethesda guidelines [295]: Lynch syndrome is suspected based on clinical and pathological information (including family history) or the results of microsatellite instability (MSI) testing or immunohistochemistry for MMR proteins (MMR-IHC).
- The Amsterdam criteria II (1999) [294] are strict guidelines requiring that at least three relatives be diagnosed with Lynch syndrome-associated tumors (CRC, endometrial cancer, renal pelvis/ureter cancer, or small bowel cancer) and that all of the following five criteria are met:
  1. At least one of the affected individuals is a first-degree relative of the other two
  2. There are affected individuals in at least two successive generations
  3. At least one of the affected individuals was diagnosed before the age of 50 years
  4. The tumors have been confirmed as malignant on histopathological examination
  5. Familial adenomatous polyposis (FAP) has been excluded.
- The revised Bethesda guidelines [295] are more lenient, recommending MSI testing of tumors collected from individuals who meet any one of the following five conditions:
  1. CRC diagnosed under the age of 50 years.
  2. Synchronous or metachronous CRC or other Lynch syndrome-associated tumors*, regardless of age.
  3. CRC with a high frequency of MSI (MSI-H) histological features** diagnosed under the age of 60 years
  4. CRC in a patient with at least one first-degree relative diagnosed with a Lynch syndrome-associated tumor, with one of them diagnosed under the age of 50 years
  5. CRC in a patient with two or more up to second-degree relatives diagnosed with Lynch syndrome-associated tumors, regardless of age.
- **Universal screening:** Screening tests should be performed for Lynch syndrome using MSI testing or MMR-IHC in all cases with CRC (or those aged < 70 years). If MSI-H or loss of MMR protein expression is observed in the tumor tissue, the patient should be referred for genetic counseling. In cases of CRC where MMR-IHC shows loss of MLH1 and PMS2 protein expression, testing for the *BRAF* V600E variant or *MLH1* promoter methylation can help to exclude sporadic CRC.
- **Companion diagnostics:** Regardless of age, MSI testing or MMR-IHC can be performed as a companion diagnostic for treatment selection in CRC. If MSI-H or loss of expression of the MMR protein is observed in the tumor tissue, the patient should be referred for genetic counseling.
- **Comprehensive genomic profiling**: If a GPV is suspected from a comprehensive genomic profiling test performed on DNA derived from tumor cells, a referral for genetic counseling should be made.

**STEP 2:** Perform MSI testing or MMR-IHC on tumor tissue and confirm MSI-H or loss of MMR protein expression.

**Note:** If IHC reveals that the tumor has the *BRAF* V600E variant in cases with MSI-H or loss of expression of both MLH1 and PMS2 proteins, a sporadic tumor is likely and there is no need to proceed to genetic testing (STEP 3). Notably, a study in patients with CRC associated with Lynch syndrome found that 1.6% (15/969) of cases tested positive for the *BRAF* V600E variant, and 1.7% (8/482) were positive for *MLH1*, 0.7% (2/269) for *MSH2*, 9.3% (5/54) for *PMS2*, and 0% (0/27) for *MSH6* [296].

**STEP 3:** For diagnosis, genetic testing should be performed to identify the GPV in the MMR gene. If a GPV is directly identified by comprehensive genomic profiling of both tumor and non-tumor tissues and the patient wishes to know of the findings, the results should be provided, along with a referral for genetic counseling.

##### Universal screening

- In Western countries, universal screening by MSI testing or MMR-IHC on all CRC cases (or those aged ≤ 70 years) is recommended as a highly sensitive and cost-effective method for diagnosing Lynch syndrome.
- Pooled analyses have indicated that MSI testing, MMR-IHC, or a combination of both 0.93 (95% CI 0.87–0.96), 0.91 (95% CI 0.85–0.95), and 0.97 (95% CI 0.90–0.99), respectively [297].
- The incidence of Lynch syndrome among patients with CRC identified by universal screening is 2.4%–3.7% internationally [290, 291] and 0.7%–1.01% in Japan, suggesting that the efficiency of universal screening may be lower in Japan than in other countries.
- Among older patients with CRC, the prevalence of Lynch syndrome is relatively low but the frequency of sporadic MMR-deficient CRC is higher [293, 298, 299]. Therefore, screening may be targeted to patients below a certain age, such as those aged ≤ 70 years, rather than universally applied in all patients with CRC.
- In families with Lynch syndrome, 15%–27% of members meet the Amsterdam criteria II [298, 300] and 68%–89% meet the revised Bethesda guidelines [298].
- In a study by the Japanese Society for Cancer of the Colon and Rectum, 1.2% of all patients with CRC in Japan fulfilled the Amsterdam criteria II [301]. In a study from Spain, approximately one quarter of patients with CRC fulfilled the revised Bethesda guidelines [302]. Thus, the revised Bethesda guidelines provide high sensitivity but relatively low specificity when screening for Lynch syndrome, whereas the Amsterdam criteria II have lower sensitivity but higher specificity.
- All patients with endometrial cancer should undergo MSI testing or MMR-IHC as part of universal screening.

##### Characteristic histopathological features of CRC with MMR deficiency

**Fig. 18:**
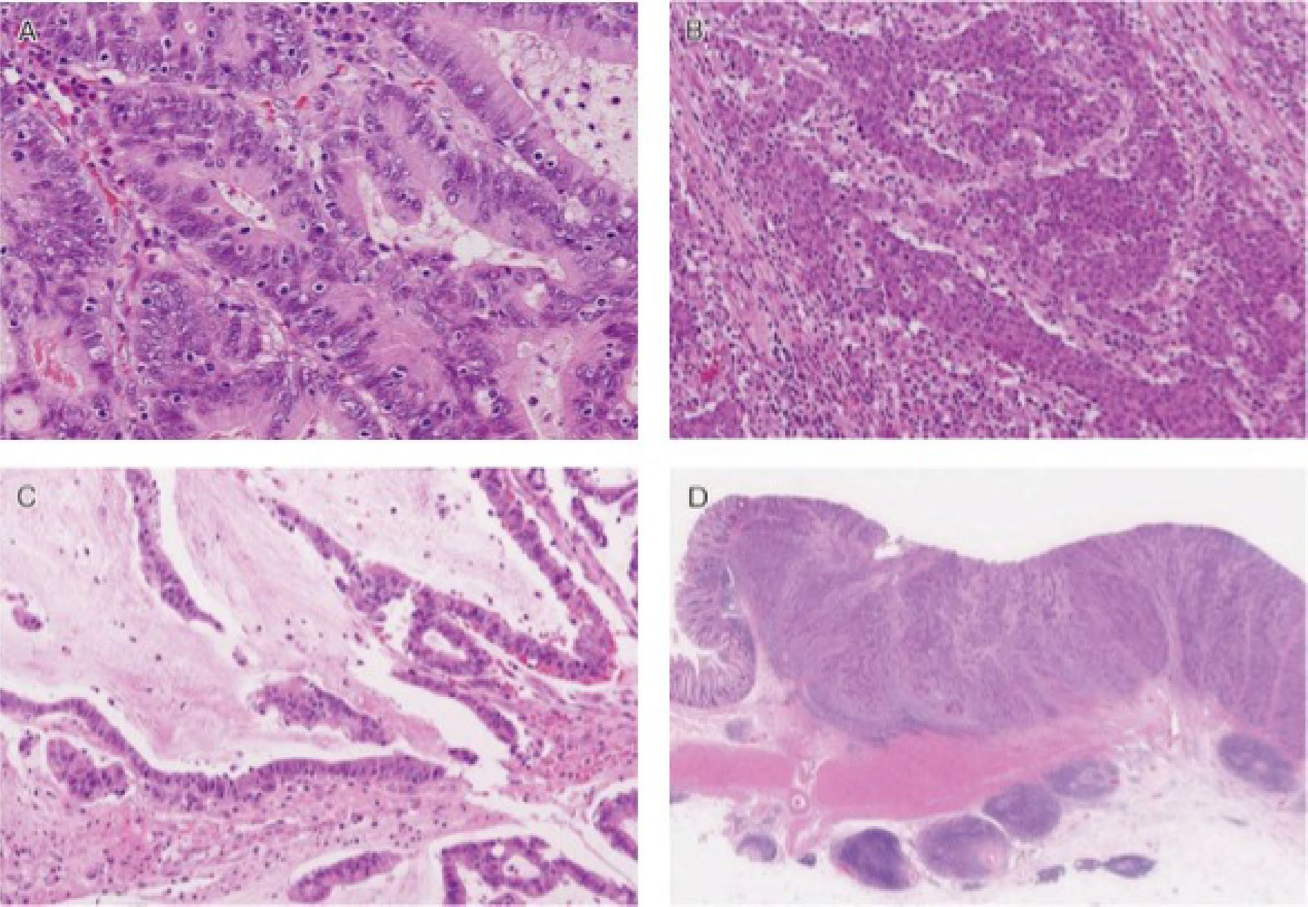
Histologic characteristics of MSI-H colorectal cancer. A Tumor-infiltrating lymphocytes. Numerous intra-epithelial lymphocytes showing clear halos. B Medullary carcinoma. Tumor showing a solid growth pattern without a glandular structure. C Mucinous carcinoma. Prominent extracellular mucin. D Crohn’s-like lymphocytic reaction. Characterized by peritumoral lymphocytic aggregates. MSI-H, high frequency of microsatellite instability

- CRCs with MMR deficiency have several histological features. The revised Bethesda guidelines [295] list the following four characteristics:
  1. Tumor-infiltrating lymphocytes
  2. Medullary growth
  3. Mucinous and signet-ring cell differentiation
  4. A Crohn’s-like lymphocytic reaction (Fig. 18).
- However, these histopathological features are not specific to CRC in patients with Lynch syndrome and are also commonly observed in sporadic MMR-deficient CRC [304].
- In a Japanese study, IHC testing of colorectal tumors from patients with Lynch syndrome identified MMR deficiency in 68 (79%) of 89 cases, regardless of degree of atypia, suggesting that MMR deficiency precedes adenoma formation [305].

##### Screening tests for Lynch syndrome

*MSI testing*

- In tumor cells with MMR deficiency, changes in the repeat number of microsatellites (short tandem repeat sequences in the genome), namely MSI, are more likely to occur.
- Over 90% of CRCs associated with Lynch syndrome are MSI-H [306]. In contrast, the proportion of all CRCs with MSI-H is 12%–16% in Western countries [306–308] and 6%–7% in Japan [309, 310]. Therefore, MSI testing is a useful screening tool for identifying suspected cases of Lynch syndrome.

*MMR-IHC testing*

- In most tumors associated with Lynch syndrome, biallelic inactivation occurs in one of the MMR genes, namely *MLH1*, *MSH2*, *MSH6*, or *PMS2*, leading to loss of the corresponding protein expression in most cases (Side Note III-4: Expression patterns and evaluation; Side Note III-5: Exceptional MMR-IHC expression results).
- Because MSI-H is caused by MMR dysfunction, MSI testing and MMR-IHC show a high concordance rate, which has been reported to be 98.8% in CRC tissues [311].
- The advantage of MMR-IHC over MSI testing is the ability to estimate the causative gene. However, the specificity of these tests is comparable, and the decision as to which to perform should be made based on the testing capabilities of the facility, with either test being sufficient. Although rare, false negatives can occur with either test. Performing the other test can improve the sensitivity of detecting MMR deficiency if a false negative is clinically suspected.

Side Note III-5: Exceptional MMR-IHC results

Abnormal protein expression because of missense variants

Nonfunctional proteins are expressed in some cases with missense variants. This is known to be relatively common in cases of Lynch syndrome with an *MLH1* variant, and only PMS2 expression is lost in most of these cases [312]. However, there are rare cases in which no abnormalities are detected by IHC. In this situation, if the patient is strongly suspected to have Lynch syndrome on clinical grounds, MSI may be detectable by MSI testing.

Secondary variants in MMR genes because of MSI

Some MMR genes contain repeat sequences, which increase the likelihood of secondary variants. In cases with an *MLH1* variant (loss of MLH1/PMS2 protein expression), loss of MSH6 protein expression may be diffuse or follow a regional pattern [313].

Loss of MSH6 protein expression because of preoperative chemoradiotherapy

Preoperative chemoradiotherapy can lead to loss of MSH6 protein expression, even in the absence of germline abnormalities in *MSH6* [313].

*Testing for the BRAF V600E variant*

- The majority of sporadic CRCs with MMR deficiency are caused by loss of MLH1 expression as a result of aberrant hypermethylation of the promoter region of the *MLH1* gene. In contrast, CRCs associated with Lynch syndrome attributable to a GPV in *MLH1* rarely have the *BRAF* V600E variant [296]. Therefore, the presence of the *BRAF* V600E variant in tumor tissue almost excludes the possibility of Lynch syndrome in cases where MMR-IHC reveals loss of expression of both the MLH1 and PMS2 proteins [314].
- Although *BRAF* V600E variants are rare in CRCs associated with *MLH1*, *MSH2*, and *MSH6* pathogenic variants in Lynch syndrome, some CRCs with a GPV in *PMS2* have been reported to harbor the *BRAF* V600E variant, with a prevalence of 9.3%, which underscores the need for caution [296].
- Identifying sporadic tumors using the *BRAF* V600E variant is only effective for CRC and cannot be applied to other types of cancer, such as endometrial cancer.

*Testing for methylation of the MLH1 promoter*

Most sporadic CRCs with MMR deficiency are caused by aberrant hypermethylation of the *MLH1* promoter, which suppresses MLH1 protein expression. Therefore, testing for methylation of the *MLH1* promoter can efficiently identify sporadic CRC and exclude it through genetic testing [9, 231, 232, 315, 316].

#### Diseases and conditions in the differential diagnosis

##### Sporadic MSI-H CRC with aberrant hypermethylation of the *MLH1* promoter.

Sporadic MSI-H CRC has certain typical clinicopathological characteristics, including occurrence in older women, poorly differentiated adenocarcinoma, and a right-sided predominance. The primary cause of MSI-H in sporadic CRC is acquired aberrant hypermethylation of the *MLH1* promoter region [317]. Such tumors show loss of MLH1 and PMS2 protein expression on MMR-IHC. As noted earlier, 50%–67% of CRCs with aberrant hypermethylation of the *MLH1* promoter also harbor the *BRAF* V600E variant [293, 318].

##### Polymerase proofreading associated polyposis

Polymerase proofreading associated polyposis (PPAP) [52, 55, 319] can present with clinical features similar to those of attenuated FAP and Lynch syndrome, necessitating differential diagnosis (II-2-2: Diseases/conditions requiring differential diagnosis). PPAP often involves endometrial cancer, making it a differential diagnosis for Lynch syndrome. CRC caused by *POLE*, the gene responsible for PPAP, can also be MSI-H.

##### Lynch-like syndrome

Lynch-like syndrome refers to CRC in which an MMR defect (MSI-H or loss of MMR protein expression) is found without aberrant hypermethylation of the *MLH1* promoter and is not diagnosed as Lynch syndrome based on genetic testing. The primary cause of this syndrome is somatic variants in both alleles of the MMR gene; however, cases involving undetectable germline MMR gene variants or variants in genes other than MMR may also be included [320, 321].

##### Constitutional mismatch repair deficiency syndrome

Constitutional mismatch repair deficiency (CMMRD) syndrome is caused by biallelic GPVs in an MMR gene, leading to early onset of multiple synchronous or metachronous cancers, including CRC, during childhood [322]. CMMRD follows an autosomal recessive mode of inheritance, with *PMS2* and *MSH6* being the genes most commonly affected. Non-tumor manifestations frequently include café-au-lait spots resembling those observed in neurofibromatosis type 1 [322, 323]. Patients with *PMS2* pathogenic variants may also present with immunodeficiency owing to class switch recombination defects, which are characterized by decreased IgG and IgA levels and an increased IgM level [324]. In a prospective registry study, the median age at first diagnosis of cancer was 9.2 years (range 1.7–39.5), with brain tumors being the most common, followed by CRC and hematological malignancies [325]. The colon typically shows multiple adenomas, with clinical features resembling those of FAP. A surveillance protocol that includes brain and whole body magnetic resonance imaging, abdominal ultrasound, gastrointestinal endoscopy, and blood tests has been proposed [326], and the potential for improved survival outcomes were demonstrated in a full-surveillance cohort [325]. The condition formerly known as Turcot syndrome type 1 is now understood to be CMMRD syndrome.

##### Familial CRC type X

Familial CRC type X (FCCTX) is a term proposed for cases fulfilling the Amsterdam criteria I [327] but lacking germline MMR gene variants and showing intact MMR function in colorectal tumors. FCCTX likely represents a heterogeneous group of conditions, including (1) coincidental clustering of sporadic CRCs, (2) clustering caused by shared lifestyle factors, and (3) hereditary cancers other than Lynch syndrome, including those caused by unidentified genes [328]. Families with FCCTX have a significantly lower risk of Lynch syndrome-associated extra-colonic tumors [329].

### Surveillance and treatment

#### Colorectal adenomas and cancer

##### Characteristics and classification

- Colorectal adenomas in Lynch syndrome show higher degrees of dysplasia than conventional adenomas [330] and progress to cancer more rapidly [294, 331, 332]. The carcinogenesis pathway in Lynch syndrome may involve MMR deficiency from the adenoma stage and an alternative pathway originating from MMR-deficient crypts in addition to the conventional adenoma–carcinoma sequence [305, 333]. However, distinguishing between these pathways during colonoscopy is challenging. Therefore, any neoplastic lesion should be considered for endoscopic treatment regardless of size [330]. A thorough personal medical history should also be taken to identify any associated extra-colonic tumors characteristic of this condition [9, 294] (Table 11).
- Surveillance and screening for Lynch syndrome-associated extra-colonic tumors requires a different approach to that used in the general population. Regular screening of the relevant organs is essential, as is colonoscopy surveillance.

**Table 11:** Recommended surveillance protocols for common Lynch syndrome-associated tumors

Site	Test	Age at the start of tests	Intervals	Comments	References
Colorectum	Colonoscopy	*MLH1, MSH2*: 20–25 years	1–2 years		[9, 229, 231, 232, 315, 316]
		*MSH6*: 30–35 years		-	
		*PMS2*: 30–35 years	1–3 years		
Uterus	Endometrial biopsy	*MLH1, MSH2, MSH6*: 30–35 years	1–2 years	-	[9, 229, 231, 232, 315, 316]
Ovary	Transvaginal US			Consider at physician’s discretion	[9, 315]
Stomach/ duodenum	HP infection	30–35 years		Sterilization if HP infection is present	[9, 229, 232, 315, 316]
	Upper gastrointestinal endoscopy	30–35 years	1–3 years	Consider in cases with high risk factors or family history of gastric cancer	[9, 229, 232, 315, 316]
Urinary tract	Urinalysis or urine cytology	30–35 years	1 years	Consider in cases with pathogenic variants in the *MSH2* or family history of urothelial cancer	[9, 229, 231, 232, 316]
Pancreas	EUS or MRI/MRCP	50 years	1 years	Consider in cases with family history of pancreatic cancer	[9, 10, 229, 411–414]

US, ultrasonography; HP, Helicobacter pylori; EUS, endoscopic ultrasonography; MRI, magnetic resonance imaging; MRCP magnetic resonance cholangiopancreatography

##### Cancer surveillance and prevention

*Colonoscopy surveillance*

The recommended starting age for colonoscopy surveillance in individuals with Lynch syndrome who have not yet developed CRC is generally between 20 and 25 years for those with *MLH1* or *MSH2* variants and between 30 and 35 years for those with *MSH6* or *PMS2* variants [9, 207, 334, 335]. Lifelong regular colonoscopy surveillance is necessary for those who have undergone surgery for CRC because of the risk of metachronous cancer. Although a prospective study in Lynch syndrome families demonstrated that 3-yearly colonoscopy surveillance reduced mortality from CRC by 65% [336], some observational studies have reported cases of advanced cancer developing within the 3-year interval, leading to proposals for shortening the surveillance interval to 1 year [229, 328, 337]. However, a study that compared surveillance intervals of 1–3 years in three European countries (Germany, the Netherlands, and Finland) found no significant difference in cancer incidence or stage at diagnosis according to surveillance interval, and there is still no consensus on the optimal interval [338, 339]. Risk stratification based on the causative gene and history of CRC is being explored. In Japan, the recommended interval is generally 1–2 years [229, 231, 232, 315], whereas a number of European and US guidelines suggest a 2-year interval [9, 207]. High-quality colonoscopy using a high-resolution endoscope is important for surveillance, and adherence to quality indicators for colonoscopy (e.g., quality of bowel preparation, cecal intubation rate, adenoma detection rate, examination time) is also essential [335, 340].

*Lifestyle modifications*

Several lifestyle factors related to diet, alcohol consumption, and physical activity are modifiable and can decrease the risk of CRC in patients with Lynch syndrome. Maintaining an appropriate body weight and smoking cessation are particularly important. High body mass index has been shown to increase the risks of developing adenomas and CRC [315], so it is recommended to maintain body weight within the normal range. A prospective cohort study identified that men with a body mass index of > 25 were at increased risk of CRC [341]. Moreover, randomized controlled trials found a 3.72-fold increase in risk of CRC in obese individuals with the *MLH1* variant but no increase in risk in their counterparts taking aspirin 600 mg/day or those with *MSH2* or *MSH6* variants [342, 343]. Case-control and retrospective observational studies have indicated that smoking also increases the risk of CRC [315, 329, 344]; therefore, smoking cessation is recommended. Notably, current smokers have a higher risk of developing colorectal adenomas than former smokers [345]. Multivitamins and calcium supplements reduce the risk of CRC [346], as does increased fruit consumption [347, 348]. Alcohol consumption is associated with an increased risk of an earlier onset of CRC [348–350], and increased physical activity may reduce the risk of CRC [351].

*Chemoprevention*

Chemoprevention trials using aspirin in patients with Lynch syndrome have been conducted, but its effectiveness of low-dose aspirin remains unclear.

##### Treatment

*Surgery*

- Surgical options for CRC in patients with Lynch syndrome include the following:
  o Resection equivalent to that for sporadic CRC
  o Extended surgery (total colectomy, proctocolectomy)
- The extent of resection is typically equivalent to that for sporadic CRC in Lynch syndrome. When selecting the surgical approach for Lynch syndrome, it is important to consider the location of the primary cancer, the gene responsible, the frequency and timing of colorectal tumors, and other risk factors. Currently, risk stratification for multiple CRCs is inadequate, and no systematic recommendation exists for a specific surgical approach. In Japan, the surgical approach for initial and metachronous CRC is individualized, taking into account the patient’s age, comorbidities, and other factors. The need for postoperative surveillance and its limitations should be explained in detail and the patient’s preferences ascertained before proceeding with surgery [207, 352].
- In Western countries, extended surgery, such as total colectomy for colon cancer and proctocolectomy for rectal cancer, is recommended for patients with Lynch syndrome and *MLH1* or *MSH2* variants. However, there is not sufficient evidence of oncological benefit to recommend extended surgery in cases of CRC with *MSH6* or *PMS2* variants [207, 335].
- In terms of prophylactic colorectal resection, while the lifetime risk of CRC in Lynch syndrome is 54%–74% for men and 30%–52% for women, some variant carriers never develop CRC. Therefore, no consensus exists on the usefulness of prophylactic colorectal resection, and it cannot be recommended for asymptomatic individuals.

Side Note III-6: Extended surgery for CRC in Lynch syndrome

In Lynch syndrome, initial CRCs associated with the *MLH1* and *MSH2* variants have been reported to occur in the proximal colon in 84% of cases with a 36% risk of developing metachronous CRC between the ages of 40 and 70 years [353]. Retrospective observational studies and meta-analyses worldwide have shown that metachronous CRC occurs in 22.4%–22.8% of cases after segmental colectomy, compared with 4.7%–6.8% following extended surgery, indicating a significantly increased risk of metachronous CRC with segmental colectomy [354, 355]. However, no difference in mortality was observed between the two surgical approaches, with a relative risk of death after segmental colectomy of 1.65 (95% CI 0.90–3.02) [355]. Approximately 15% of initial CRCs in Lynch syndrome occur in the rectum, and many metachronous CRCs occur in the right colon in cases where rectal resection is performed. A retrospective observational study reported a cumulative incidence of multiple metachronous CRCs of 19% at 10 years, 47% at 20 years, and 69% at 30 years when endoscopic surveillance was conducted at an average interval of 14 months [356]. Reports on surgical procedures for initial CRC in Japan are limited. However, a favorable prognosis comparable with that for sporadic CRC can be achieved even with resection if quality indicators for colonoscopic examination are adhered to and endoscopic resection of precancerous lesions, including adenomas, is performed [173, 357].

*Adjuvant therapy post-surgery*

- The efficacy of adjuvant chemotherapy specifically for CRC in Lynch syndrome is still unclear; therefore, treatment is often based on the approaches used for sporadic MSI-H CRC. It is important to note the differences between Lynch syndrome-associated CRC and sporadic MSI-H CRC, such as the frequency of *BRAF* V600E variants and methylation status. For example, it has been found that while 5-fluorouracil-based adjuvant chemotherapy is not effective for sporadic MSI-H CRC, it could be beneficial for MSI-H CRC in individuals under 50 years of age who are suspected of having Lynch syndrome [358]. This finding suggests the need to consider Lynch syndrome-associated CRC separately from sporadic MSI-H CRC. However, little useful data are available on adjuvant chemotherapy for Lynch syndrome-associated and sporadic MSI-H rectal cancer.
- A meta-analysis that focused on the relationship between MSI status and the efficacy of 5-fluorouracil-based adjuvant chemotherapy in patients with stage II/III sporadic CRC found that while MSI-H CRC had a better prognosis than microsatellite-stable (MSS) CRC, adjuvant chemotherapy did not improve overall survival or relapse-free survival [359, 360]. However, in the phase III NSABP-C07 and MOSAIC trials, adding oxaliplatin to adjuvant chemotherapy was beneficial in MSI-H and MSS CRCs [361]. Furthermore, an analysis of the ACCENT database, which includes data from 12 clinical trials, showed that progression-free survival and overall survival were better in patients with stage III CRC who received combination therapy with oxaliplatin than in those who received fluoropyrimidine alone, regardless of MSI status [362]. N2 MSI-H/ deficient mismatch repair (dMMR) CRC had a similar prognosis to that of MSS/pMMR CRC, whereas N1 MSI-H/dMMR CRC had a better prognosis than that of MSS/pMMR CRC. Therefore, the indication for adjuvant chemotherapy should not be determined based on MSI status in stage III CRC. Moreover, the benefit of adjuvant therapy is low and its use has not been established in stage II CRC, particularly in MSI-H cases, which generally have a good prognosis.

*Chemotherapy for unresectable advanced/recurrent CRC*

- The frequency of MSI-H is lower in sporadic stage IV CRC than in stage II/III CRC [363, 364]. While chemotherapy specific to advanced or recurrent MSI-H CRC has been well studied, few studies have focused specifically on Lynch syndrome. The phase II KEYNOTE-016 trial, which evaluated the efficacy of pembrolizumab as third-line or later treatment in MSI-H/dMMR CRC, MSI-H/dMMR non-colorectal solid tumors, and MSS CRC, reported response rates of 40%, 71%, and 0%, respectively [365], demonstrating the efficacy of anti-programmed cell death protein 1 antibodies in MSI-H/dMMR solid tumors. Follow-up data for 86 cases of MSI-H/dMMR solid tumors across 12 types of cancer showed a response rate of 53% (52% in CRC and 54% in non-CRC), with response rates of 46% for Lynch syndrome-associated cancers and 59% for cancers not associated with Lynch syndrome [366, 367].
- The efficacy of pembrolizumab as second-line or later chemotherapy in patients with MSI-H/dMMR CRC and those with previously treated non-CRC was also confirmed in the phase II KEYNOTE-164 and KEYNOTE-158 trials [367, 368]. These trials have established pembrolizumab as a treatment option that can be administered for MSI-H/dMMR solid tumors across multiple organ systems.
- The phase II CheckMate-142 trial evaluated the efficacy of nivolumab monotherapy or nivolumab plus ipilimumab combination therapy as second-line or later treatment for MSI-H/dMMR CRC. The response rate was 31% with nivolumab monotherapy and 55% with the combination therapy. Grade 3 and 4 treatment-related adverse events were reported in 20% and 32% of patients, respectively [369, 370]; among these cases, 36% and 29% of the cancers were associated with Lynch syndrome, with response rates of 33% and 71%, respectively, which were comparable with the overall results.
- Progression-free survival was significantly prolonged in the pembrolizumab group in the phase III KEYNOTE-177 trial, which compared the efficacy and safety of pembrolizumab with those of chemotherapy as first-line treatment for MSI-H/dMMR CRC [371]. Approximately 90% of Lynch syndrome-associated CRCs are MSI-H/dMMR. Therefore, use of immune checkpoint inhibitors is strongly recommended for advanced or recurrent CRC in patients with Lynch syndrome.

#### Gynecological tumors

##### Characteristics and classification

- Lynch syndrome-associated gynecological malignancies include endometrial cancer and ovarian cancer. Endometrial cancer is the second most common cancer after CRC in women with Lynch syndrome, with a penetrance of up to approximately 60% [9, 372–374]. Furthermore, endometrial cancer often precedes CRC in women with Lynch syndrome, making it a sentinel malignancy in these patients. Like CRCs, universal screening for Lynch syndrome using MSI testing or MMR-IHC on all endometrial cancers is recommended as a sensitive and cost-effective diagnostic method.
- Notably, the age of onset and cumulative risk of developing endometrial or ovarian cancer in women with Lynch syndrome vary depending on the type of MMR gene involved [9, 315, 375].
- Women with pathogenic variants in *MSH6* develop endometrial cancer at an older age than those with *MLH1* or *MSH2* variants; however, the risk of developing this cancer is similar to or higher than that in women with *MLH1* or *MSH2* variants [315, 334, 353]. There is little evidence to suggest an increased risk of ovarian cancer in women with pathogenic variants in *PMS2*.
- In Japan, endometrial cancer in women with Lynch syndrome is often of the well-differentiated endometrioid type, with many cases presenting at clinical stage I. These patients develop cancer at a younger age, are not obese, and have lower rates of hypertension and dyslipidemia [376]. However, international studies have reported cases involving serous carcinoma, clear cell carcinoma, and carcinosarcoma, collectively known as type II endometrial cancer. Furthermore, endometrial cancer limited to the lower uterine segment occurs more frequently in patients with Lynch syndrome [377, 378]. The prognosis of endometrial cancer in Lynch syndrome is better than that of sporadic cases, likely because of its MSI-H status and the efficacy of immune checkpoint inhibitors [339, 379]. Ovarian malignancies in Lynch syndrome present as non-serous cancers such as endometrioid or clear cell carcinoma at stage I, which contrasts with the high-grade serous carcinomas typically observed in hereditary breast and ovarian cancers. Ovarian cancer in Lynch syndrome also has a better prognosis than sporadic ovarian cancer, with a higher incidence of concurrent endometrial cancer [380, 381].

##### Surveillance

- Endometrial biopsy, which has high sensitivity and specificity, is the primary surveillance method for endometrial cancer [9, 375]. While endometrial cytology is generally not a substitute for biopsy because of its lower diagnostic accuracy, it may be performed at the discretion of the physician, particularly when the biopsy procedure is invasive. The sensitivity of transvaginal ultrasound is approximately 34%, compared with 57% for endometrial biopsy [375]. Transvaginal ultrasound is not recommended for routine surveillance because of the variation in endometrial thickness according to the menstrual cycle in premenopausal women [9, 315]. Moreover, there is limited evidence to suggest that surveillance significantly reduces cancer-related mortality compared with diagnosis after symptom onset, considering that endometrial cancer generally has a good prognosis. While there is no strong evidence regarding the optimal age to start screening or screening intervals, surveillance starting between the ages of 30 and 35 years or 5 years earlier than the youngest age of cancer diagnosis in the family, with intervals of 1–2 years, is often considered. Furthermore, because the age of onset and cumulative risk vary according to the causative gene, surveillance should start at the age of 35 years for *MLH1* and *MSH2* and at the age of 40 years for *MSH6* [9, 382]. Also crucial is education on the importance of seeking a gynecological consultation in the event of abnormal vaginal bleeding, which is the main symptom of endometrial cancer.
- No established effective surveillance method or interval exists for ovarian cancer. However, transvaginal ultrasound and measurement of serum cancer antigen 125 may be considered at the discretion of the physician [9, 315]. Consideration should be given to the risk of interval cancers, namely, cancers that develop and are detected after the last negative surveillance visit but before the next scheduled visit [315]. Although ovarian cancer is often asymptomatic in the early stages, patients should be encouraged to seek a gynecological consultation if they experience symptoms such as lower abdominal pain, bloating, an increase in waist circumference, eating problems, or frequent or urgent urination, given that these are symptoms associated with ovarian cancer. A recent prospective study in patients with Lynch syndrome reported 10-year survival rates of 98% for endometrial cancer and 89% for ovarian cancer [353]. However, whether these outcomes were attributable to the effectiveness of surveillance or the relatively low malignancy of gynecological cancers associated with Lynch syndrome remains unclear.

##### Treatment

*Surgery*

- In general, surgical management of endometrial and ovarian cancers in women with Lynch syndrome should follow the same principles as those for sporadic cases.
- While prophylactic hysterectomy does not reduce mortality in women with Lynch syndrome, it does prevent occurrence of endometrial cancer and should be considered as a risk-reducing option [9, 229, 321].
- In the absence of an effective surveillance method for ovarian cancer, risk-reducing salpingo-oophorectomy (RRSO) may be considered as a primary preventive measure [315]. However, no reduction in ovarian cancer-related mortality has been demonstrated for RRSO [9, 229, 382, 383]. The timing of RSSO should be determined individually, considering factors such as the desire for childbearing, comorbidities, family history, and the causative MMR gene. However, performing hysterectomy and RRSO before the age of 40 years has limited benefits [384]. Concurrent hysterectomy and RRSO may be considered in women with Lynch syndrome undergoing surgery for CRC [315].
- Depending on the timing of RRSO, concerns may arise regarding menopausal symptoms, changes in libido, lipid profile, and bone metabolism as consequences of ovarian depletion. Therefore, it is essential to involve experts in women’s health when considering RRSO. Hormone replacement therapy may be beneficial after RRSO in women without a history of breast cancer [9, 384–386].
- Occult cancer can be detected in resected specimens obtained by RRSO, with reported rates of approximately 5%–17% for endometrial cancers and 0.5% for ovarian cancers. Therefore, a thorough evaluation before surgery is recommended [375, 383, 387, 388].

*Chemoprevention*

Chemoprevention may be considered in addition to risk-reducing surgery. Chemoprevention with high-dose aspirin is effective for CRCs. Oral contraceptives are also beneficial for preventing endometrial and ovarian cancers [9, 389]. However, evidence from Japan is still lacking.

##### Postoperative surveillance

No specific guidelines or recommendations exist regarding the method or duration of postoperative gynecological surveillance after risk-reducing surgery for endometrial and ovarian cancers. Although cases of primary peritoneal cancer after RRSO have been reported in patients with Lynch syndrome [390], the frequency of primary peritoneal cancer after RRSO in these patients remains unclear, and long-term follow-up is recommended.

#### Urological tumors

##### Characteristics and classification

- Upper tract urothelial carcinoma (UTUC) occurs in the renal pelvis and ureter and is a relatively rare type of urological cancer. However, UTUC is involved in approximately 5% of urological cancers and is the third most common Lynch syndrome-associated tumor after CRC (63%) and endometrial cancer (9%) [391–393]. Bladder cancer has recently been recognized as an associated tumor, and associations with prostate cancer, testicular tumors, and adrenocortical carcinoma have been reported [313, 394–399].
- A screening study for UTUC, including sporadic cases, found that 5% of cases had GPVs in MMR genes [393, 400, 401]. Therefore, screening for Lynch syndrome using MMR-IHC is increasingly being conducted for CRCs, endometrial cancers, and UTUC. Furthermore, 64% of Lynch syndrome-associated UTUC cases had a history of other Lynch syndrome-associated tumors, including CRC, underscoring the importance of considering Lynch syndrome in the clinical evaluation of UTUC and taking a thorough medical history [400]. The average age of onset is 70 years for sporadic UTUC and 64 years for Lynch syndrome-associated UTUC, with a higher cumulative incidence in *MSH2* GPV carriers[400].

##### Surveillance

Surveillance of the urinary tract, including urinalysis and urine cytology, is proposed annually for patients with Lynch syndrome, starting from the age of 30–35 years. Ultrasound examinations should be considered in patients with GPVs in *MSH2* [402]. Further evaluation with CT, retrograde pyeloureterography, and tumor biopsy via ureteroscopy is warranted if abnormalities are detected. Retrograde pyeloureterography and ureteroscopic tumor biopsy have high diagnostic accuracy but are invasive procedures that involve insertion of a catheter or ureteroscope into the ureter, making them unsuitable for regular surveillance.

##### Treatment

Treatment for UTUC in Lynch syndrome follows the same principles as those for sporadic UTUC. Depending on imaging findings and disease stage, surgical options with curative intent, such as nephroureterectomy, segmental ureterectomy, or ureteroscopic laser ablation, are considered, and chemotherapy or immune checkpoint inhibitors are options for metastatic cases [485].

#### Upper gastrointestinal tumors

##### Characteristics and classification

The cumulative risk of gastric cancer in Lynch syndrome is particularly high in East Asia, including Japan [11, 295, 403–405]. This high risk may be attributable to regional differences in the prevalence of *Helicobacter pylori* (HP) infection-associated gastritis, with a history of HP infection being a common risk factor for development of gastric cancer, as in the general population [404, 405]. Other related factors include a higher risk of gastric cancer in patients who harbor pathogenic variants in *MLH1* and *MSH2* [406].

##### Surveillance

- Surveillance with upper gastrointestinal endoscopy every 1–3 years is recommended for patients with Lynch syndrome, similar to individuals with HP infection in regions with a high incidence of gastric cancer, like East Asia, and for patients with Lynch syndrome who have a family history of gastric cancer [407]. The typical age of onset of gastric cancer in Lynch syndrome is in the 40 s, with the youngest cases reported being aged 31–35 years. Therefore, surveillance should start at 30–35 years [11, 404, 405].
- Surveillance for small bowel cancer in Lynch syndrome is not recommended owing to its rarity, and current guidelines do not support routine small bowel endoscopy, video capsule endoscopy, or contrast-enhanced CT. Careful observation of the deep duodenum during upper gastrointestinal endoscopy is suggested instead [408].

##### Treatment

*Endoscopic and surgical treatment*

The approach to endoscopic treatment for early gastric cancer and surgical procedures for advanced gastric cancer in Lynch syndrome should follow the same principles as those for sporadic cases.

*Chemotherapy*

The phase III comparative CheckMate-649 trial evaluated the efficacy and safety of nivolumab plus chemotherapy and nivolumab plus ipilimumab versus chemotherapy alone as first-line treatment in patients with advanced gastric cancer [409]. Compared with the group that received chemotherapy alone (*n* = 792), there was a significant overall survival benefit in the group that received nivolumab plus chemotherapy (*n* = 789) (hazard ratio [HR], 0.79) but not in the group that received nivolumab plus ipilimumab. In a subgroup analysis of patients with MSI-H, the overall survival benefit was more pronounced in the group that received nivolumab plus chemotherapy (*n* = 22) than in the group that received chemotherapy alone (*n* = 21) (HR, 0.38). The group that received nivolumab plus ipilimumab (*n* = 11) also showed an overall survival benefit when compared with the chemotherapy group (*n* = 21) (HR, 0.28). Therefore, nivolumab plus chemotherapy is currently used as the standard first-line treatment for advanced gastric cancer, with Lynch syndrome-associated advanced gastric cancer being a particularly promising target for the added benefit of nivolumab.

#### Other associated tumors

- Limited evidence exists regarding the optimal age to start screening for pancreatic cancer in Lynch syndrome or on the screening interval. However, the guidelines developed in Western countries suggest starting surveillance with endoscopic ultrasound or magnetic resonance imaging/cholangiopancreatography at the age of 50 years or 10 years younger than the earliest age of cancer onset in the family for carriers of pathogenic variants with a family history of pancreatic cancer [9, 229, 410–414].
- Limited and inconclusive evidence exists for the surveillance of biliary tract cancer and brain tumors in Lynch syndrome [408].

### Patients with CRC and unconfirmed lynch syndrome status

#### Cases without genetic testing or with variants of uncertain significance

- Surveillance of associated tumors should be tailored to the individual based on clinical information and on MSI and MMR-IHC results if Lynch syndrome is suspected but not confirmed (Fig. 17). “Unconfirmed” refers to cases where genetic testing has not been performed or where no pathogenic variant has been identified (including cases where only variants of uncertain significance are detected).
- Surveillance is indicated if Lynch syndrome is confirmed in cases that meet the Amsterdam criteria II and those who have a strong personal or family history suggestive of Lynch syndrome if MSI testing shows MSI-H or MMR-IHC tests shows loss of MMR protein expression.
- Lynch syndrome cannot be excluded in cases that meet the Amsterdam criteria II or have a strong personal or family history suggestive of Lynch syndrome but MSS/MSI-Low or no loss of MMR protein expression is observed (i.e., no strong evidence of MMR gene abnormalities). In such cases, continued surveillance with attention to personal and family history is recommended, with colonoscopy every 3–5 years at a minimum for CRC.
- If MSI-H or loss of MMR protein expression is detected in cases that meet the revised Bethesda guidelines but not the Amsterdam criteria II or in those with no strong personal or family history suggestive of Lynch syndrome, there is still a possibility of Lynch syndrome, although sporadic CRC is more likely. Continued surveillance with attention to personal and family history is recommended.
- No specific surveillance is required in cases of MSS/MSI-Low or those with no loss of MMR protein expression and a low likelihood of Lynch syndrome based on personal and family history. However, patients should seek medical attention if they develop symptoms suggestive of CRC or other Lynch syndrome-associated tumors or if new associated tumors develop in a relative.

### Genetic counseling and management of relatives

- The patient’s relatives should receive genetic counseling.
- At-risk family members (particularly first-degree relatives, such as parents, children, and siblings) should be provided with adequate information about the condition and, with their consent, undergo genetic testing or surveillance for associated tumors based on their risk assessment.
- In principle, considering that Lynch syndrome-associated tumors generally develop in adulthood, genetic testing should be performed in adult patients.

#### Patients with a confirmed genetic diagnosis

**Fig. 19:**
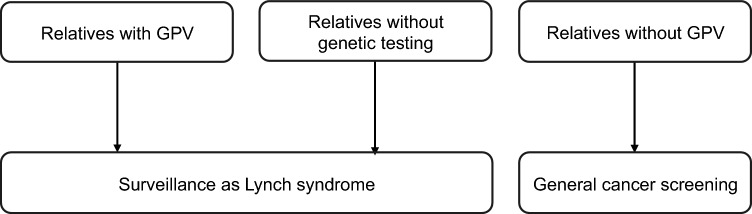
Management of biological relatives of patients with Lynch syndrome. GPV, germline pathogenic variant

Surveillance for Lynch syndrome-associated tumors is indicated for relatives confirmed to carry GPVs and those who have not undergone genetic testing (Fig. 19).

- Relatives who do not carry GPVs should undergo general screening for cancer.
- Information should be provided on the need for surveillance and the significance of genetic testing for relatives who have reached the age at which surveillance for Lynch syndrome-associated tumors should start. The decision to receive genetic counseling followed by genetic testing should be made on an individual basis.

#### Patients with unconfirmed genetic status

In relatives of patients who have not undergone genetic testing or in whom genetic testing have failed to yield a diagnosis of Lynch syndrome, individual risk should be assessed based on considerations such as age of onset and the incidence of Lynch syndrome-associated tumors in family members. Surveillance for associated tumors should be conducted.

## Clinical questions

### CQ6: Should universal screening for mismatch repair deficiency be performed in patients with CRC to screen for Lynch syndrome?

(**Evidence level: C**, **Recommendation level: 1**, **Agreement rate: 94.4%).**

**Recommendation:** Universal screening for Lynch syndrome in patients with CRC is strongly recommended.

Comments

A meta-analysis, four prospective cohort studies, nine retrospective studies, four guidelines, and one article identified by hand searching were reviewed to determine the efficacy and cost-effectiveness of universal screening for diagnosis of Lynch syndrome in all patients with CRC. Screening methods based on clinicopathological information, such as the Amsterdam criteria II and the revised Bethesda guidelines, have been used to screen for Lynch syndrome. However, 12%–28% of patients with Lynch syndrome could not be identified even by the revised Bethesda guidelines, which provide sensitive clinicopathological criteria [298, 299, 311]. Furthermore, accurately obtaining clinicopathological information, including a family history of cancer, is often challenging [415]. An integrated analysis of four large cohort studies that together included 10,206 patients with CRC found that 3.1% of the patients had Lynch syndrome but only 2.5% fulfilled the Amsterdam criteria II [298]. Specificity was high at 97.9% and sensitivity was low at 27.2%. However, 39.8% of patients met the revised Bethesda guidelines criteria, with high sensitivity of 88.1%, although specificity was low at 54.4% These findings suggest that screening for Lynch syndrome based on clinicopathological factors has limitations in terms of sensitivity, and balancing sensitivity and specificity is challenging.

MSI testing and MMR-IHC are now widely used for universal screening in patients with CRC in Europe and the USA. Universal screening is more expensive per diagnosis of Lynch syndrome than screening with the Amsterdam criteria II or the revised Bethesda guidelines [416]; however, universal screening does not rely on clinicopathological information and has extremely high sensitivity for a diagnosis of Lynch syndrome. The results of cost-effectiveness analyses that consider quality-adjusted life years gained also support use of universal screening [417–419]. Accordingly, guidelines from other countries recommend universal screening for all patients with CRC [9, 207, 229].

Notably, no cost-effectiveness analysis with Lynch syndrome has been conducted in Japan. The proportion of patients with Lynch syndrome among patients with CRC in Japan is approximately 1% [292, 293, 420, 421], which is lower than the 2.2% reported in a recent global meta-analysis [422]. Therefore, the cost-effectiveness of universal screening in Japan may be lower than that in Western countries. From a cost-effectiveness perspective, limiting universal screening to patients with CRC below a certain age, such as 70 years, has also been proposed, considering that Lynch syndrome is less common in older patients with CRC [423].

In theory, universal screening can detect cases of Lynch syndrome with high sensitivity; however, in reality, the sensitivity of screening is affected by various factors, including the rate of uptake of genetic testing by patients. Providing genetic counseling for patients with MMR deficiency detected by screening increases the rate of uptake of genetic testing [424]. Therefore, it is essential to establish a system that provides appropriate genetic counseling if universal screening is implemented to ensure diagnosis of Lynch syndrome with high sensitivity.

MSI testing and MMR-IHC can identify Lynch syndrome with similarly high sensitivity [425]. Screening by MMR-IHC with additional testing for the *BRAF* V600E variant can help to exclude sporadic MSI-H CRC in some cases (Fig. 17) and narrow down the target genes for genetic testing, making it more cost-effective than MSI testing [418].

### CQ7: Should colonoscopy surveillance in patients with Lynch syndrome be personalized based on the causative gene?

(**Evidence level: C, Recommendation level: 1, Agreement rate: 100%).**

**Recommendation:** It is strongly recommended that the causative gene should be considered when performing colonoscopy surveillance in patients with Lynch syndrome.

Comments

Two prospective cohort studies that examined the incidence of colorectal adenoma in patients with Lynch syndrome were identified [426, 427]. The prevalence of colorectal adenoma was between 10.6% and 23% and increased with age. Colonoscopy surveillance in Lynch syndrome has been reported to reduce mortality from CRC by 60%–72% and is the only effective surveillance method [336, 428]. Two prospective and eight retrospective cohort studies that analyzed the cumulative incidence of CRC based on the causative gene in patients with Lynch syndrome found that the cumulative incidence of CRC by the age of 80 years was 46%–61% for *MLH1*, 33%–52% for *MSH2* and *EPCAM*, 10%–44% for *MSH6*, and 8.7%–20% for *PMS2*, indicating variability in the incidence of CRC depending on the causative gene [10, 335, 410, 429–433]. Although performed retrospectively, an international collaborative study that included 5255 cases also reported a difference in the incidence of CRC depending on the causative gene, as well as differences according to sex and continent [434]. Japanese researchers participated in that study; however, comparisons with other continents were not possible because of the limited number of cases from Asia. Based on the findings to date, colonoscopy surveillance for CRC in patients with Lynch syndrome should take into account the causative gene and the sex of the patient.

Five prospective cohort studies, six retrospective studies, one meta-analysis, and four guidelines were reviewed to determine the age at which colonoscopy surveillance should start. The annual incidence of CRC in patients with Lynch syndrome in their 20 s has been reported to be < 1% regardless of the causative gene, and the incidence of CRC before the age of 25 years is extremely low [10, 315, 316, 353, 410, 429, 433, 436–444]. The cumulative incidence of CRC by the age of 30 years was found to be 0%–2.4% for *MLH1*, 0.4%–3.0% for *MSH2*/*EPCAM*, 0%–0.05% for *MSH6*, and 0.02%–0.1% for *PMS2* in females. In contrast, the cumulative incidence of CRC was 0.9%–4.5% for *MLH1*, 0.7%–2.6% for *MSH2*/*EPCAM*, 0%–0.5% for *MSH6*, and 0.2%–0.4% for *PMS2* in males. Furthermore, the cumulative incidence of CRC by the age of 40 years was 0.09%–2.5% for *MSH6* and 0.07–0.7% for *PMS2* in females and 1.2%–9.9% for *MSH6* and 0.5%–2.1% for *PMS2* in males [434]. Although the incidence rates were higher in males for all causative genes, the sex-related differences were not sufficiently significant to affect planning of surveillance. Therefore, in patients with Lynch syndrome, colonoscopy surveillance should begin at the age of 20–25 years for those with *MLH1* or *MSH2* as the causative gene and at 30–35 years for their counterparts with *MSH6* or *PMS2* as the causative gene when the onset of CRC is late. However, while the evidence for starting surveillance earlier is limited and should be determined on a case-by-case basis, surveillance may start 2–5 years earlier than the age of the youngest affected family member [9].

One prospective cohort study and two retrospective observational studies investigated the interval for colonoscopy surveillance. One observational study that included patients with Lynch syndrome and *MLH1*, *MSH2*, or *MSH6* as the causative gene reported that the cumulative incidence of CRC and the stage at diagnosis were similar regardless of whether lower gastrointestinal endoscopy was performed once a year, twice a year, or at 2–3-year intervals [338]. A prospective cohort study found that 69% of CRCs occurred more than 2 years after the last lower gastrointestinal endoscopy [10].

Studies from Europe and the USA have reported high cost-effectiveness for gene-specific colonoscopy surveillance in patients with Lynch syndrome [445, 446]. Starting colonoscopy surveillance at the age of 25 years rather than at 20 years in patients with Lynch syndrome and *MLH1* or *MSH2* as the causative gene and at the age of 35 or 40 years for those with *MSH6* or *PMS2* as the causative gene is more cost-effective. Furthermore, a 3-year surveillance interval is more cost-effective for patients who have Lynch syndrome with *MSH6* or *PMS2* as the causative gene. However, in Japan, no cost-effectiveness analysis has been reported regarding the cost of colonoscopy surveillance or the cumulative CRC incidence rate for each causative gene.

Based on these findings, the interval for colonoscopy surveillance should be every 1–2 years in patients who have Lynch syndrome with *MLH1*, *MSH2*/*EPCAM*, or *MSH6* as the causative gene and every 1–3 years for those with *PMS2* as the causative gene. Patients with a history of CRC or adenoma, those who are male, those with *MLH1* or *MSH2* as the causative gene, and those over 40 years of age should undergo annual surveillance [10, 447].

### CQ8: Is chemoprevention as effective as surveillance in patients with Lynch syndrome?

(**Evidence level: B, Recommendation level: 2, Agreement rate: 100%).**

**Recommendation:** It is weakly recommended that aspirin should not be administered for cancer chemoprevention in patients with Lynch syndrome.

Comments

Five randomized controlled trials that examined the effectiveness and safety of chemoprevention in patients with Lynch syndrome were reviewed, the most well-known being the CAPP2 trial conducted in Europe and the USA [448]. CAPP2 was a double-blind study that evaluated the ability of aspirin 600 mg/day and resistant starch 30 g/day to prevent Lynch syndrome-associated tumors and colorectal adenomas or cancers in patients with Lynch syndrome. After 10 years of follow-up, the group that received aspirin for longer than 2 years showed a significant reduction in the incidence of CRC when compared with the placebo group (HR, 0.65; 95% CI 0.43–0.97) [449]. Furthermore, the risk of CRC increased by 7% for every 1-unit increase in body mass index in the placebo group; however, no increase in risk was found in the aspirin-treated group [342]. In CAPP2, the number of evaluable participants decreased by approximately 40% over 20 years, so caution is required when interpreting the data from this trial.

Long-term use of aspirin increases the risk of gastrointestinal complications and is contraindicated after 28 weeks of pregnancy. Furthermore, studies in the general population have suggested that the effect of aspirin on the risk of CRC may depend on body weight, indicating that the benefits of a fixed dose may be outweighed by the risks if the individual is not within the optimal weight range [450]. Therefore, it is weakly recommended that aspirin should not be administered for cancer chemoprevention in patients with Lynch syndrome at this time. The randomized non-inferiority CAPP3 study is presently underway to determine the optimal aspirin dose, comparing 600 mg/day with 300 mg/day and 100 mg/day in patients with Lynch syndrome.

The final follow-up results from CAPP2 showed that treatment with resistant starch did not affect the incidence of CRC [451]. However, resistant starch reduced the risk of non-colorectal Lynch syndrome-associated cancers (particularly in the stomach, duodenum, bile duct, and pancreas) by 46% (HR, 0.54; 95% CI 0.33–0.86). The mechanism by which resistant starch reduces the risk of non-colorectal cancers is not yet understood. Therefore, based solely on the findings of CAPP2, it is not recommended to routinely consume resistant starch to reduce the risk of non-colorectal Lynch syndrome-associated cancers. Moreover, insufficient evidence exists for the use of sulindac, celecoxib, or ibuprofen.

### CQ9: Is risk-reducing surgery (i.e., hysterectomy or bilateral salpingo-oophorectomy) beneficial for patients with Lynch syndrome?

(**Evidence level: C, Recommendation level: None, Agreement rate: 94.4%).**

**Recommendation:** Although risk-reducing surgery in women with Lynch syndrome does not reduce mortality, it should be considered as an option to reduce the risk of endometrial and ovarian cancers. However, this option should be considered on a case-by-case basis according to factors such as the desire for childbearing, complications, family history of Lynch syndrome-related tumors, and the type of MMR gene involved.

Comments

**1. Tumor suppression:** Two case–control studies and one prospective cohort study were reviewed. The case–control studies indicated that hysterectomy and bilateral salpingo-oophorectomy reduce the risk of developing endometrial and ovarian cancers. According to a report on risk-reducing surgery published by Schmeler et al., none of 61 women who underwent hysterectomy developed endometrial cancer during an average postoperative follow-up of 13.3 years [383]. In contrast, 33% of 210 women who did not undergo hysterectomy developed endometrial cancer during an average follow-up of 7.4 years. Similarly, none of the 47 women who underwent bilateral salpingo-oophorectomy developed ovarian cancer after an average follow-up of 11.2 years. In contrast, 5.5% of 223 women who did not undergo bilateral salpingo-oophorectomy developed ovarian cancer during an average follow-up of 10.6 years. Four of 41 women who underwent risk-reducing surgery (hysterectomy plus bilateral salpingo-oophorectomy, n = 32; hysterectomy alone, n = 7; bilateral salpingo-oophorectomy alone, n = 2) had endometrial cancer/hyperplasia in the surgical specimens. In contrast, nine of the 45 women who did not undergo risk-reducing surgery developed endometrial cancer/hyperplasia and two developed ovarian cancer during surveillance [452]. The international multicenter Prospective Lynch Syndrome Database (PLSD) study, which was performed primarily in Europe, investigated the age-specific effects of risk-reducing surgery on the risks of endometrial and ovarian cancers in women with Lynch syndrome [453]. For women who underwent risk-reducing hysterectomy at the age of 25 years, the risk of developing endometrial cancer by the age of 70 years was reduced by 35% in carriers of the *MLH1* variant, by 47% in *MSH2* carriers, by 41% in *MSH6* carriers, and by 13% in *PMS2* carriers. Similarly, for those who underwent risk-reducing hysterectomy at the age of 40 years, the risk reduction by the age of 70 years was 34% for *MLH1* carriers, 45% for *MSH2* carriers, 40% for *MSH6* carriers, and 13% for *PMS2* carriers. Therefore, the effect of risk-reducing hysterectomy on the risk of endometrial cancer is similar whether performed at the age of 25 years or 40 years, and there is little benefit in performing the surgery before the age of 40 years. Moreover, for women who underwent risk-reducing bilateral salpingo-oophorectomy at the age of 25 years, the risk of developing ovarian cancer by the age of 70 years was reduced by 11% for carriers of the *MLH1* variant, 17% for *MSH2*, 11% for *MSH6*, and 3% for *PMS2*. Similarly, the risk reduction by the age of 70 years was 9% for *MLH1*, 16% for *MSH2*, 9% for *MSH6*, and 3% for *PMS2* among those who underwent the procedure at the age of 40 years. Therefore, the effect on the risk of ovarian cancer is similar whether the surgery is performed at the age of 25 years or 40 years, and the benefits of undergoing the surgery before 40 years of age are limited.

**2. Improvement of prognosis:** No study has directly investigated the effectiveness of risk-reducing surgery on prognosis. Two prospective cohort studies [10, 454] reported that women with Lynch syndrome who underwent surveillance without preventive hysterectomy or bilateral salpingo-oophorectomy had a relatively good prognosis, even if they developed endometrial or ovarian cancer. Among 123 women diagnosed with Lynch syndrome (The surveillance compliance rate was 97.1%), 19 of 103 patients (18%) who underwent surveillance using endometrial biopsy and transvaginal ultrasound every 2–3 years starting at the age of 35 years developed endometrial cancer (median age 49 years). Only 2 of these 19 women were symptomatic at diagnosis, and no deaths were observed during a median follow-up of 8 years. Ovarian cancer occurred in 6 of 112 women, with no deaths during a median follow-up of 2 years [454]. In a prospective cohort study of data from the PLSD, which allowed for both surveillance and risk-reducing surgery during the study period, 186 of 1057 women with Lynch syndrome underwent hysterectomy and 153 underwent bilateral salpingo-oophorectomy. Among those who remained under surveillance, 72 developed endometrial cancer, with a 10-year survival rate of 98%. Furthermore, 19 women developed ovarian cancer, with a 10-year survival rate of 88%, indicating a relatively favorable prognosis [10]. The PLSD study also showed that risk-reducing bilateral salpingo-oophorectomy before the age of 40 years does not improve life expectancy, particularly in carriers with PGVs in *MSH6* and *PMS2* [453].

**3. Improved cost-effectiveness:** Health economics studies from Europe and the USA have reported that risk-reducing surgery in women with Lynch syndrome is cost-effective [455–457]. However, careful consideration is required before its implementation in Japan.

**4. Psychological impact and quality of life:** Two questionnaire surveys of women with Lynch syndrome who underwent risk-reducing surgery (hysterectomy ± bilateral salpingo-oophorectomy) found that although psychological anxiety related to cancer was reduced, there were concerns regarding the effects of iatrogenic menopause [458, 459].

**5. Perioperative complications:** In one study, serious complications occurred in one (1.6%) of 61 women who underwent risk-reducing surgery [383]. Notably, this case had a history of surgery and radiation therapy for rectal cancer.

### CQ10: Is screening for HP infection beneficial in patients with Lynch syndrome?

(**Evidence level: C, Recommendation level: 2, Agreement rate: 88.9**%).

**Recommendation:** It is weakly recommended that screening for HP infection should be performed in patients with Lynch syndrome to reduce the risk of gastric cancer.

Comments

We reviewed one prospective observational cohort study, nine retrospective cohort studies, one retrospective observational study, three reviews, and one guideline that examined the significance of upper gastrointestinal endoscopy and HP testing for the surveillance of gastric cancer in Lynch syndrome. The risk of gastric cancer in the general population is higher in East Asia than in Western countries. Similarly, the risk of developing gastric cancer is higher in East Asian patients with Lynch syndrome than in their Western counterparts. The HP infection rate largely influences this geographic difference in the risk of gastric cancer. HP infection leads to chronic inflammation of the stomach, resulting in atrophic gastritis and intestinal metaplasia, which increase the risk of intestinal-type (differentiated) gastric adenocarcinoma [460]. Therefore, this guideline considers geographic differences and examines the recommendation for screening for HP infection and subsequent eradication therapy in patients with Lynch syndrome.

The lifetime risk of developing gastric cancer in patients with Lynch syndrome has been reported to be up to 13% in Western countries [316, 335], but is as high as 41% in East Asia [11, 404, 462], indicating a higher lifetime risk of gastric cancer in East Asians. Risk factors for gastric cancer or premalignant lesions in Lynch syndrome include male sex, advanced age, and more severe atrophic gastritis [316, 335, 463]. Furthermore, the PLSD study, which analyzed data primarily from Europe, found that the risk of developing gastric cancer varied depending on the causative gene. The cumulative relative risk of developing gastric cancer by the age of 75 years in carriers of pathogenic variants in *MLH1* or *MSH2* is 8.9 and 9.7 times higher, respectively, than in the general population [410]. However, no correlation has been found between a history of gastric cancer in a first-degree relative and the risk of developing gastric cancer in the proband [464].

Studies from Western countries have not demonstrated an association of development of gastric cancer with chronic gastritis or HP infection in patients with Lynch syndrome [465, 466]. However, many of the gastric cancers associated with Lynch syndrome are of the intestinal type [280, 403]. In contrast, a retrospective cohort study from Japan indicated that atrophic gastritis is a risk factor for development of gastric cancer in patients with Lynch syndrome [404]. Another retrospective cohort study from Japan also reported that all six gastric cancer specimens in patients with Lynch syndrome who were tested for HP were infected [11]. Therefore, although there is no direct evidence of involvement of HP infection in the development of gastric cancer in Lynch syndrome, the presence of atrophic gastritis, the high rate of HP infection in gastric cancers that do develop, and the prevalence of intestinal-type gastric adenocarcinoma suggest that HP infection is a risk factor for gastric cancer in Lynch syndrome [9].

In view of the evidence to date, the recommendation for screening tests to confirm the presence of HP infection in the surveillance for gastric cancer in patients with Lynch syndrome in addition to upper gastrointestinal endoscopy is weak. Eradication therapy should be considered if HP infection is confirmed.

## Data Availability

No new data were generated or analyzed in the development of this guideline. The recommendations are based on previously published studies, which are cited within the guideline document.
